# Gene network activity in cultivated primary hepatocytes is highly similar to diseased mammalian liver tissue

**DOI:** 10.1007/s00204-016-1761-4

**Published:** 2016-06-23

**Authors:** Patricio Godoy, Agata Widera, Wolfgang Schmidt-Heck, Gisela Campos, Christoph Meyer, Cristina Cadenas, Raymond Reif, Regina Stöber, Seddik Hammad, Larissa Pütter, Kathrin Gianmoena, Rosemarie Marchan, Ahmed Ghallab, Karolina Edlund, Andreas Nüssler, Wolfgang E. Thasler, Georg Damm, Daniel Seehofer, Thomas S. Weiss, Olaf Dirsch, Uta Dahmen, Rolf Gebhardt, Umesh Chaudhari, Kesavan Meganathan, Agapios Sachinidis, Jens Kelm, Ute Hofmann, René P. Zahedi, Reinhard Guthke, Nils Blüthgen, Steven Dooley, Jan G. Hengstler

**Affiliations:** 1 IfADo-Leibniz Research Centre for Working Environment and Human Factors, Technical University of Dortmund, Ardeystrasse 67, 44139 Dortmund, Germany; 2 Leibniz Institute for Natural Product Research and Infection Biology eV-Hans-Knöll Institute, Jena, Germany; 3 Molecular Alcohol Research in Gastroenterology, Department of Medicine II, Faculty of Medicine at Mannheim, University of Heidelberg, Mannheim, Germany; 4 BG Trauma Center, Siegfrid Weller Insitut, Eberhard Karls University Tübingen, Tübingen, Germany; 5 Center for Liver Cell Research, Department of General, Visceral, Transplantation, Vascular and Thorax Surgery, Grosshadern Hospital, Ludwig Maximilians University, Munich, Germany; 6 Department of General, Visceral and Transplantation Surgery, Charité University Medicine Berlin, Berlin, Germany; 7 Center for Liver Cell Research, University Children Hospital (KUNO), Regensburg University Hospital, Regensburg, Germany; 8 Institute of Pathology, Friedrich-Schiller-University of Jena, Jena, Germany; 9 Experimental Transplantation Surgery, Department of General, Visceral and Vascular Surgery, University Hospital Jena, Friedrich-Schiller-University of Jena, Jena, Germany; 10 Institute of Biochemistry, Faculty of Medicine, University of Leipzig, Leipzig, Germany; 11 Institute of Neurophysiology, Medical Faculty, University of Cologne, Cologne, Germany; 12 InSphero AG, Wagistrasse 27, 8952 Schlieren, Switzerland; 13 Dr. Margarete Fischer-Bosch Institute of Clinical Pharmacology, University of Tuebingen, Auerbachstrasse 112, 70376 Stuttgart, Germany; 14 Leibniz-Institut für Analytische Wissenschaften - ISAS - e.V., Dortmund, Germany; 15 Institute of Pathology, Charité-Universitätsmedizin Berlin, Berlin, Germany; 16 Department of Forensic and Veterinary Toxicology, Faculty of Veterinary Medicine, South Valley University, Qena, Egypt; 17 Facultad de Ciencias Biológicas, Departamento de Fisiología, Universidad de Concepción, Concepción, Chile; 18 Department of Developmental Biology, Washington University School of Medicine, St. Louis, MO USA

**Keywords:** Gene arrays, Bioinformatics, Inflammation, Metabolism, Differentiation

## Abstract

It is well known that isolation and cultivation of primary hepatocytes cause major gene expression alterations. In the present genome-wide, time-resolved study of cultivated human and mouse hepatocytes, we made the observation that expression changes in culture strongly resemble alterations in liver diseases. Hepatocytes of both species were cultivated in collagen sandwich and in monolayer conditions. Genome-wide data were also obtained from human NAFLD, cirrhosis, HCC and hepatitis B virus-infected tissue as well as mouse livers after partial hepatectomy, CCl_4_ intoxication, obesity, HCC and LPS. A strong similarity between cultivation and disease-induced expression alterations was observed. For example, expression changes in hepatocytes induced by 1-day cultivation and 1-day CCl_4_ exposure in vivo correlated with *R* = 0.615 (*p* < 0.001). Interspecies comparison identified predominantly similar responses in human and mouse hepatocytes but also a set of genes that responded differently. Unsupervised clustering of altered genes identified three main clusters: (1) downregulated genes corresponding to mature liver functions, (2) upregulation of an inflammation/RNA processing cluster and (3) upregulated migration/cell cycle-associated genes. Gene regulatory network analysis highlights overrepresented and deregulated HNF4 and CAR (Cluster 1), Krüppel-like factors MafF and ELK1 (Cluster 2) as well as ETF (Cluster 3) among the interspecies conserved key regulators of expression changes. Interventions ameliorating but not abrogating cultivation-induced responses include removal of non-parenchymal cells, generation of the hepatocytes’ own matrix in spheroids, supplementation with bile salts and siRNA-mediated suppression of key transcription factors. In conclusion, this study shows that gene regulatory network alterations of cultivated hepatocytes resemble those of inflammatory liver diseases and should therefore be considered and exploited as disease models.

**Electronic supplementary material:**

The online version of this article (doi:10.1007/s00204-016-1761-4) contains supplementary material, which is available to authorized users.

## Introduction

Hepatocyte in vitro systems represent a well-accepted tool in many fields of basic and applied research such as pharmacology and toxicology, tissue engineering and clinical hepatocyte transplantation (Godoy et al. 2013). However, despite of their widespread use, research with primary hepatocytes remains challenging (Godoy et al. 2013). Isolating hepatocytes from their physiological environment in the liver causes alterations in cell physiology and major gene expression alterations (Godoy et al. 2013; Zellmer et al. 2010). However, it has never been studied whether these changes only represent in vitro artifacts or whether they resemble disease-relevant processes. Such a situation has since long been acknowledged for liver fibrosis, where cultivated stellate cells undergo similar activation mechanisms as in the fibrotic liver (De Minicis et al. 2007). To address this question, we compared time-resolved, genome-wide data of cultivated hepatocytes and liver disease models. We report that alterations in cultivated human and mouse hepatocytes resemble those in inflammatory liver diseases, and that similar transcriptional networks and transcription factors are responsible for the identified changes. Since we performed the study under identical conditions for mouse and human hepatocytes, a systematic interspecies comparison was possible, identifying similar features such as HFN4-driven downregulation of metabolic functions, but also major interspecies differences such as Klf6-driven inflammatory processes. The resulting transcriptomic network directory offers a blueprint for interventions for improving the in vitro systems but also for interfering with disease-relevant processes.

## Materials and methods

### Hepatocyte isolation and cultivation

Primary mouse hepatocytes were isolated from male C57BL6/N mice (8–12 weeks old) by the two-step collagenase perfusion method (Godoy et al. 2013). Primary human hepatocytes were obtained under informed consent from patients undergoing surgical liver resection by a two-step collagenase I perfusion (Godoy et al. 2013). The cells were plated on six-well dishes, either onto dried collagen I (monolayer) or between two layers of soft-gel collagen (sandwich) as described in (Godoy et al. 2013). Details for the protocols can be found in the supplemental section.

### Genome-wide analyses and bioinformatics

Affymetrix gene array analysis was performed as previously described (Godoy et al. 2009), using the Affymetrix GeneChip^®^ Mouse Genome A430 2.0 (Santa Clara, CA, USA) (details in suppl. Section). Affymetrix gene expression data were preprocessed using ‘affyPLM’ packages of the Bioconductor software as previously described (Godoy et al. 2015). Data obtained from fresh hepatocytes were used as reference. A false positive rate of *a* = 0.05 with FDR correction and a fold change greater 2 were taken as the level of significance. Two samples, one *M*_S_ day 1 and one *S* day 5, were identified as outliers by principal component and Pearson’s correlation analyses and were not included in the subsequent analyses (Suppl. Fig. 1; Suppl. Table 1). A list with all differentially regulated genes (DEG) in hepatocytes and liver disease models can be found in the supplemental section. Processing and visualization (principal component analysis) of data were performed using MATLAB tools (The MathWorks Inc., Natick, MA, USA). Clusters of correlated genes based on similar time-dependent fold change were generated by fuzzy c-means. A list with genes belonging to each fuzzy cluster is provided in the supplemental section. Gene set enrichment analysis (GSEA) was performed using the manually curated Gene Ontology of the BIOBASE Knowledge Library (BKL) of the ExPlain™ Web service (BIOBASE GmbH, Wolfenbüttel, Germany). Overrepresented transcription factors binding sites were identified using the algorithm PRIMA (PRomoter Integration in Microarray Analysis) of the Expander software 6.1 (EXPression ANalyzer and DisplayER) as previously described (Godoy et al. 2015). Metagenes were generated by calculating the mean expression value for each biological sample in any model system for each set of genes that belong to the respective metagene (Schmidt et al. 2008). Interspecies (mouse/human) gene expression comparison was performed by using orthologous genes (Yue et al. 2014). Correlation analyses (Spearman’s rank correlation and odds ratio) were performed using Statistics Toolbox of MATLAB. A classification probability and a metric of the gene regulatory networks (GRN) status, related to 16 specific mouse and human tissues and cells, were calculated using the CellNet platform (Cahan et al. 2014), using the locally available R version of the software CellNet http://pcahan1.github.io/cellnetr/.

### Additional methods and gene expression profiles from mouse and human cell lines and disease models

Additional methods such in vivo models of liver disease, flow cytometry, Percoll-based purification, immunofluorescence, siRNA-mediated knockdown of Klf6 and MafF and LUMINEX assays, together with a complete list of chemicals, reagents, antibodies and tables containing gene expression data and bioinformatics, are provided in the supplemental section. Additional gene expression data (based on Affymetrix gene arrays) including mouse HCC (Dapito et al. 2012), mouse cell lines AML12 (Ventura-Holman et al. 2008) and iHep (stem cell-derived hepatocytes) (Morris et al. 2014), human cell lines HepG2 (Rodrigues et al. 2016) and HLC (stem cell-derived hepatocytes) (Godoy et al. 2015), human hepatitis B-infected liver (Farci et al. 2010), human non-alcoholic fatty liver (NAFLD) (Moylan et al. 2014), human cirrhosis and hepatocellular carcinoma (HCC) (Yildiz et al. 2013) were obtained from Gene Expression Omnibus http://www.ncbi.nlm.nih.gov/geo/ or ArrayExpress https://www.ebi.ac.uk/arrayexpress/ and provided in the supplemental section.

## Results

### Stereotypic gene expression responses to different types of stress

**Fig. 1:**
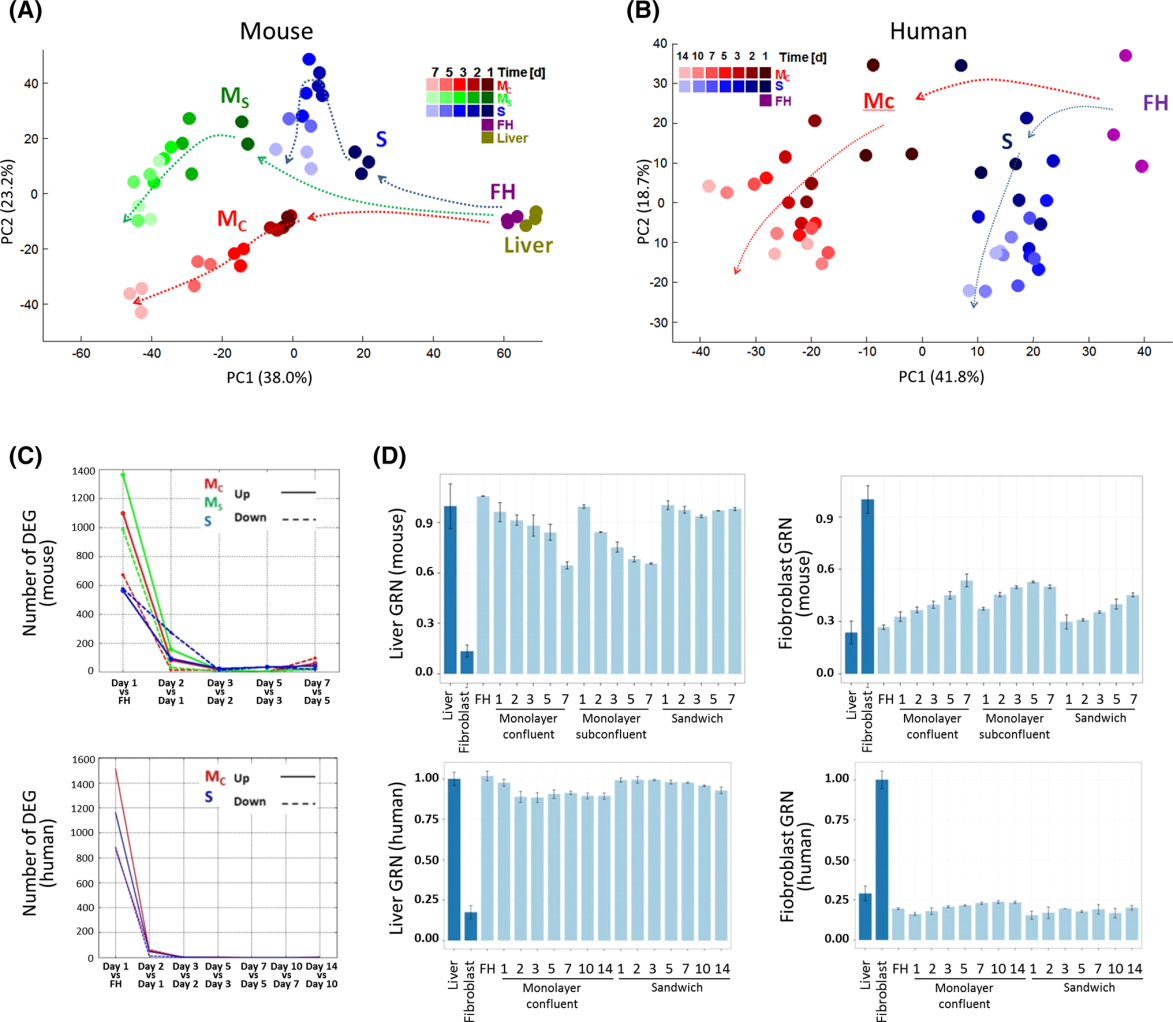
Transcriptional alterations induced by cultivation of primary hepatocytes. **a**, **b** Principal component analysis representing the 1000 genes with highest variance in primary mouse (**a**) and human (**b**) hepatocytes. The *top* two principal components (PC1 and PC2) represent 61.2 % of the variance in **a** and 60.5 % in **b**. The *graph* includes primary hepatocytes in 2D monolayer confluent (*M*_C_) or subconfluent (*M*_S_), and 3D (sandwich; *S*) cultures. The largest deviations from the reference state freshly isolated hepatocytes (FH) occur during the first 24 h of culture in both mouse and human hepatocytes. Moreover, liver tissue (liver) was included into the expression profiling study. A cultivation period from day 1 to day 14 was analyzed for humans. Analysis of the faster dedifferentiating mouse hepatocytes was already terminated at cultivation day 7. The *dashed arrows* indicate the trend of gene expression changes in time, in relation to freshly isolated primary hepatocytes. **c** Deregulated genes in a day-by-day analysis of mouse and human hepatocytes. The number of differentially expressed genes (DEGs) is maximally induced at 24 h in culture (‘Day 1 vs FH’). **d** Cell identity analysis based on gene regulatory network scores (GRN) in freshly isolated and cultivated primary mouse and human hepatocytes. The ‘liver’ GRN score (maximal in fresh hepatocytes) is progressively suppressed during cultivation of mouse hepatocytes in monolayer culture. Sandwich cultures in mouse and human hepatocytes more efficiently sustain the ‘liver’ score. The opposite trend is observed for the ‘fibroblast’ GRN score that increases during cultivation in monolayers, while sandwich cultures ameliorate this effect, whereby that stabilizing effect of collagen sandwiches is strongest in human hepatocytes. *Dark blue bars* represent the score of the training datasets for ‘liver’ and ‘fibroblast,’ respectively, and the *numbers below the bars* indicate the days in culture

To understand the structure of gene expression alterations in hepatocytes, we compared genome-wide expression changes that occur during cultivation to changes in liver diseases and experimental disease models. Human and mouse hepatocytes were isolated by similar techniques and cultivated under sandwich and monolayer conditions. Over the entire cultivation period, hepatocytes of both species showed the expected morphology (Suppl. Fig. 2). Genome-wide analysis identified the following features for hepatocytes of both species: (1) The by far largest gene expression alterations occurred during the first 24 h in culture [Fig. 1a–c; Suppl. Tables 2–4; genes in human hepatocytes in Godoy et al. (2015)]; (2) later, these genes continued to be up- or downregulated but at a lower rate. Only few additional genes were deregulated at day 2 or later (Fig. 1c). (3) Deregulated genes of all culture systems overlapped strongly (Suppl. Fig. 3). (4) The total numbers of deregulated genes were lower in sandwich cultures compared to monolayers (Fig. 1c).

**Fig. 2:**
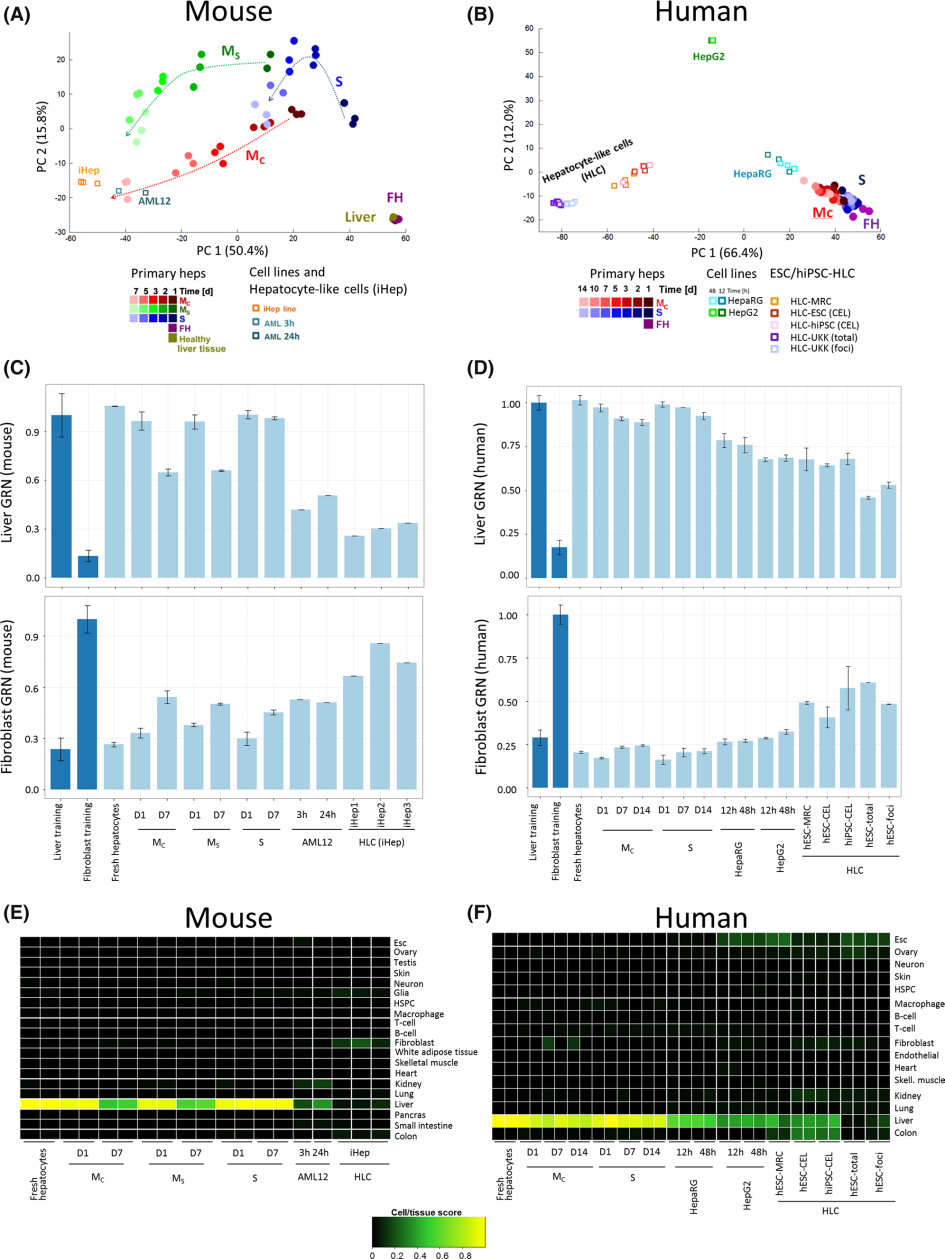
Comparison of primary hepatocytes in culture to hepatocyte cell lines and stem cell-derived hepatocyte-like cells. **a** Principal component analysis representing the 1000 genes with highest variance in cultivated mouse hepatocytes, the AML12 cell line at 3 and 24 h in culture and human-induced pluripotent stem cell-derived hepatocyte-like cells (iHep). For mouse, the time course expression alterations are indicated by *dashed arrows*. The *graph* shows the *top* two principal components (PC1 and PC2) representing 66.2 % of the variance. **b** Principal component analysis representing the 1000 genes with highest variance in cultivated human hepatocytes, the hepatoma cell lines HepaRG and HepG2 at 12 and 24 h in culture, and embryonic stem cell (ESC) as well as human-induced pluripotent stem cell (hiPSC)-derived hepatocyte-like cells (HLC). The *graph* shows the *top* two principal components (PC1 and PC2) representing 78.4 % of the variance. **c**, **d** Cell/tissue identity analysis based on gene regulatory networks (GRN) in freshly isolated and cultivated primary mouse (**c**) and human (**d**) hepatocytes, their corresponding cell lines and hepatocyte-like cells. For mouse and human cell lines, the ‘liver’ GRN scores are significantly lower than in long-term cultured primary hepatocytes. Similarly, the ‘fibroblast’ GRN scores are higher in all cell lines as in long-term cultured hepatocytes. **e**, **f** Cell/tissue identity analysis by CellNet in cultivated mouse (**e**) and human (**f**) primary hepatocytes, hepatocyte cell lines and stem cell-derived hepatocyte-like cells. The *heat maps* show the cell and tissue classification probability on freshly isolated primary hepatocytes (FH) and primary hepatocytes in monolayer confluent, subconfluent and sandwich culture for the indicated time (days). Cell/tissue identities based on gene expression profiles were analyzed with the CellNet algorithm (see supplemental methods) and compared to the training expression profiles defining 16 (human) or 20 (mouse) tissues or cells, as described in Cahan et al. (2014)

The major difference between mouse and human hepatocytes was that expression alterations (fold changes) were stronger in mouse than in human hepatocytes. For example, in monolayer cultures, 2498 and 2381 genes were deregulated at least twofold in mouse and human hepatocytes, respectively, whereas 117 versus 65 genes were above tenfold deregulated in mouse and human hepatocytes, respectively (*p* < 0.05, FDR adjusted) [Suppl. Tables 2–4, genes in human hepatocytes in Godoy et al. (2015)]. The massive gene expression changes (Fig. 1a, b) could mean that hepatocytes in culture lose their original cell identity or even adopt identities of other cell/tissue types. To study possible changes in cell identity in an unbiased manner, a cell/tissue score based on global gene expression profiles (i.e., gene regulatory network status—GRN) was calculated using the CellNet platform (Cahan et al. 2014) (Fig. 1d). In monolayer cultures of mouse hepatocytes, both *M*_C_ and *M*_S_ a progressive repression on ‘liver’ GRN scores during cultivation are observed, while in sandwich (*S*) cultures the ‘liver’ score was well maintained (Fig. 1d). The opposite trend was obtained for the ‘fibroblast’ GRN score (Fig. 1d) which may reflect the well-known EMT-like process induced in monolayer cultures (Dooley et al. 2008; Godoy et al. 2009). The ‘liver’ and ‘fibroblast’ GRN scores were much more stable in human hepatocytes compared to mice, and again *S* cultures generated more stable GRN scores for ‘liver’ and ‘fibroblast’ (Fig. 1d). All further studied cell/tissue identities did not show significant changes (Suppl. Fig. 4). Although the expression changes in cultivated primary hepatocytes are massive, the cells still resemble freshly isolated hepatocytes more closely than liver cell lines, AML12 (mice) and HepaRG as well as HepG2 (human) (Fig. 2a, b). Particularly for human hepatocytes, the difference between up to 14 days sandwich-cultured cells to the two cell lines and stem cell-derived hepatocyte-like cells (HLC) is large (Fig. 2b). The impression obtained by the principal component analysis (PCA) is confirmed by GRN scores for liver and fibroblasts (Fig. 2c, d). Also the cell/tissue scores confirm that the cell lines and HLC have lower hepatocyte scores and higher fibroblast scores, but do not gain features of other cell types (Fig. 2e, f).

### Similarity of global gene expression changes induced during hepatocyte cultivation and various liver diseases

**Fig. 3:**
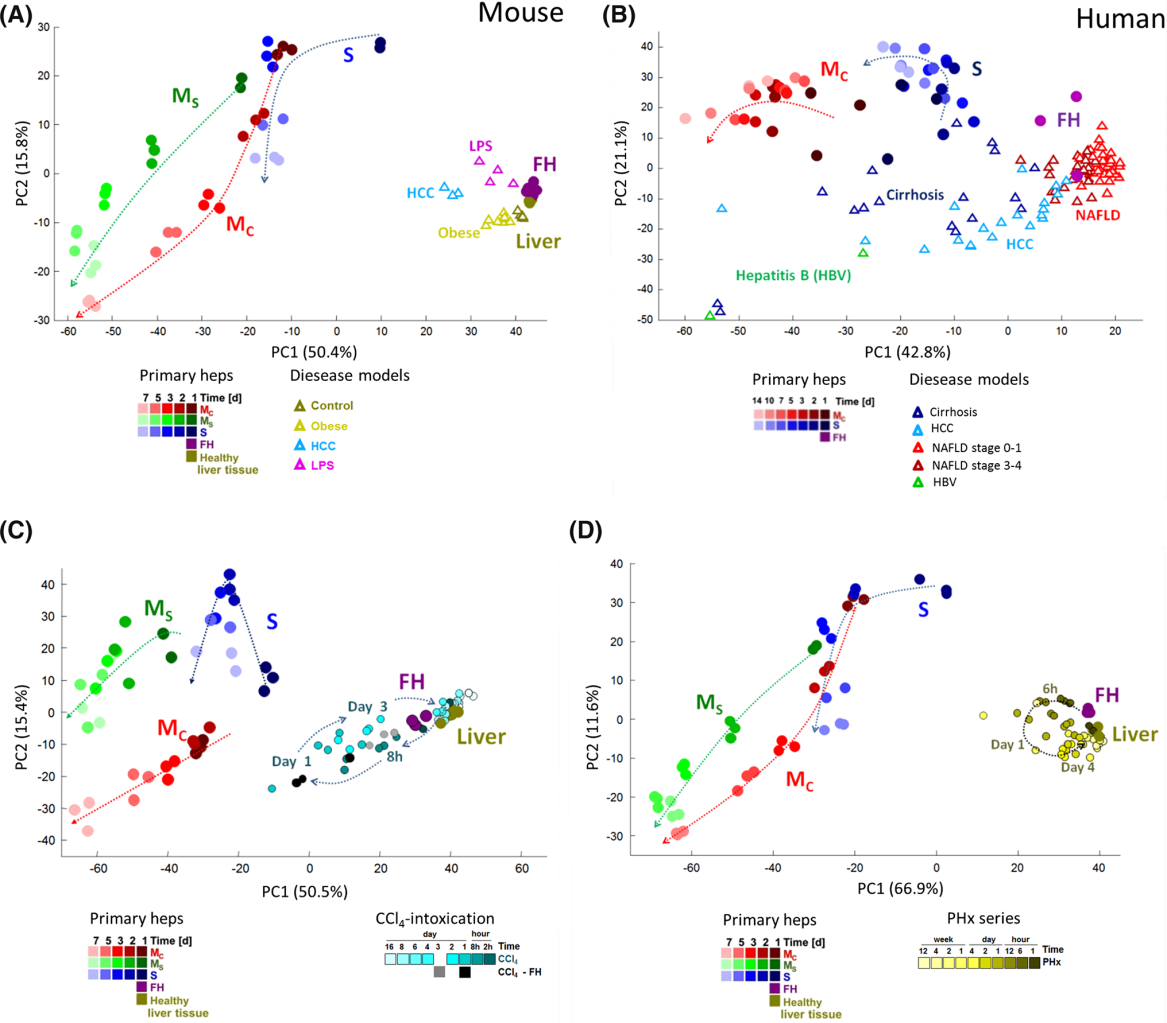
Similarity of transcriptional responses in cultivated hepatocytes and diseased or damaged livers. **a** Analysis of cultivated primary mouse hepatocytes (*M*_c_, *M*_s_, *S*), mouse liver tissue 24 h after intraperitoneal injection of LPS, hepatocellular carcinoma induced by a single diethylnitrosamine injection followed by chronic CCl_4_ intoxication (HCC, *n* = 3), steatosis in obese mice induced by leptin deficiency (ob/ob) and freshly isolated hepatocytes from healthy mice (FH). Principal components (PC1 and PC2) represent 66.2 % of the variance. **b** Analysis of human hepatocytes, liver tissue of patients with cirrhosis (*n* = 15), hepatocellular carcinoma (HCC, *n* = 15), non-alcoholic fatty liver disease (NAFLD) stages 0–1 (*n* = 40) and stages 3–4 (*n* = 32), and hepatitis B infection (*n* = 2). The *graph* shows the *top* two principal components (PC1 and PC2) representing 63.9 % of the variance. **c**, **d** Transcriptomic alterations in cultivated mouse hepatocytes compared to time course analysis of mouse liver tissue after acute liver damage by a single intoxication with CCl_4_ (**c**) and after 2/3 partial hepatectomy (**d**). The *dashed arrows* indicate the trend of gene expression changes in time, on cultivated hepatocytes and in liver tissue after CCl_4_ intoxication or partial hepatectomy. The *top* two principal components (in **c**, **d**) represent 65.9 and 78.5 % of the variance, respectively. All PCA graphs are based on the 1000 genes with highest variance

**Fig. 4:**
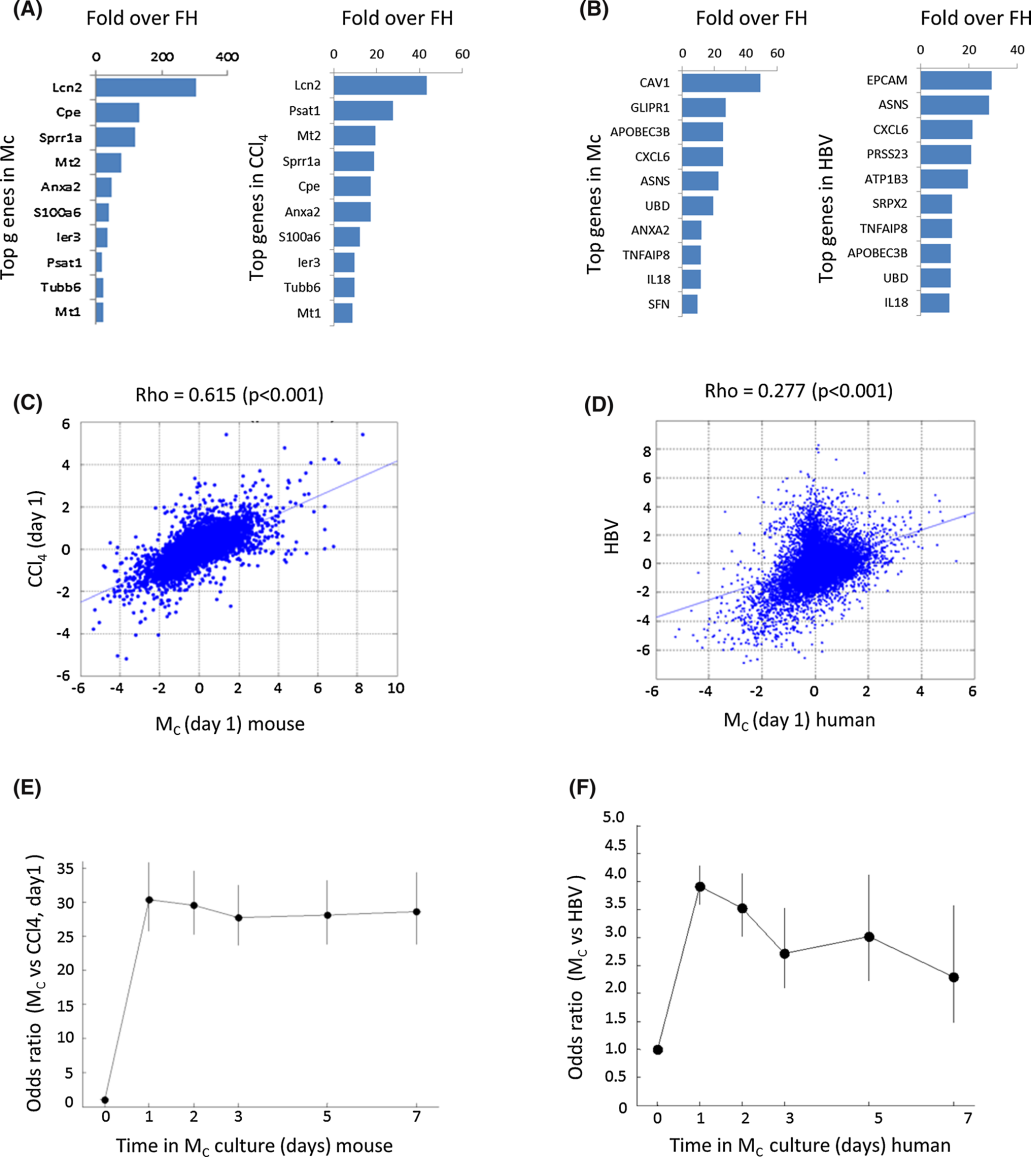
Similarities in transcriptional responses induced in cultivated hepatocytes and liver diseases. **a**, **b** *Top* ten upregulated genes in primary mouse (**a**) and human (**b**) hepatocytes in monolayer confluent (*M*_C_), and in mouse liver tissue on day 1 after CCl_4_ intoxication (**a**) or in HBV-infected human liver (**b**). The *bars* show fold changes over freshly isolated hepatocytes by gene array analysis. **c** *Scatter plots* showing all differentially expressed genes in primary mouse hepatocytes in monolayer confluent culture (day 1) and in mouse liver tissue on day 1 after CCl_4_ administration (log2 scale). **d** *Scatter plot* showing all differentially expressed genes in primary human hepatocytes on monolayer confluent culture (day 1) and in HBV-infected human liver tissue. **e** Odds ratio analysis between differentially expressed genes in primary mouse hepatocytes (at different days on monolayer confluent culture) and mouse liver tissue on day 1 after CCl_4_ administration. **f** Odds ratio analysis between differentially expressed genes in primary human hepatocytes (at different days on monolayer confluent culture) and mouse HBV-infected human liver tissue. Rho: correlation coefficient and its *p* value

In a next step, we compared cultivation-induced expression changes to those occurring in liver diseases. In mice, a shift inversely along PC1 was observed for HCC tissue, obese livers and livers of LPS-exposed mice (Fig. 3a). Also for humans, this move was seen for HCC, cirrhosis and hepatitis B virus-infected livers and parallels the cultivation-induced shift (Fig. 3b). In contrast, NAFLD did not show this shift. Moreover, we analyzed mouse liver tissue at various time periods after CCl_4_ intoxication (Fig. 3c) and partial hepatectomy (Fig. 3d). Again, an initial shift inversely along PC1 was seen up to day 1, followed by recovery at later time points. Although the changes obtained in vivo were smaller compared to the cultivation-induced alterations, the similarity in PCA orientation encouraged us to study these in further detail. We next focused on the strongest deregulated genes. The most upregulated gene in cultivated mouse hepatocytes is lipocalin 2 (Lcn2; 305-fold up at day 1 in *M*_C_; Fig. 4a; immunoblot validation in Suppl. Fig. 5), a known acute phase response gene in LPS-induced inflammation (Flo et al. 2004). This prompted us to compare the in vitro response to that of the well-established in vivo sterile damage and inflammation models CCl_4_ and partial hepatectomy (PHx) (Gao et al. 2008), and to LPS-induced inflammation. Interestingly, Lcn2 was also the most upregulated gene in all three in vivo models of liver damage [Suppl. Table 5, 6 and Campos et al. (2014)]. A ranking analysis demonstrated that besides Lcn2, the same set of top upregulated genes are induced in monolayer-cultured mouse hepatocytes (day 1) and in vivo after CCl_4_-induced liver damage (Fig. 4a; Suppl. Table 7). This was also true for genes induced in human hepatocytes and HBV-infected human liver tissue (Fig. 4b). Furthermore, all top ten upregulated genes play key roles in inflammation or cellular stress (Suppl. Table 8). To analyze in vitro/in vivo similarities in an unbiased manner, a correlation analysis was performed for genes deregulated in liver diseases and after 24 h of hepatocyte culture. The 24-h cultivation period was chosen because this led to the highest number of deregulated genes in *M*_C_ and *S*. For all mouse (CCl_4_, hepatectomy, HCC, LPS and obese/steatosis) and human (HBV, HCC, cirrhosis, NAFLD) conditions, significant correlations were obtained with genes deregulated after 24 h in *M*_C_ (Fig. 4c) and sandwich cultures (Suppl. Fig. 6). In human hepatocytes, cultivation-induced expression changes also correlated with changes observed in human liver diseases as shown for the example of HBV-infected patients (Fig. 4d; Suppl. Fig. 6). While the complete set of data is available in the supplemental section (Suppl. Figs. 6–10), only the example with the highest correlation coefficient is presented in Fig. 4. To study the time course-dependent correlations, odds ratios of coincidentally (in vivo and in vitro) up- or downregulated genes were calculated for all cultivation periods (Fig. 4e, f). In mouse and human systems, odds ratios of in vivo/in vitro coincidence increased by day 1 followed by a plateau and eventually a minor decrease at longer cultivation periods (Fig. 4e, f; Suppl. Figs. 6–10). It should be noted that in vivo altered genes are mostly a consequence of altered expression in hepatocytes and not by genes expressed on infiltrating leukocytes or non-parenchymal cells, because similar results were obtained when hepatocytes were isolated from CCl_4_-exposed livers compared to liver tissue (Fig. 3c black and gray symbols; Suppl. Table 9). In conclusion, expression changes in cultivated primary human and mouse hepatocytes strongly resemble the transcriptional state of inflamed/disease liver tissue.

### Identification of transcriptional control mechanisms in gene networks activated in cultivated hepatocytes

**Fig. 5:**
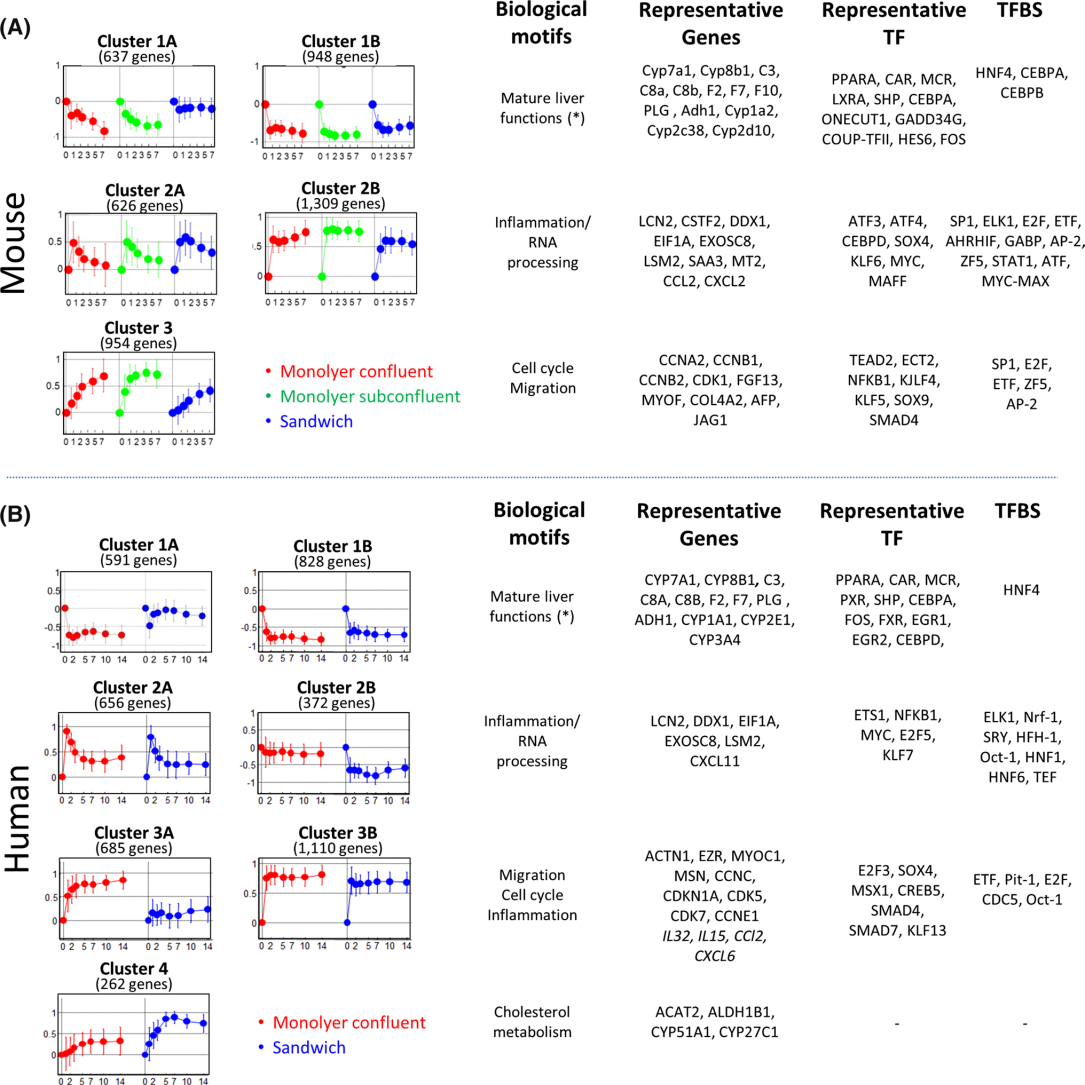
Molecular anatomy of gene expression patterns of cultured hepatocytes reveals biological motifs and their control mechanisms. Time-dependent gene expression profiles in cultivated mouse (**a**) and human (**b**) hepatocytes were established by fuzzy c-means clustering. The *graphs* represent the mean scaled value of all genes contained in each cluster at each time point. The gene clusters were assigned to the biological motifs ‘mature liver functions,’ ‘inflammation and RNA processing,’ ‘cell cycle/migration’ and ‘cholesterol metabolism’ based on enrichment analysis of GO and KEGG terms and by comparison with well-known liver inflammation models in vivo. Representative deregulated genes are shown for each cluster. In addition, genes corresponding to transcription factors (TF) whose expression is deregulated are shown in a separate column. The most significantly overrepresented transcription factor binding sites (TFBS), determined by the PRIMA algorithm (in a region spanning −3000 and +500 nucleotides from the transcription start site), are shown for each gene cluster group. The *numbers* below the *x*-axes represent cultivation period in days

Having described the most obvious gene expression alterations of cultivated hepatocytes, we focused on unveiling the underlying transcriptional control mechanisms. In a first step, we identified groups of tightly correlated genes by time-dependent fuzzy clustering. For mouse hepatocytes, this technique identified five main clusters of which 1A and 1B as well as 2A and 2B were closely related (Fig. 5; Suppl. Table 10). Cluster 1 contains genes involved in mature liver functions (Fig. 5a). The most important difference between Clusters 1A and 1B is that the sandwich culture stabilizes expression of Cluster 1A but not Cluster 1B genes (Fig. 5a). In Cluster 1A, metabolism-associated GO groups and KEGG pathways are overrepresented (Suppl. Table 11). KEGG and GO analyses identify cytochrome P450, sterol metabolism as well as coagulation and complement factors among these ‘sandwich culture maintained’ genes (Suppl. Table 11). This corresponds to the higher sensitivity of sandwich cultures to hepatotoxic compounds that require P450 metabolism such as APAP (Suppl. Fig. 11). Cluster 1B with genes that could not be maintained in sandwich culture differs from Cluster 1A by an overrepresentation of amino acid metabolism (e.g., valine, leucine and isoleucine degradation, *p* = 2.7 × 10^−13^ in Cluster 1B; n.s. in Cluster 1A) as well as peroxisome pathway-associated genes (19 genes, *p* = 1.47 × 10^−07^). The most significantly overrepresented transcription factor binding site of all Cluster 1 genes is HNF4α. Consistently, the expression of HNF4α (and HNF1) was markedly downregulated during the first hours in culture (Suppl. Fig. 12). Likewise, HNF4α nuclear expression was reduced in cultivated cells as compared to fresh hepatocytes (FH) (Suppl. Fig. 12). Clusters 2A and 2B contain the ‘top 10 upregulated inflammation genes’ identified in the previous paragraph (Fig. 5a; Suppl. Table 11). The transcription factor Klf6 was among the strongest transcriptionally upregulated genes of this cluster. Klf6 is a binding partner of SP1 (Botella et al. 2009), which corresponds to the most significantly overrepresented TFBS in this cluster (Fig. 5a). Cluster 2A contains transiently (11 of the 15 top inflammation genes in Fig. 5a), and Cluster 2B permanently upregulated genes (4 of the top 15 genes in Fig. 5a). A well-known feature of liver inflammation is the downregulation of Cebp/α and a concomitant upregulation of Cebp/β and Cebp/δ (Alam et al. 1992). This pattern was also observed in cultivated hepatocytes (Suppl. Fig. 13). Moreover, ELK1, ETF and STAT1 transcription factor binding sites are significantly overrepresented in the inflammation Cluster 2B but not in 2A (Suppl. Table 11). Several independent qRT-PCR experiments with hepatocytes from different mice were performed to confirm the cluster principles of metabolism and inflammation derived from gene array analyses (Supp. Fig. 14). In Cluster 3, cell cycle-associated GO groups (*p* = 5.96 × 10^−15^) and the KEGG pathway ‘DNA replication’ (*p* = 6.6 × 10^−6^) were overrepresented (Suppl. Table 11). Among the most significantly overrepresented TFBS in this cluster were SP1, E2F and ETF (Fig. 5a; Suppl. Table 11). Sandwich cultures reduced upregulation of Cluster 3 genes (Fig. 5a). This corresponds to the lower proliferation activity of sandwich compared to monolayer cultures as evidenced by BrdU incorporation and PCNA expression (Suppl. Fig. 15).

The same approach was used for human hepatocytes leading to four cluster groups (Fig. 5b). Cluster 1 represents loss of mature liver functions (i.e., metabolism) and therefore corresponds to the respective mouse cluster. Similarly, as for mouse the sandwich culture ameliorates the decrease in genes in Cluster 1A (Suppl. Table 12). The strongest induced genes in mouse Cluster 2 represent canonical inflammation genes (e.g., lipocalin 2, Fig. 5a). In the human Cluster 2 these genes were only slightly or not induced (Suppl. Table 12). The substantially milder induction of inflammation genes in human hepatocytes represents a major interspecies difference. Moreover, RNA processing and ribosomal genes are overrepresented in human Cluster 2 (Fig. 5b; Suppl. Table 13). These motifs are also found in the corresponding mouse Cluster 2 (Suppl. Table 11). Cluster 3 contains overrepresented proliferation and migration GO groups, similarly as the corresponding mouse cluster (Fig. 5b). Cluster 4 comprises genes involved in cholesterol metabolism which were upregulated in culture (Suppl. Table 13). The induction of Cluster 4 genes is human specific, since the corresponding mouse genes remain unaltered or are even downregulated. Key transcription factors overrepresented in Clusters 1, 2 and 3 are HNF4, ELK1 and E2F, respectively (Fig. 5b; Suppl. Table 13). Comparison of transcriptional control factors in human and mouse hepatocytes identified similarities (i.e., HNF4 in Cluster 1, ELK1 in Cluster 2 and ETF in Cluster 3) but also major differences, in particular Klf6 and Cebpd which are upregulated in mouse but not in human hepatocytes (Fig. 5a, b).

**Fig. 6:**
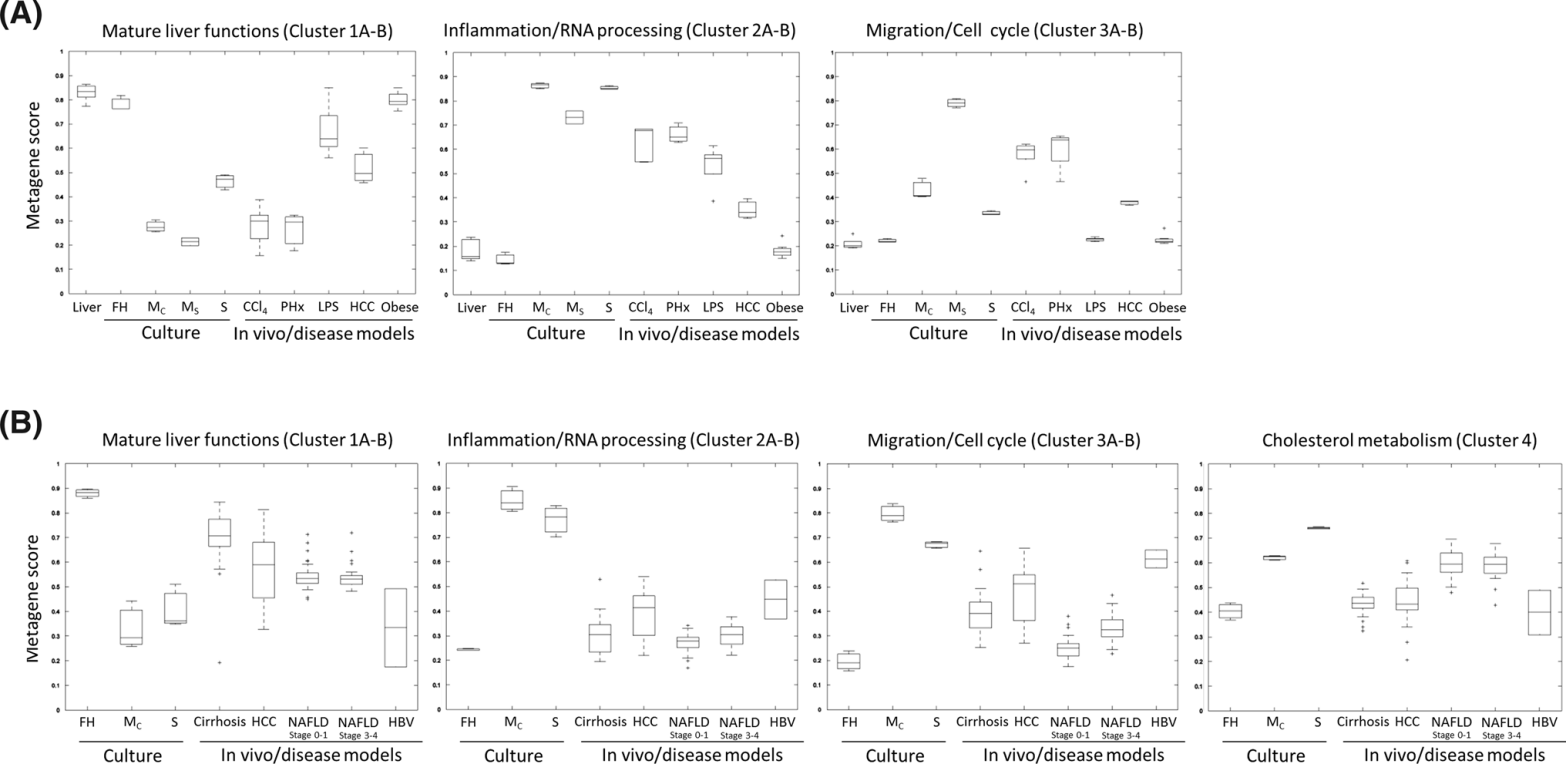
Quantitative analysis of the metagenes calculated for the three main clusters ‘mature liver functions,’ ‘inflammation/RNA processing’ and ‘migration/cell cycle’ in cultured hepatocytes and liver disease models in mouse (**a**) and human (**b**). Genes assigned to the metagenes ‘mature liver functions,’ ‘inflammation/RNA processing’ and ‘migration/cell cycle’ (and ‘cholesterol metabolism’ for human) were determined by selecting the top deregulated genes from each Fuzzy cluster group (Fig. 5; see ‘metagene analysis’ in ‘supplemental materials and methods’). The graphs indicate the metagene score for each condition, which corresponds to the mean scaled value of all genes of the corresponding cluster groups as identified in Fig. 5. For mouse (**a**), the analysis was performed based on expression levels in hepatocytes at day 1 in *M*_C_, *M*_S_ and *S*, and the in vivo models of CCl_4_ intoxication at day 1 (CCl_4_), partial hepatectomy at day 1 (PHx), LPS-induced inflammation (LPS), hepatocellular carcinoma (HCC) and fatty liver (obese). For human (**b**), the analysis includes gene expression levels at day 1 in cultured hepatocytes, and on the in vivo disease models of cirrhosis, HCC, non-alcoholic fatty liver disease (NAFLD) on stages 0–1 and 2–4, and in HBV-infected liver tissue

Based on the aforementioned clusters, metagenes can be calculated which represent a normalized mean of the genes in the respective cluster (Schmidt et al. 2008). Metagenes allow systematic comparisons between samples in vitro and ex vivo, and confirmed that similar changes were induced by the isolation and cultivation stress as in some disease conditions (metagene list in Suppl. Table 14). In mouse, Cluster 1 metagene corresponding to mature liver functions was downregulated in cultivated hepatocytes and also after CCl_4_, partial hepatectomy and to a minor degree in LPS treated mice and in HCC, but not in obese/steatotic mice (Fig. 6a). The inflammation Cluster 2 shows a reciprocal pattern to that of Cluster 1 (Fig. 6a). The cell cycle/migration Cluster 3 shows major differences between the culture systems with stronger upregulations in subconfluent monolayers and an ameliorated response in sandwich cultures (Fig. 6a). In vivo, Cluster 3 responses are strongest after CCl_4_, partial hepatectomy and in HCC, whereas obese/steatotic and LPS treated mice showed no response (Fig. 6a). Similar responses were observed in human hepatocytes (Fig. 6b). For diseased human livers, the strongest responses of Clusters 1–3 were seen for HBV, while deregulation of metagenes was comparably mild for cirrhosis, NAFLD and HCC (Fig. 6b). In contrast, Cluster 4 response (cholesterol metabolism) was stronger in NAFLD compared to cirrhosis, HCC and HBV. The results of the metagene approach are remarkable, because they illustrate the similarity of strong and systematic expression alterations of highly correlated genes in vitro and in vivo and allow the quantitative comparisons of different liver diseases.

### Interspecies comparison identifies predominantly similar responses in human and mice

**Fig. 7:**
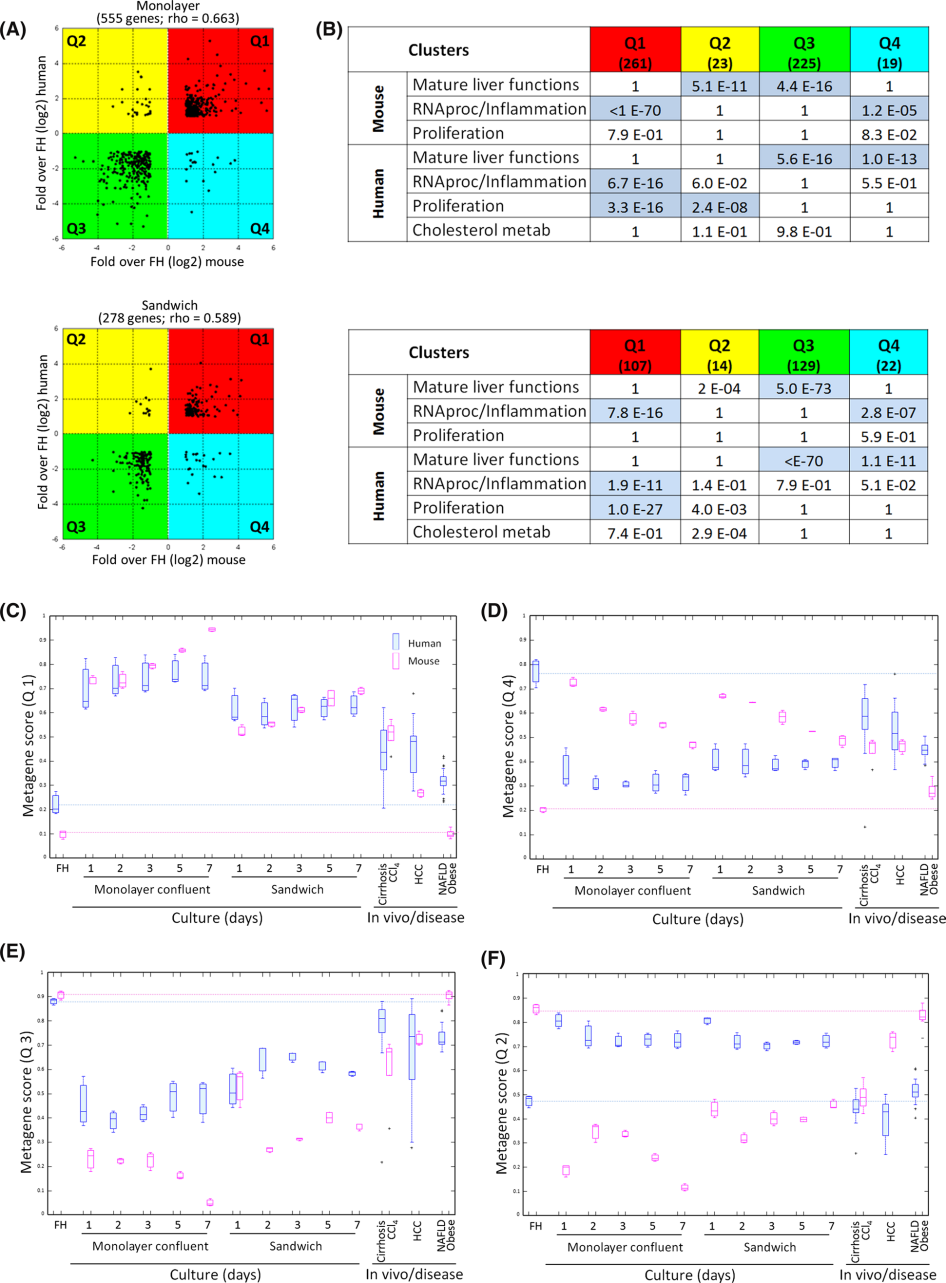
Similarity of transcriptional alterations in cultivated mouse and human hepatocytes. **a** Scatter plot analysis of differentially expressed genes at day 1 in monolayer or sandwich culture. The *graph* shows only genes whose expression levels are at least twofold deregulated (at cultivation day 1). Quadrants are defined as either upregulated in both mouse and human (*Q1*), downregulated in mouse and human (*Q3*) or inversely deregulated in mouse and human (*Q2* and *Q3*). **b** Overrepresentation of genes representing the biological motifs (described in Fig. 5) for each correlation quadrant. The table shows the *p* value for each biological motif in the respective quadrant (gray boxes shown *p* < 0.05). **c**–**f** Metagene analysis of deregulated genes in mouse and human hepatocytes and tissue of diseased livers. The analysis was performed using the top ten deregulated genes in cultured hepatocytes (orthologues in mouse and human), for each independent quadrant (as shown in **a**). The *bars* indicate the mean scaled value of all genes for each metagene. *Blue and red boxes* correspond to human and mouse metagenes, respectively. *Dashed lines* indicate the reference expression levels in freshly isolated hepatocytes

Interspecies comparison of diseases in human and laboratory animals are often difficult, because the disease stimuli may differ. However, the isolation and cultivation stress of hepatocytes is similar for hepatocytes of both species and therefore offers excellent conditions for interspecies comparison. For a systematic analysis, corresponding (orthologous) mouse–human gene pairs were identified (Suppl. Table 15) (Yue et al. 2014). Scatter plots with cultivation-induced gene expression alterations of mouse and human genes were established (Fig. 7a). For monolayer as well as sandwich cultures, significant correlations were obtained, with coefficients of 0.663 and 0.589, respectively (Fig. 7a). This analysis identifies genes with similar responses in both species (Q1 and Q3) and genes with opposite responses (Q2 and Q4). To understand which biological motifs are represented in Q1–Q4, we assessed their overlap with the cluster groups identified in Fig. 5. Q1 contains genes upregulated in both human and mouse and is dominated by genes of the inflammation/RNA processing and proliferation/migration clusters (Fig. 7b). Q3 with downregulated genes in both human and mouse represents the mature liver functions/metabolism cluster (Fig. 7b). The quadrants Q2 and Q4 contain genes with opposite expression changes in human and mouse hepatocytes, thus representing interspecies differences (Fig. 7b). Q2 contains downregulated genes in mouse hepatocytes representing mature liver functions, and upregulated genes in human hepatocytes representing the proliferation cluster. Statistical significance for each motif was observed only in deregulated genes in monolayer cultures (Fig. 7b). Conversely, Q4 contains upregulated genes in mouse hepatocytes representing RNA processing/inflammation motif, and downregulated genes in human hepatocytes representing mature liver function cluster (Fig. 7b). Using a ranking approach, top diagnostic genes showing similar responses in human and mouse were identified (Suppl. Table 16). This technique allows a quantitative and unbiased interspecies comparison, because identical genes are compared between both species and the rules of identifying these genes were identical in human and mouse. This approach demonstrates that genes in Q1 (inflammation and proliferation associated) show quantitatively similar responses in both species not only in vitro but also for the studied disease conditions (Fig. 7c). In contrast, Q4 refers to inflammation-associated genes, but they are upregulated in mice which are down regulated in human (Fig. 7d). These responses are stronger in cultivated hepatocytes than in liver disease. In Q3 similar responses in form of downregulation in vitro and in vivo are seen for human and mouse, whereby mice show stronger responses (Fig. 7e). In contrast Q2 shows opposite responses of mature liver function genes, with downregulations observed in mouse hepatocytes and liver disease, whereas in human hepatocytes these genes were upregulated or unchanged (Fig. 7f). In conclusion, a systematic interspecies comparison reveals that the majority of genes (Q1 + Q3 = 486 genes in *M*_C_) show similar responses, whereas a minority (Q2 + Q4 = 42 genes in *M*_C_) responds differently.

### Interventions improving hepatocyte differentiation

**Fig. 8:**
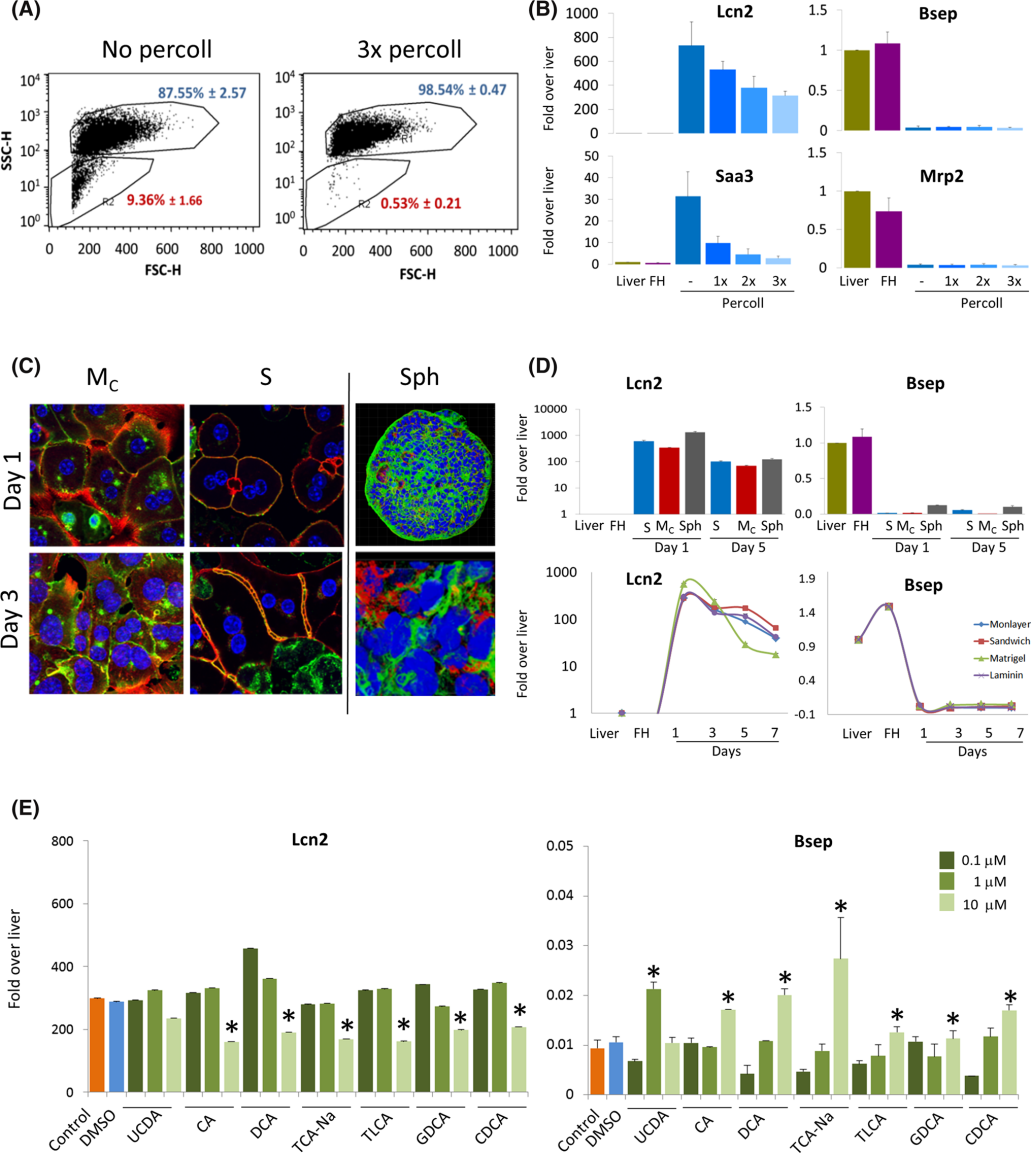
Interventions to reduce cultivation-dependent transcriptional alterations in primary hepatocytes. **a** FACS analysis of cell suspensions from perfused mouse liver before and after three purifications with Percoll gradients. The *scatter plots* indicate size (FSC-H) and granularity (SSC-H). The populations representing hepatocytes and non-parenchymal cells (NPCs) plus debris are *marked*, and their corresponding ratios are shown in *blue* for hepatocytes and *red* for NPCs. The fraction of NPCs is drastically reduced after successive purifications with Percoll. **b** Real-time qPCR analysis of the diagnostic genes for ‘inflammation’ (Lcn2, Saa3) and ‘mature liver functions’ (Bsep, Mrp2) in hepatocytes on monolayer culture (day 1), without or after 1, 2 or 3 purifications with Percoll gradient centrifugation. **c** Confocal images of mouse hepatocytes stained with phalloidin (*red*), anti-DPPIV antibodies (*green*) and DAPI (*blue*), showing polymerized actin, apical domains and nuclei, respectively. The analysis was performed on hepatocytes in monolayer confluent (*M*_C_), sandwich (*S*) and spheroid cultures. The spheroid was stained at day 5 in culture. **d** Real-time qPCR analysis of the diagnostic genes for the ‘inflammation’ (Lcn2) and ‘mature liver functions’ (Bsep) in hepatocytes cultivated on different matrix supports. The *top graphs* show the expression levels of diagnostic genes in sandwich (*S*), monolayer confluent (*M*_C_) and spheroids (Sph) at days 1 and 5. The *bottom graphs* show expression levels of diagnostic genes in hepatocytes cultured on collagen coated monolayer confluent (monolayer), collagen sandwich (sandwich), matrigel and laminin at the indicated cultivation periods. **e** Real-time qPCR analysis of the diagnostic genes for ‘inflammation’ (Lcn2) and ‘mature liver functions’ (Bsep), in hepatocytes cultures on collagen monolayer using standard culture media (control) or additional bile salts for 24 h. DMSO was included as vehicle control. *UCDA* ursodeoxycholic acid, *CA* cholic acid, *DCA* deoxycholic acid, *TCA-Na* taurocholic acid, *TLCA* taurolithocholic acid, *GDCA* glycodeoxycholic acid, *CDCA* chenodeoxycholic acid (*p < 0.05 compared to controls, Mann–Whitney test, two-sided)

The biostatistical analyses described in the previous chapters deliver a blueprint for interventions to improve hepatocyte differentiation. One potential explanation for the strong ‘sterile’ inflammatory response in cultivated hepatocytes could be impurities due to NPCs, e.g., stellate cells, Kupffer cells or sinusoidal endothelial cells, that might be activated in culture to secrete cytokines and cause the inflammatory transcriptional response in neighboring hepatocytes. To test this hypothesis we first characterized the NPC fraction from freshly isolated mouse hepatocytes by FACS (Fig. 8a) and manual counting of H&E stained cell suspensions (Suppl. Fig. 16). Both techniques indicated a relatively high fraction of approximately 10–13 % NPCs which could be almost completely eliminated by Percoll centrifugation (Fig. 8a). Despite a small residual fraction of approximately 1 % NPCs that could not be removed by further purifications steps, the difference in unpurified cells offers adequate conditions to study a possible influence of the NPC fraction. Removal of NPCs reduced the inflammation response as evidenced by the diagnostic cluster genes Lcn2 and Saa3 (Fig. 8b), whereas diagnostic genes of the metabolism cluster Bsep and Mrp2 were not rescued by NPC removal (Fig. 8b).

A more generic approach to establish a microenvironment that better reflects the in vivo situation is offered by spheroids where hepatocytes form their own matrix (Landry et al. 1985), in contrast to collagen sandwich cultures where hepatocytes are kept between two layers of soft gel made of collagen type I isolated from rat tail (Uygun et al. 2010). In both cultivation systems, sandwich and spheroids, hepatocytes established a polar phenotype as evidenced by DPPIV staining of bile canaliculi (Fig. 8c). Analysis of representative genes from Cluster 1 (mature liver functions) and Cluster 2 (inflammation/RNA processing) showed qualitatively similar alterations in both culture systems (Fig. 8d). However, the degree of downregulation of some metabolism cluster genes (e.g., Bsep) is slightly ameliorated in spheroids as compared to sandwiches, which may prioritize the latter system for hepatocyte physiology (Fig. 8d). Consistently, expression of HNF4α, whose binding site is overrepresented in the metabolism cluster genes, was better maintained in spheroids than in sandwich cultures (Suppl. Fig. 17). However Cebpδ, a transcription factor responsible for expression of inflammation genes, is higher in spheroids, illustrating that this culture type does not suppress the inflammation response (Suppl. Fig. 17). Comparison of collagen sandwich cultures to other types of commercially available matrixes, such as matrigel and laminin demonstrated similar alterations of metabolism and inflammation gene clusters (Fig. 8d; Suppl. Fig. 18), indicated that the systemic alterations in gene expression occur independent from the specific type of extracellular matrix.

One of the results of the fuzzy clustering analysis was the identification of downregulated mature liver functions (Fig. 5) which includes a cluster of genes involved in bile salt metabolism. Since routinely used hepatocyte culture media do not include bile salts (Godoy et al. 2013), but on the other hand bile salts have been reported to induce mature liver functions (Avior et al. 2015) (Sawitza et al. 2015), we tested their influence in the present experimental system. Of seven bile salts, six significantly suppressed the diagnostic inflammation cluster markers Lcn2 (Fig. 8e) and Saa3, while Mt2 remained unaltered (Suppl. Fig. 19). Similarly, significant increases in the diagnostic metabolism cluster markers Bsep (Fig. 8e), Mrp2 and Cyp7a1 (Suppl. Fig. 19) were obtained.

One of the candidates identified by GRN analysis that potentially contributes to orchestrating the transcriptional inflammation response is Klf6 (Fig. 5). Klf6 is a binding partner of the overrepresented TF SP1 (Fig. 5), and it was transcriptionally upregulated in cultivated hepatocytes and in mouse liver disease (Fig. 5; Suppl. Table 1–3, 6). The biostatistical results were confirmed by qRT-PCR (Suppl. Fig. 20), immunostaining showed nuclear translocation of Klf6 (Suppl. Fig. 20) and western blot demonstrating upregulation of Klf6 in cultivated hepatocytes and in vivo after CCl_4_ administration (Suppl. Fig. 20). However, siRNA-mediated knockdown of Klf6 in cultivated mouse hepatocytes led only to a minor increase in Bsep expression (Suppl. Fig. 20). A second knockdown approach was performed for MafF, another transcription factor involved in the inflammation response. A pool of 4 oligos against MafF reduced expression in cultivated mouse hepatocyte by approximately 90 % (Suppl. Fig. 20). This led to a statistically significant reduction in the marker genes of the inflammation cluster Lcn2, Mt2, and Saa3, and increased the expression of metabolism cluster genes Bsep, Mrp2 and Cyp7a1 (Suppl. Fig. 20). However, it should be considered that the size of this recue effect is small and by far does not restore the expression levels to those of freshly isolated primary hepatocytes.

Further interventions to ameliorate the massive expression alterations in cultivated hepatocytes can be undertaken at the level of the signaling network. LUMINEX analysis of phosphoproteins in freshly isolated mouse hepatocytes and after cultivation for up to 7 days showed a strong increase in p-ERK1/2, p-JNK, p-MEK1, p-p38, and p-p70S6 compared to healthy mouse liver tissue (Suppl. Fig. 21). Consistently, we observed genes of the MAPK pathway upregulated in all cultures, including Map3k5, Map3k6, Mapk3, VRas and Rras (Suppl. Table 10, Cluster 2B). Perfusion of mouse livers with EGTA and collagenase either alone or in combination shows that EGTA alone is sufficient to induce the identified signaling activity (Suppl. Fig. 21). It is known since long that isolated hepatocytes can be kept in cold storage for more than 24 h without compromising their plating efficacy (Hengstler et al. 2000). Cold storage for 24 h reduced the phosphorylation levels of all aforementioned signaling proteins; however, this did not blunt the expression responses of representative genes of Clusters 1–3 (Suppl. Fig. 22). Therefore, ‘cooling down’ the signaling activities of freshly isolated ‘burning’ hepatocytes before plating does not prevent the massive isolation and cultivation-induced expression changes shown in Fig. 1.

## Discussion

In a genome-wide, time-resolved profiling study initially designed to improve human and mouse hepatocyte in vitro systems we made the surprizing observation that expression changes induced by hepatocyte isolation and cultivation resemble expression alterations in liver diseases. We demonstrate by genome-wide analysis that gene cluster responses in vitro, i.e., upregulation of inflammation/RNA processing and migration/cell cycle genes, downregulation of mature liver functions genes, also occurred in human HBV-infected liver tissue, cirrhosis and HCC, as well as CCl_4_ intoxication, partial hepatectomy, LPS intoxication and HCC in mouse, although the intensity varied and was generally weaker in diseased liver than in vitro. Our observations are in agreement with previous reports that also identified components of the acute phase response among the top upregulated genes in cultivated hepatocytes (Boess et al. 2003). However, our study goes beyond those observations because we establish precise correlations to disease models in vivo, and unravel control mechanisms responsible for the altered gene expression in vitro, allowing the design of molecular interventions. Gene network analysis identified a low HNF4 signature as one of the major reasons for loss of mature liver functions. Gain of inflammation/RNA processing was mostly driven by Klf6 and interaction partners, Cebp and ATF stress factors. Central control factors for the migration/cell cycle cluster include E2F family members.

Possible reasons for gene expression alterations in cultured primary hepatocytes are contamination with non-parenchymal cells, unphysiological extracellular matrix (ECM) or lacking soluble environmental cues present in normal liver such as bile salts. In our study, we addressed all these aspects individually. First, a strong reduction of NPCs contamination by Percoll gradient resulted in partial suppression of genes from the inflammation/RNA processing cluster. Second, different types of ECM were tested. We have previously observed that recombinant extracellular matrix proteins (e.g., laminins) significantly improve hepatocyte differentiation of HLCs compared to cancer-derived matrix (e.g., matrigel) (Cameron et al. 2015). In primary hepatocytes, however, matrigel or laminin ECM did not provide significant improvements in expression of either ‘inflammation’ or ‘mature liver function’ genes compared to collagen I cultures. Interestingly in hepatocyte spheroids, where cultivated hepatocytes generate their own matrix, a significant increase in genes from the ‘mature liver functions’ cluster was seen. Nonetheless, this increase is small when comparing to expression levels of healthy liver. Addition of bile salts increased expression of mature liver function genes (e.g., Bsep) although the achieved levels were again far below those of healthy liver. Interestingly, all tested bile salts decreased inflammation cluster genes. Knockdown of Klf6 and MafF, central transcription factors of the inflammation/RNA processing cluster, led only to a small decrease in inflammation cluster genes, suggesting that manipulation of only single transcription factors is not sufficient to tip the balance from inflamed livers back to the normal situation. Taken together, this illustrates that several mechanisms contribute to the altered cell state; hence, none of the individual interventions can result in a full rescue. However, the results strongly suggest that Percoll gradient purification and bile acid supplementation should be done routinely. Also, further factors of influence such as LPS contamination of collagenase or the strong induction of several signaling pathways in response to the isolation stress (‘burning hepatocyte phenomenon’) were individually not sufficient to explain the altered state of cultivated hepatocytes. In conclusion, the transcriptional state of hepatocytes in culture represents a multifactorial phenomenon that closely resembles gene expression patterns and transcriptional regulatory networks in liver disease. For pharmacological and toxicological routine work, this means that we should be aware that the frequently applied cultivated hepatocyte systems represent an inflamed liver. Importantly, this represents a disease model that can be exploited to develop anti-inflammatory strategies for liver disease. It is of interest that cultivated stellate cells, even when isolated from healthy livers, have been widely used as a model of liver fibrosis (Friedman 2008). Similarly, cultivated primary hepatocytes represent an inflammatory model in vitro.

A question of high practical relevance is mouse-to-human extrapolation, since most therapeutic concepts are initially tested in rodents. However, the relevance of mouse inflammation data for humans has been challenged up to the extreme position that there are no relevant similarities at all (Leist and Hartung 2013). The present study using mouse and human hepatocytes under identical pro-inflammatory culture conditions offers a blueprint for rational interspecies extrapolation. Qualitatively similar is the inflammation/RNA processing response that in both species is driven by increased Krüppel-like factors, Sox4, Myc, Tead2 (ETF) and ELK1. It should be considered that despite of this overall correlation, important differences can be identified, such as the key inflammation-associated transcription factor Cebpd which is upregulated in mouse, but repressed in human primary hepatocyte cultures. A further common feature of both species is downregulation of mature liver function genes such as Pck1, Adh4 and G6pc that are more than tenfold downregulated in both species, with HNF4 as a dominant control factor. In conclusion, the knowledge of interspecies conserved versus distinct transcriptional networks allows predicting which features observed in mice can or cannot be extrapolated to humans.

The present study leads to the following practical recommendations: (1) Sandwich cultures or spheroids provide the most effective culture conditions to sustain expression of genes associated with mature liver functions. However, when an in vivo-like metabolism is critical, for instance in the study of metabolism-associated toxicity, compound exposures should be performed within the first 24 h of cultivation, due the time-dependent suppression of mature liver function genes. This is even more critical for mouse than human hepatocytes. (2) Collagen monolayers provide a system where proliferation-associated genes can be induced. This feature is also more substantial in mouse hepatocytes which can engage in cell proliferation. (3) When primary hepatocytes are cultivated over longer periods, they approach transcriptional states of hepatocyte cell lines and stem cell-derived hepatocyte-like cells. In conclusion, the present genome-wide study of primary human and mouse hepatocytes demonstrates that the broadly applied culture systems represent models of inflamed livers.

## Electronic supplementary material

Below is the link to the electronic supplementary material.

Supplementary material 1 (DOCX 542 kb)

Supplementary material 2 (XLSX 19 kb)

Supplementary material 3 (XLS 5220 kb)

Supplementary material 4 (XLS 6066 kb)

Supplementary material 5 (XLS 6031 kb)

Supplementary material 6 (XLS 2799 kb)

Supplementary material 7 (XLS 8479 kb)

Supplementary material 8 (XLS 14707 kb)

Supplementary material 9 (XLS 36 kb)

Supplementary material 10 (XLS 3503 kb)

Supplementary material 11 (XLS 4149 kb)

Supplementary material 12 (XLS 6828 kb)

Supplementary material 13 (XLS 1399 kb)

Supplementary material 14 (XLS 6259 kb)

Supplementary material 15 (XLS 60 kb)

Supplementary material 16 (XLS 170 kb)

Supplementary material 17 (XLSX 89 kb)

Supplementary material 18 (XLSX 12 kb)

Supplementary material 19 (XLSX 11 kb)

Supplementary material 20 (DOCX 25 kb)

Supplementary material 21 (DOCX 12128 kb)

## Abbreviations

FH: Freshly isolated hepatocytes
NPCs: Non-parenchymal cells
*M*_C_: Monolayer confluent culture
*M*_S_: Monolayer subconfluent culture
*S*: Sandwich culture
PHx: Partial hepatectomy
LPS: Lipopolysaccharide
EMT: Epithelial to mesenchymal transition
PC: Principal component
