# Supplementary material for: Gene network activity in cultivated primary hepatocytes is highly similar to diseased mammalian liver tissue
Source: Arch Toxicol. 2016 Jun 23;90(10):2513–29. doi: 10.1007/s00204-016-1761-4 (PMC5043005; doi:10.1007/s00204-016-1761-4)
Supplement: Supplementary file 1 — Supplementary material 1 (DOCX 542 kb) [file 204_2016_1761_MOESM1_ESM.docx]

**Supplemental materials and methods for the manuscript “Gene network activity in cultivated primary hepatocytes is highly similar to diseased mammalian liver tissue”**

Patricio Godoy^1,17,*,**^, Agata Widera^1,*^, Wolfgang Schmidt-Heck^2,*^, Gisela Campos^1^, Christoph Meyer^3^, Cristina Cadenas^1^, Raymond Reif^1^, Regina Stöber^1^, Seddik Hammad^1,3,16^, Larissa Pütter^1^, Kathrin Gianmoena^1^, Rosemarie Marchan^1^, Ahmed Ghallab^1,16^, Karolina Edlund^1^, Andreas Nüssler^4^, Wolfgang E. Thasler^5^, Georg Damm^6^, Daniel Seehofer^6^, Thomas S. Weiss^7^, Olaf Dirsch^8^, Uta Dahmen^9^, Rolf Gebhardt^10^, Umesh Chaudhari^11^, Kesavan Meganathan^11,18^, Agapios Sachinidis^11^, Jens Kelm^12^, Ute Hofmann^13^, René P. Zahedi^14^, Reinhard Guthke^2^, Nils Blüthgen^15^, Steven Dooley^3^, Jan G. Hengstler^1**^

**Reagents**

A complete list of antibodies ant TaqMan assays is provided in the supplemental tables 17 and 18, respectively.

**Primary hepatocyte isolation and culture (mouse and human)**

Primary mouse hepatocytes were obtained by a two-step perfusion technique from male Black6N mice (8-12 week old) as previously described (1). Primary human hepatocytes were obtained from liver sections from patients undergoing surgical liver resection under patient informed consent (following the 1975 Declaration of Helsinki) as previously described (1). The Ethics approval documents are provided as supplemental information. The percentage of viable cells was determined by trypan blue exclusion. The minimal viability of hepatocytes used in this study was 97%. Detailed protocols for the isolation of mouse, rat and human hepatocytes are described in Godoy 2013 (1). For conventional culture configurations (monolayer confluent-M_C_ or monolayer subconfluent-M_S_) hepatocytes were plated onto collagen-I coated dishes at a density of 104166 cells/cm^2^ or 13000 cells/cm^2^, respectively. For collagen sandwich cultures (S), hepatocytes were plated at 88542 cells/cm^2^. Detailed protocols for the cultivation of hepatocytes are described in Godoy et al 2013 (1). For Matrigel and Laminin cultures, hepatocytes were plated in Matrigel® (BD Biosciences, Heidelberg, Germany) or Laminin (BD Biosciences, Heidelberg, Germany) coated dishes following the manufacturer’s instructions. Hepatocyte morphology was daily assessed with a phase contrast microscope (Nikon, Düsseldorf, Germany), equipped with a Digital Sight DS 2MBW digital camera.

**In vivo models of liver damage, inflammation and regeneration**

All experiments were performed in male C57Black6/N mice (8-12 weeks old, Charles Rivers, Sulzfeld, Germany), except C57BL6.V-Lepob/JRj obese mice (carrying a deletion of leptin receptor) which were obtained from Janvier (Le Genest-Saint-Isle, France). Wild type C57Black6/N mice were fed ad libitum with Ssniff R/M-H, 10 mm standard diet (Ssniff, Soest, Germany). All experiments were approved by the local Ethic committees. 4-5 mice were used for each time point in each model. For CCl_4_-induced acute liver damage, mice received a single intraperitoneal injection of CCl_4_ (1,6 g/kg body weight) dissolved in olive oil as previously described (2). For lipopolysaccharide (LPS) model, mice received a single intraperitoneal injection of LPS (750 ng/kg) dissolved in sterile PBS. At the indicated time points, mice were anesthetized and the liver was resected, washed in ice cold PBS and sectioned for further analysis. Sections of about 0.5 cm^2^ were snap frozen in liquid nitrogen and stored at -80°C for subsequent isolation of proteins or RNA.

A precise vessel-oriented, parenchyma-preserving surgical technique was used for 70% partial hepatectomy, using a modification of the technique described by Madrahimov (3) for rats. All surgical interventions were performed under inhalation of 2% isoﬂurane mixed with an oxygen flow of 0.3 L/min (isoflurane vaporizer, Sigma Delta, UK) in S1 operation room. The procedure started with a laparotomy via a transverse abdominal incision. The whole liver was well exposed by elevating the xiphoid process. Skin and muscle were fixed by retractors, and the small bowel was moved out to the left side of the abdomen and covered with saline soaked gauze. The liver was freed from the falciform ligament and triangular ligamentum. The liver lobes were positioned so that hilum of left lateral lobe (LLL) and median lobe (ML) were clearly visible. All subsequent steps were performed using a stereo microscope with a 10x magnification. A ligature (6-0 silk) was applied loosely to the pedicle of LLL. The ligature was tightened keeping a distance of about 3 mm from the cava while the LLL remained in its anatomical position and the lobe resected. Next, cholecystectomy was performed after double ligating the cystic duct and cystic artery using a 7-0 prolene suture. For resection of the median lobe, a virtual line was drawn between the left side of the cava and the gallbladder. The clamp was placed, roughly perpendicular to the surface of the left median lobe (LML), about 3mm lateral to this line and the left median lobe removed. Proximal to the clamp, a piercing suture was positioned according to vascular anatomy to ligate the left median hepatic vein and the clamp removed. Then, the right median lobe (RML) was clamped in similar way. After resection, two piercing sutures were placed to ligate the right and median hepatic vein as well as the arterial and portal supply. Finally, the abdomen was irrigated with warm saline solution and closed with a 2 layer running suture (6-0 prolene). At the end of anaesthesia, animals were allowed to recover on a heating pad. Temgesic (0.05 mg/kg) was applied subcutaneously after operation and at an interval of 12 hours in next 3 days. Animals were monitored daily for body weight development and activity using a scoring system (6). Briefly, mice with normal activity, physiological position, no jaundice, and no signs of bleeding were regarded as healthy (+++); animals showing a weaker activity, hunched back position and/or signs of jaundice or bleeding were regarded as weak (++); and animals with no spontaneous activity and lying position and signs of jaundice or bleeding were regarded as severely ill (+). Same as for the CCl_4_ and LPS conditions, mice were anesthetized at the indicated time points and the liver was resected, washed in ice cold PBS and sectioned for further analysis. Sections of about 0.5 cm^2^ were snap frozen in liquid nitrogen and stored at -80°C for subsequent isolation of proteins or RNA.

**RNA isolation**

RNA was isolated from cultured hepatocytes and from mouse liver tissue using the Phenol/Chloroform method (Trizol®, Qiagen, Hilden, Germany) according to the manufacturer’s description. For freshly isolated hepatocytes, 1 million cells in suspension medium were allowed to decant in a 1.5 ml conical tube placed on ice for 10 minutes. The medium was quickly removed and the cells homogenized in 1 ml of Trizol® (Qiagen, Hilden, Germany) by 10 repetitive aspirations in a micropipette. For hepatocytes in culture, the medium was removed and the cells were homogenized in 1 ml Trizol® (Qiagen, Hilden, Germany) with a micropipette. For liver tissue, snap frozen sections were homogenized in a 1.5 ml tube with 1 ml Trizol® (Qiagen, Hilden, Germany) using a plastic pestle and subsequent sonication on ice. For spheroids (see section “hepatocyte spheroids”), all microtissues were collected from three independent plates by gently flushing the hanging drops into a sterile Petri dish. The spheroids were concentrated by swirling the dishes, collected with a micropipette and transferred to a 1.5 ml tube. After decanting for 10 minutes on ice, the medium was removed and the microtissues were homogenized in 500 µl of Trizol® (Qiagen, Hilden, Germany). RNA concentration and integrity and were determined spectrophotometrically in a Nanodrop®2000 (ThermoScientific, Waltham MA, USA) and in a Bioanalyzer® (Agilent, Waldbronn, Germany) respectively.

**Affymetrix® gene array analysis**

Affymetrix gene array analysis was performed as previously described (4), using the Affymetrix GenChip® Mouse Genome 430 2.0 arrays (Santa Clara, CA, USA). Briefly, five µg RNA were transcribed into cDNA by oligo dT primers, and reverse transcribed to biotinylated cRNA with the Gene Chip IVT® Labeling kit (Affymetrix, High Wycombe, UK). Cleanup of the IVT product was done using CHROMA SPIN-100 columns (Clontech, USA). Spectrophotometric analysis was used for quantification of cRNA with acceptable A260/A280 ratio of 1.9 to 2.1. After that the cRNA was fragmented using Affymetrix’s protocol. Labeled and fragmented cRNA was hybridized to Mouse Genome 430 2.0 Affymetrix GeneChips for 16h at 45° C according to the manufacturer’s instructions. Microarrays were washed using an Affymetrix fluidics station 450 and stained initially with streptavidin-phycoerytherin. For each sample the signal was further enhanced by incubation with biotinylated goat anti-streptavidin followed by a second incubation with streptavidin-phycoerytherin and a second round of intensities were measured. Microarrays were scanned with an Affymetrix scanner controlled by Affymetrix Microarray Suite software.

**Microarray processing and statistical analysis**

Affymetrix gene expression data were pre-processed using ‘affyPLM’ packages (5) of the Bioconductor Software (6). To obtain the genes with the most evidence of differential expression, a linear model fit was applied for each gene using ‘limma’ (Linear Models for Microarray Data) packages (7) of (6). Data obtained from fresh hepatocytes were used as reference. The custom chip definition file from Brainarray (8) based on Unigene ID’s was used to annotate the microarrays. A false positive rate of a=0.05 with FDR correction and a fold change greater 2 was taken as the level of significance. Processing and visualization (Principal Component Analysis) of data were performed using MATLAB tools (The MathWorks Inc., Natick, MA).

**Additional gene array expression profile from mouse and human cell lines and disease tissue**

All additional gene array data was collected from the Gene Expression Omnibus or ArrayExpress databases. We used only datasets generated with Affymetrix gene arrays as follows: acute liver damage in mouse by single intraperitoneal injection of CCl_4_ (E-MTAB-2445) (9), mouse HCC (GSE33446), generated by CCl_4_ and diethylnitrosamine intraperitoneal administration (10); mouse hepatocyte-like cells iHep (GSE59037) generated by conversion of mouse embryonic fibroblast using retrovirus vectors with HNF4a and Foxa1 (11); mouse hepatic cell line AML12 (GSE11703) (12); Human HepG2 and Human HepaRG cells (13); human hepatocyte-like cells (HLC) derived from embryonic or induced-pluripotent stem cells (14); human liver tissue infected with hepatitis B (GSE14668) (15); human liver tissue with non-alcoholic fatty liver disease (NAFLD, score 0-1, 3-4) (GSE49541) (16); human liver cirrhosis and human hepatocellular carcinoma (GSE17548) (17).

**Ranking analysis of deregulated genes in cultivated hepatocytes and in in vivo disease models**

To detect genes with similar responses under different condition a ranking approach is used. The obtained fold-changes under each condition are sorted in decreasing order and the appropriate ranks are associated to the genes. The final rank of genes is obtained by summarizing the ranks of each condition

**Pearson’s rank correlation and odds ratio analysis**

To calculate the strength of the relationship between cultivation (M_C_, M_S_, S) and the in vivo models CCl_4_, PHx, LPS and HCC models in mouse, and HCC, cirrhosis, NAFLD and HBV in human, we used Pearson’s rank correlation and odds ratio analyses. The gene expression level at different time points in cultivation (M_C_, M_S_, S) and in each in vivo model were used for the calculation of the correlation coefficients. The number of differentially expressed genes (DEGs) at different time points in the cultivations series and in each in vivo model and the resulting intersection were used to calculate the strength of association (odds ratio) between the model systems.

**Fuzzy clustering of gene expression profiles**

The time-profiles of the differentially expressed genes in cultivated mouse or human hepatocytes were scaled between their respective absolute temporal extreme values to focus subsequent cluster analysis on the qualitative behavior of the expression profiles. The time series were clustered using fuzzy c-means (18) (fuzzy exponent = 1.5; maximum number of iteration = 200; minimum cost function improvement = 10-10). The optimum number N of cluster was estimated by repeated calculation (number of iterations = 100) of the fuzzy cluster index ‘Separation Index’. Clusters containing genes with similar trends in expression and similarly enriched in gene ontology and KEGG pathway terms were joined into cluster groups.

**Gene Set Enrichment Analysis (GSEA) (overrepresented Gene Ontology (GO) terms, KEGG pathways, and transcription factor binding sites (TFBS))**

1. Gene ontology (GO) and KEGG pathway analysis: Genes which show change ratios greater (or less) than 2-fold in the triplicate arrays at any one of the time points have been considered as up or downregulated and subjected for gene ontology (GO) and pathway analyses. DEGs were categorized using the manually curated Gene Ontology of the Biobase Knowledge Library (BKL) of the ExPlain™ webservice (BioBase GmbH, Wolfenbüttel, Germany) (19). The web based tool g:Profiler ((http://biit.cs.ut.ee/gprofiler/), 15) is used for additional functional interpretation of the list of DEGs related to the pathway database KEGG (20).

# Promotor analysis for overrepresented transcription factor binding sites (TFBS): To identify transcription factors (TFs) whose binding sites are enriched in a given set of promoters, the algorithm PRIMA (PRomoter Integration in Microarray Analysis (21)) of the Expander Software 6.1 (EXPression ANalyzer and DisplayER; (22)) was used. Genes that are significantly (p-value cut off is 0.05) changed greater than 2-fold were used for analysis. All genes from mouse (Ensembl release 42) were used as background set, the threshold of the p-value was set to 0.01 and the region was scanned from -3000 to +200.

**Metagene analysis**

The ´inflammation` and ´metabolism` metagenes were obtained as follows: (i) The overlap between deregulated genes (2-fold, p≥0.05, FDR adjusted) of all culture systems (M_C_, M_S_, S) and all in vivo models (CCl_4_, PHx, LPS) day 1 was determined. This resulted in 74 genes. (ii) Of these 74 genes, 19 and 28 belonged to the ´inflammation` and 28 ´metabolism` clusters, respectively, as defined in **Fig 5** (list of individual genes in **Suppl. Table 13**). The 19 inflammation and 28 metabolism associated genes were used to calculate the ´inflammation` and ´metabolism` metagenes, respectively. (iii) For this purpose, the expression level of each gene was normalized in the following way: the mean value (of the normalized gene expression values) of the 19 inflammation associated genes was defined as the ´inflammation` metagene. The same technique was applied to the metabolism associated genes. This calculation was performed in each independent biological replica (n=3 for cultivation and freshly isolated hepatocytes; n=5 for CCl_4_, PHx and LPS) and boxplots of the corresponding data are shown in **Fig. 6**. For the in vitro samples, (M_C_, M_S_, S) the metabolism and inflammation metagenes were calculated using deregulated genes after 1 day in culture. Similarly, in vivo samples (CCl_4_, PHx and LPS) were analyzed one day after administration of CCl_4_, LPS or after performing PHx. The time point of one day was chosen because this represents the earliest time point were a strong correlation (r>0.6) between all genes deregulated in vivo and in vitro was obtained (**Fig 4** and **Suppl. Fig 6**). In contrast, since proliferation of hepatocytes in mice only occurs approximately 2 days after administration of CCl_4_ or PHx, all calculations for the proliferation metagene shown in **Fig. 6** were done using the expression values of 2 days in culture and in the in vivo models CCl_4_ and PHx. For human hepatocytes and human disease liver models, a similar approach was applied, except that the top 15 strongest deregulated genes in each cluster group were used for establishing the corresponding metagenes.

**Interspecies (mouse/human) analysis of gene expression**

For establishing a correlation between similar genes deregulated in mouse and human hepatocytes, a spearman correlation analysis was performed as described (see Pearson’s rank correlation and odds ratio analysis section), using orthologous genes based on (23), with the Statistics Toolbox of MATLAB. The scatter plot obtained by this analysis was divided into four quadrants, representing upregulated genes in both mouse and human hepatocytes (Q1), downregulated genes in mouse hepatocytes which are upregulated in human hepatocytes (Q2), downregulated genes in both mouse and human hepatocytes (Q3) and upregulated genes in mouse hepatocytes which are downregulated in human hepatocytes (Q4) (Fig 7). A complete list of genes contained in each quadrant is provided in the Supplemental Tables section (**Suppl. Table 14**).

**cDNA synthesis and real time quantitative polymerase chain reaction analysis**

For complementary DNA synthesis, 2 µg of RNA were transcribed using the High Capacity cDNA reverse transcription kit (Applied Biosystems, Darmstadt, Germany). The resulting cDNA was diluted 10 fold and used as template for real-time PCR in an ABI Prism 7300 Sequence detection system (Applied Biosystems) using 2.5 µl of the diluted cDNA, TaqMan Universal PCR Master Mix and TaqMan primer probes. All TaqMan primer probes used are listed in Suppl. Table 15. The PCR conditions were 50°C for 2 min, 95°C for 10 min, followed by 40 cycles of 15 s at 95°C and1 min at 60°C for all PCR reactions. The relative mRNA content was normalized to GAPDH mRNA expression in each sample. For calculations of relative gene expression the 2^-ΔΔCt^ method was used (24). For mouse and human gene expression analyses, the expression levels in freshly isolated hepatocytes and hepatocytes in culture were normalized to the levels of healthy liver tissue. For human studies, the expression levels of cultivated hepatocytes were normalized to freshly isolated hepatocytes. The results shown correspond to means of three independent cell batches unless otherwise described.

**Purification of primary hepatocyte suspensions using Percoll**

In some experiments, freshly isolated hepatocytes were further purified from non-parenchymal cells by one, two or three additional Percoll centrifugations steps. Briefly, 40 million freshly isolated hepatocytes were mixed with Percoll (1.063 g/ml) (Easycoll®, Biochrom, Berlin, Germany), and centrifuged for 5 min at 50G, 4°C. The resulting cell pellet was washed twice by carefully re-suspending the cells in sterile PBS and centrifuged for 5 min at 50G, 4°C. Afterwards, the cell pellet was re-suspended in 10 ml of ´suspension medium` (1). This cell preparation was named ´1X Percoll`. Cell viability in this fraction was determined by trypan blue exclusion. From this cell suspension, two aliquots of 1 million cells each were collected for quantification of hepatocytes and non-parenchymal cells, by flow cytometry and hematoxylin-eosin (H&E) staining, respectively (procedures for flow cytometry and H&E staining are described below). The rest of the cells were used for cultivation in collagen sandwich. This procedure was repeated with the remaining cells for one or two additional times, herein referred to as ´2X- or 3X Percoll`.

**Fluorescent activated cell sorting (FACS) analysis**

The purity of each hepatocyte preparation before and after purification with Percoll was determined by fluorescence activated cell sorting (FACS) in a BD FACS-Calibur flow cytometer (Benton Dickinson, San Jose, CA) and the data were processed using the CellQuest Pro software (Becton Dickinson, San Jose, CA). A representative scatter plot analysis is shown in **Fig. 8A**. The large and granular hepatocyte population (designated here as R1) was clearly distinguishable from the smaller and less granular non-parenchymal cell population (designated as R2, **Suppl. Fig. 10A**).

**Hematoxylin-eosin staining of hepatocytes**

Primary mouse hepatocytes from standard or Percoll purified preparations were allowed to attach to collagen coated glass slides for 3h, washed two times with sterile PBS, and fixed with formaldehyde 4% in PBS, 10 min at room temperature. Afterwards, cells were permeabilized with triton X-100 0.5% in PBS (15 min, room temperature) and washed three times in PBS (5 min, room temperature). The slides were briefly soaked in distilled water, and stained with Hematoxylin (7.5 g/l) for 1 min at room temperature, and washed in distilled water for 15 min. The slides were shortly soaked in 1% HCl in 70% ethanol, stained with Eosin (1%) for 1 min, and washed in distilled water for 10 min. Afterwards, the samples were dehydrated by sequential incubation into increasing ethanol solutions (50%, 70%,90% and 100%, 2 min in each solution). Finally, the slides were incubated in Roti®-Histol for 2-3 min, mounted onto glass slides with Entellan® (Merck, Darmstadt, Germany) and air-dried overnight. Hepatocytes and non-parenchymal cells were identified with bright field microscopy, and counted on 10 randomly selected fields (200X magnification). A representative image is shown in **Suppl. Fig. 16A**.

**Hepatocyte spheroids**

Mouse hepatocyte spheroids were prepared using the hanging drop method in 96 well plates (GravityPLUS® 3D Culture and assay platform, InSphero®, Schlieren, Switzerland), with 2000 cells per well in a 40 µl drop of William’s E medium plus additives (2) and 20% FCS according to the manufacturer’s instructions (InSphero®, Schlieren, Switzerland). The spheroids were collected at days 1, 5, 7 and 14, and split into two fractions. One fraction was fixed in 4% PFA, and used for immunofluorescence staining of DPPIV and ICAM. Polymerized actin was detected by staining with rhodamine-labeled phalloidin (Life Technologies, Karlsruhe, Germany). Nuclei were stained with and 4`,6-diamidino-2-phenylindole (DAPI, Life Technologies, Karlsruhe, Germany) at 0.1 µg/ml for 30 min at room temperature. A second fraction was used for RNA isolation and subsequent qRT-PCR analysis of inflammation and metabolism markers. Details for immunostaining and RNA isolation are described below.

**Immunofluorescence**

Primary mouse hepatocytes were plated onto collagen coated glass slides and allowed to attach for 10 min (representing freshly isolated hepatocytes) or cultivated for a period of 1, 3, 5 or 7 days in M_C_, M_S_ or S configurations in 6 well culture plates as previously described (1). After the indicated time points, the medium was removed, the cells were washed twice with serum free William’s E medium, and immediately fixed with 4% PFA for 15 min at room temperature. Afterwards, the cells were permeabilized with triton X-100 0.5% in PBS for 10 min at room temperature, and washed three times with PBS, 5 min each. From here on, all incubations were performed in a humidified chamber. Unspecific binding sites were blocked by incubation with 5% BSA in PBS for 1h at room temperature. Afterwards, the blocking solution was removed, and the cells were incubated overnight at 4°C with the following antibodies in 5% BSA / PBS: anti HNF4α (Santa Cruz, Heidelberg, Germany), anti DPPIV (RnD Systems, Wiesbaden-Nordenstadt, Germany). Subsequently, the antibody solutions were removed and samples were washed three times for 10 min with PBS at room temperature. Afterwards, the samples were incubated for 1h at room temperature with following secondary antibodies labeled with Alexa 555 (for detection of HNF4α) or Alexa 647 (for detection of DPPIV, Maff or Klf6) dissolved in 5% BSA /PBS at 1:100 dilutions. After removing the secondary antibody solutions, the cells were washed three times for 10 min with PBS, and incubated with a mixture of rhodamine-labeled phalloidin at 5 U/ml (Biotium, Karlsruhe, Germany) and 4`,6-diamidino-2-phenylindole (DAPI, Life Technologies, Karlsruhe, Germany) at 0.1 µg/ml for 30 min at room temperature. Afterwards, the samples were washed three times with PBS for 10 min each, and mounted onto SuperfrostPlus glass slides (Menzel, Braunschweig, Germany) using SlowFade®Gold antifade reagent (Life Technologies, Karlsruhe, Germany), allowed to air dry for 30 min at room temperature, and fixed with nail polish. Hepatocyte spheroids were collected from 96 well plates at the indicated time points, fixed in 4%PFA and stained as previously described (25). For visualization of HNF4α in primary hepatocytes, the samples were analyzed on an Olympus BX41 epifluorescence microscope, and images were acquired using the Cell^M software (Olympus, Hamburg, Germany). For visualization of DPPIV and phallodin stainings in M_C_, S and spheroids, the images were acquired on an Olympus FV-1000 confocal microscope (Olympus, Hamburg, Germany). The excitation and emission wavelengths were used as specified by the manufacturer. The colors assigned to DPPIV, Phalloidin and DAPI were green, red and blue respectively.

**Multiplex analysis of signal transduction (Luminex)**

Protein extracts were collected from snap frozen liver tissue, freshly isolated hepatocytes and hepatocytes in M_C_ or S culture, using the Bio-Plex Cell Lysis kit (Bio-Rad, Hercules, CA, USA) following the manufacturer’s instructions. Phospho-protein levels were analyzed using the Bio-Plex system (Bio-Rad, Hercules, CA, USA) using total protein amount of 25 µg per sample and Multi-Plex-Assay consisting of antibodies against p-Akt (Ser473), p-c-jun (Ser63), p-ERK2(Thy185/Thr187), p-GSK3α/β (Ser9/Ser21), p-Hsp27 (Ser78), p-JNK (Thy193/Thr185), p-MEK1 (Ser217/Ser221), p-NFκB (Ser536), p-p38 (Thr180/Thy182), p-p70S6K (Thr421/Ser424) and pSTAT3 (Ser727). The assay was performed according to the manufacturer’s instructions with minor modifications (beads and detection antibodies were diluted 4-fold).

**Western blot analysis – Immunoblots**

Protein extracts and immunoblots were performed as previously described (26). Briefly, hepatocytes or snap frozen liver tissue were homogenized by sonication in ice cold radioimmunoprecipitation (RIPA) buffer (2% NP40, 0.5% DOC, 0.1% SDS, 250 mM NaCl, 2.5 mM EDTA, 50 mM Tris pH 7.2) supplemented with protease inhibitors (Protease inhibitor cocktail) and phosphatase inhibitors (Phosphatase inhibitor cocktail-I and phosphatase inhibitor cocktail-II), all from Sigma (München, Germany). The homogenates were incubated on ice for 30 min, and centrifuged for 10 min at 13.000 rpm, 4°C. The clear supernatant containing solubilized proteins was collected in a pre-chilled 1.5 ml tube. The protein concentration in the collected extracts was determined by a bicinchoninic acid (BCA) protein assay (Pierce, Rockford IL, USA). 30 to 50 µg of protein extract were heat denaturated in reducing conditions for 5 min at 95°C with Laemmli buffer. Afterwards, the samples were briefly chilled by placing the tubes on ice and briefly centrifuged at 13.000 rpm. The samples were loaded and resolved in 10% SDS-PAGE gels, and transferred to PVDF membranes. The membranes were blocked for 1h with 5% BSA in TBST buffer (pH 7.2), followed by overnight incubations at 4°C with the following antibodies (see Suppl. Table 17), all in 5% BSA-TBST. Afterwards, the membranes were washed three times for 10 min in TBST buffer at room temperature, followed by incubation with horse radish peroxidase labeled secondary antibodies in 5% BSA-TBST for 1h at room temperature. Subsequently, the membranes were washed three times in TBST, 10 min at room temperature. Bands were detected by chemiluminescense using the Western Lightning ECL substrate (Perkin Elmer, Waltham MA, USA), in a Fusion-FX7 imager (Vilbert Loumart, Eberhardzell, Germany) equipped with a CCD camera.

**siRNA transfection**

A set of four siRNA oligos (SmartPools, Dharmacon, Laffayette, CO, USA) were transfected into primary mouse hepatocytes four hours after plating onto collagen coated dishes, using Lipofectamin2000 (Invitrogen, Karlsruhe, Germany). The set of siRNA oligos selected were M-043530-01 (against mouse Klf6) and M-059047-02 (against mouse MafF). Control cells received an equal amount of scrambled (Scr) siRNA oligos (AllStar Negative Control (Qiagen, Hilden, Germany). Liposome-mediated transfection was performed for 12h, after which the cells were washed and further cultured with serum-free media. RNA was isolated 72h after transfection, and expression levels of Klf6, MafF and ´diagnostic genes` for the inflammation and mature liver function motifs were analyzed by real time qPCR, using expression levels in freshly isolated hepatocytes as reference.

**Statistics**

To evaluate whether bile acids had a significant influence on expression levels of Lcn2 and Bsep the non-parametric Mann-Whitney test (two-sided) was used. First each of the independent experiments was normalized to the controls by dividing the expression values (fold over liver) by the respective controls. IBM SPSS Statistics 22 was used to perform the two-sided Mann-Whitney test and differences between bile acid exposed samples and controls were considered as significant if the asymptotic p-value was smaller than 0.05. Since adjustment for multiple testing was not performed the results have to be considered as exploratory.

**Ethics committee approval for patient informed consent form for donation of liver resections**


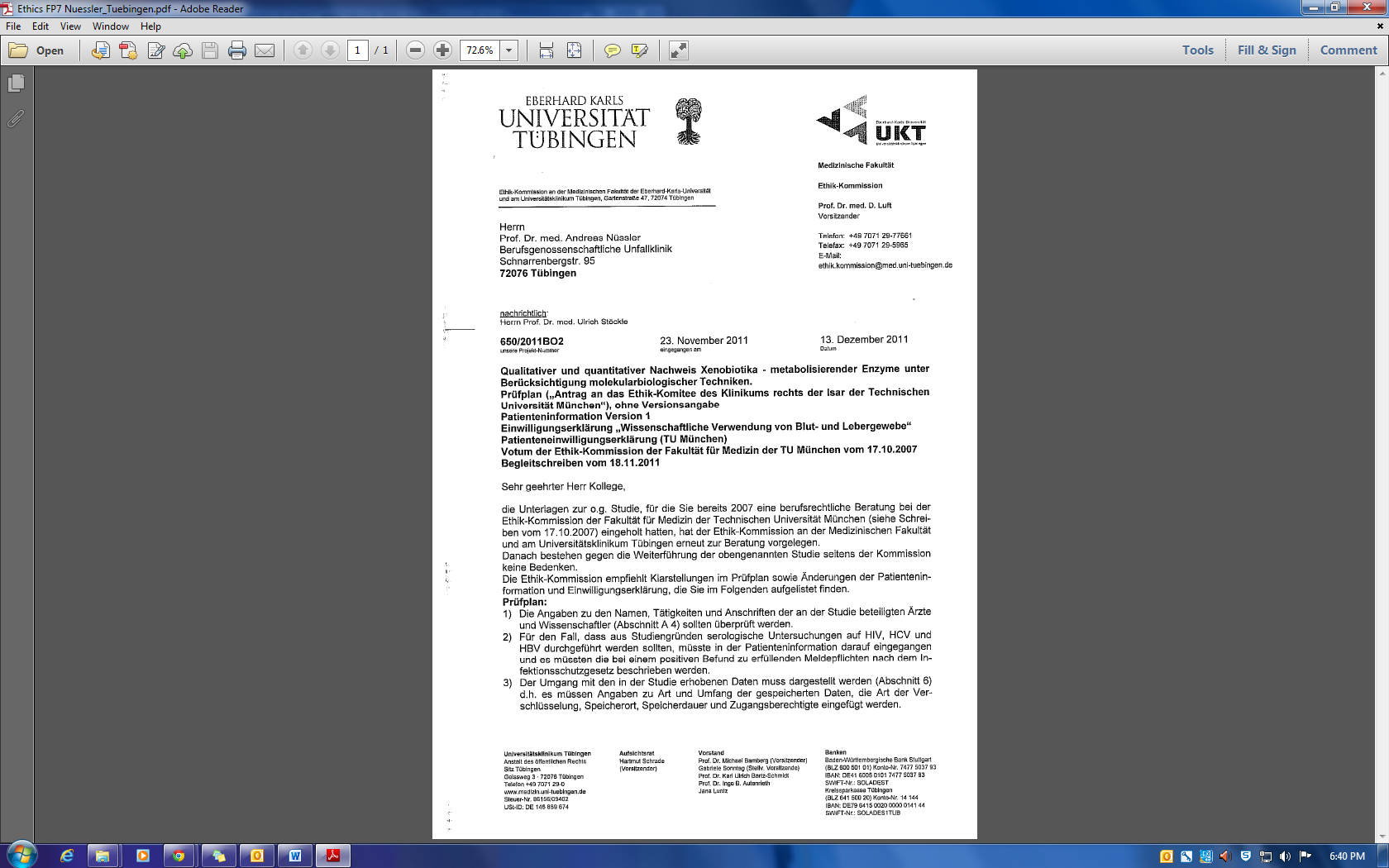


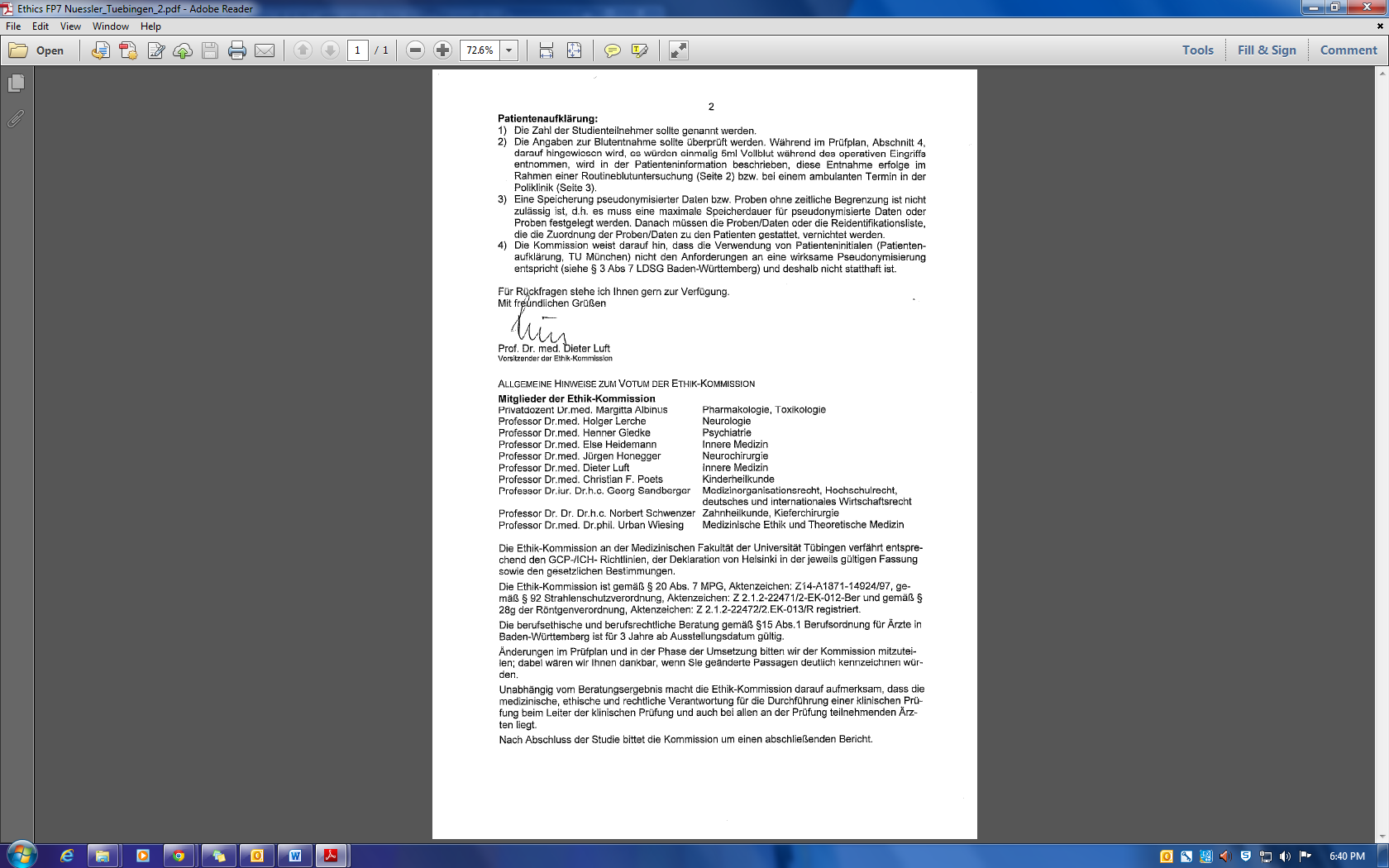


**References**

1. Godoy P, Hewitt NJ, Albrecht U, Andersen ME, Ansari N, Bhattacharya S, Bode JG, et al. Recent advances in 2D and 3D in vitro systems using primary hepatocytes, alternative hepatocyte sources and non-parenchymal liver cells and their use in investigating mechanisms of hepatotoxicity, cell signaling and ADME. Arch Toxicol 2013;87:1315-1530.

2. Hoehme S, Brulport M, Bauer A, Bedawy E, Schormann W, Hermes M, Puppe V, et al. Prediction and validation of cell alignment along microvessels as order principle to restore tissue architecture in liver regeneration. Proc Natl Acad Sci U S A 2010;107:10371-10376.

3. Madrahimov N, Dirsch O, Broelsch C, Dahmen U. Marginal hepatectomy in the rat - From anatomy to surgery. Annals of Surgery 2006;244:89-98.

4. Zellmer S, Schmidt-Heck W, Godoy P, Weng H, Meyer C, Lehmann T, Sparna T, et al. Transcription factors ETF, E2F, and SP-1 are involved in cytokine-independent proliferation of murine hepatocytes. Hepatology 2010;52:2127-2136.

5. BM. B. Low Level Analysis of High-density OligonucleotideArray Data: Background, Normalization and Summarization. . Berkeley: University of California 2004.

6. Gentleman RC, Carey VJ, Bates DM, Bolstad B, Dettling M, Dudoit S, Ellis B, et al. Bioconductor: open software development for computational biology and bioinformatics. Genome Biology 2004;5.

7. Smyth G: Limma: linear models for microarray data. In: Gentleman R C, V., Dudoit, S., Irizarry, R., Huber, W. , ed. , ed. 'Bioinformatics and Computational Biology Solutions using R and Bioconductor`. New York: Springer, 2005; 397-420.

8. Dai MH, Wang PL, Boyd AD, Kostov G, Athey B, Jones EG, Bunney WE, et al. Evolving gene/transcript definitions significantly alter the interpretation of GeneChip data. Nucleic Acids Research 2005;33.

9. Campos G, Schmidt-Heck W, Ghallab A, Rochlitz K, Putter L, Medinas DB, Hetz C, et al. The transcription factor CHOP, a central component of the transcriptional regulatory network induced upon CCl4 intoxication in mouse liver, is not a critical mediator of hepatotoxicity. Archives of Toxicology 2014;88:1267-1280.

10. Dapito DH, Mencin A, Gwak GY, Pradere JP, Jang MK, Mederacke I, Caviglia JM, et al. Promotion of Hepatocellular Carcinoma by the Intestinal Microbiota and TLR4. Cancer Cell 2012;21:504-516.

11. Morris SA, Cahan P, Li H, Zhao AM, Roman AKS, Shivdasani RA, Collins JJ, et al. Dissecting Engineered Cell Types and Enhancing Cell Fate Conversion via CellNet. Cell 2014;158:889-902.

12. Ventura-Holman T, Mamoon A, Subauste JS. Modulation of expression of RA-regulated genes by the oncoprotein v-erbA. Gene 2008;425:23-27.

13. Rodrigues RM, Heymans A, De Boe V, Sachinidis A, Chaudhari U, Govaere O, Roskams T, et al. Toxicogenomics-based prediction of acetaminophen-induced liver injury using human hepatic cell systems. Toxicol Lett 2016;240:50-59.

14. Godoy P, Schmidt-Heck W, Natarajan K, Lucendo-Villarin B, Szkolnicka D, Asplund A, Bjorquist P, et al. Gene networks and transcription factor motifs defining the differentiation of stem cells into hepatocyte-like cells. Journal of Hepatology 2015;63:934-942.

15. Farci P, Diaz G, Chen Z, Govindarajan S, Tice A, Agulto L, Pittaluga S, et al. B cell gene signature with massive intrahepatic production of antibodies to hepatitis B core antigen in hepatitis B virus-associated acute liver failure. Proceedings of the National Academy of Sciences of the United States of America 2010;107:8766-8771.

16. Moylan CA, Pang H, Dellinger A, Suzuki A, Garrett ME, Guy CD, Murphy SK, et al. Hepatic Gene Expression Profiles Differentiate Presymptomatic Patients With Mild Versus Severe Nonalcoholic Fatty Liver Disease. Hepatology 2014;59:471-482.

17. Yildiz G, Arslan-Ergul A, Bagislar S, Konu O, Yuzugullu H, Gursoy-Yuzugullu O, Ozturk N, et al. Genome-Wide Transcriptional Reorganization Associated with Senescence-to-Immortality Switch during Human Hepatocellular Carcinogenesis. Plos One 2013;8.

18. Bezdek JC, Hathaway RJ. Numerical Convergence and Interpretation of the Fuzzy-C-Shells Clustering-Algorithm. Ieee Transactions on Neural Networks 1992;3:787-793.

19. Matys V, Kel-Margoulis OV, Fricke E, Liebich I, Land S, Barre-Dirrie A, Reuter I, et al. TRANSFAC (R) and its module TRANSCompel (R): transcriptional gene regulation in eukaryotes. Nucleic Acids Research 2006;34:D108-D110.

20. Reimand J, Arak T, Vilo J. g:Profiler-a web server for functional interpretation of gene lists (2011 update). Nucleic Acids Research 2011;39:W307-W315.

21. Elkon R, Linhart C, Sharan R, Shamir R, Shiloh Y. Genome-wide in silico identification of transcriptional regulators controlling the cell cycle in human cells. Genome Research 2003;13:773-780.

22. Ulitsky I, Maron-Katz A, Shavit S, Sagir D, Linhart C, Elkon R, Tanay A, et al. Expander: from expression microarrays to networks and functions. Nature Protocols 2010;5:303-322.

23. Yue F, Cheng Y, Breschi A, Vierstra J, Wu WS, Ryba T, Sandstrom R, et al. A comparative encyclopedia of DNA elements in the mouse genome. Nature 2014;515:355-+.

24. Livak KJ, Schmittgen TD. Analysis of relative gene expression data using real-time quantitative PCR and the 2(T)(-Delta Delta C) method. Methods 2001;25:402-408.

25. Kim JY, Fluri DA, Marchan R, Boonen K, Mohanty S, Singh P, Hammad S, et al. 3D spherical microtissues and microfluidic technology for multi-tissue experiments and analysis. Journal of Biotechnology 2015;205:24-35.

26. Godoy P, Hengstler JG, Ilkavets I, Meyer C, Bachmann A, Muller A, Tuschl G, et al. Extracellular matrix modulates sensitivity of hepatocytes to fibroblastoid dedifferentiation and transforming growth factor beta-induced apoptosis. Hepatology 2009;49:2031-2043.
