# Supplementary material for: Gene network activity in cultivated primary hepatocytes is highly similar to diseased mammalian liver tissue
Source: Arch Toxicol. 2016 Jun 23;90(10):2513–29. doi: 10.1007/s00204-016-1761-4 (PMC5043005; doi:10.1007/s00204-016-1761-4)
Supplement: Supplementary file 20 — Supplementary material 20 (DOCX 25 kb) [file 204_2016_1761_MOESM20_ESM.docx]

**Legends for supplemental tables for the manuscript “Gene network activity in cultivated primary hepatocytes is highly similar to diseased mammalian liver tissue”**

Patricio Godoy^1,17,*,**^, Agata Widera^1,*^, Wolfgang Schmidt-Heck^2,*^, Gisela Campos^1^, Christoph Meyer^3^, Cristina Cadenas^1^, Raymond Reif^1^, Regina Stöber^1^, Seddik Hammad^1,3,16^, Larissa Pütter^1^, Kathrin Gianmoena^1^, Rosemarie Marchan^1^, Ahmed Ghallab^1,16^, Karolina Edlund^1^, Andreas Nüssler^4^, Wolfgang E. Thasler^5^, Georg Damm^6^, Daniel Seehofer^6^, Thomas S. Weiss^7^, Olaf Dirsch^8^, Uta Dahmen^9^, Rolf Gebhardt^10^, Umesh Chaudhari^11^, Kesavan Meganathan^11,18^, Agapios Sachinidis^11^, Jens Kelm^12^, Ute Hofmann^13^, René P. Zahedi^14^, Reinhard Guthke^2^, Nils Blüthgen^15^, Steven Dooley^3^, Jan G. Hengstler^1**^

**The following tables are included as supplemental material:**

1. **Supplemental Table 1**: Pearson correlation analysis of individual gene chips from mouse hepatocytes in monolayer confluent (CMC), monolayer subconfluent (CMS) or sandwich (CS) cultures and freshly isolated hepatocytes (FH).
2. **Supplemental Table 2**: Differentially expressed genes (DEGs) in mouse hepatocytes on monolayer confluent (M_C_) culture. Genes with a fold change ≥ 2-fold (FDR adjusted) in mouse hepatocytes in culture compared to freshly isolated hepatocytes (FH) are indicated with red (upregulated) or green (downregulated). The fold change and p-value for deregulated genes are indicated for each time point (e.g. Mc_Day1 vs FH). Genes are identified by gene symbol and name.
3. **Supplemental Table 3**: Differentially expressed genes (DEGs) in mouse hepatocytes on monolayer subconfluent (M_S_) culture. Genes with a fold change ≥ 2-fold (FDR adjusted) in mouse hepatocytes in culture compared to freshly isolated hepatocytes (FH) are indicated with red (upregulated) or green (downregulated). The fold change and p-value for deregulated genes are indicated for each time point (e.g. Ms_Day1 vs FH). Genes are identified by gene symbol and name.
4. **Supplemental Table 4**: Differentially expressed genes (DEGs) in mouse hepatocytes on sandwich (S) culture. Genes with a fold change ≥ 2-fold (FDR adjusted) in mouse hepatocytes in culture compared to freshly isolated hepatocytes (FH) are indicated with red (upregulated) or green (downregulated). The fold change and p-value for deregulated genes are indicated for each time point (e.g. S_Day1 vs FH). Genes are identified by gene symbol and name.
5. **Supplemental Table 5**: Differentially expressed genes (DEGs) in mouse liver after intraperitoneal lipopolysaccharide (LPS) administration. Genes with a fold change ≥ 2-fold (FDR adjusted) in mouse liver tissue 24h after intraperitoneal injection of 750 ng/kg LPS (LPS-D1 vs Control FH) are indicated with red (upregulated) or green (downregulated). The fold change and p-value for deregulated genes are indicated. Genes are identified by gene symbol and name.
6. **Supplemental Table 6**: Differentially expressed genes (DEGs) in mouse liver after partial hepatectomy. Genes with a fold change ≥ 2-fold (FDR adjusted) in mouse liver tissue at the indicated time points after 70% partial hepatectomy (e.g. 1h vs 0h) are indicated with red (upregulated) or green (downregulated). The fold change and p-value for deregulated genes are indicated (compared to time 0 after liver resection). Genes are identified by gene symbol and name.
7. **Supplemental Table 7**: Ranking analysis of differentially expressed genes in primary mouse hepatocytes in M_C_, M_S_ and S culture vs liver disease models (LPS, CCl_4_ intoxication, partial hepatectomy). The ranks were established by scoring the maximal gene expression values of all samples (e.g. CCl_4_ day 1, plus Mc day 1, plus Ms day 1, plus S day 1). The top genes correspond to those whose combined values are maximal. The vignette ´CCl_4_ and Mc, Ms, S` shows the rank analysis of cultivated mouse hepatocytes (day 1) versus mouse liver tissue on day 1 after CCl_4_ administration. The vignette ´PHx and (Mc, Ms, S)` shows the rank analysis of cultivated mouse hepatocytes (day 1) versus mouse liver tissue 12h and on day 1 after 70% partial hepatectomy. The vignette ´LPS and (Mc, Ms, S)` shows the rank analysis of cultivated mouse hepatocytes (day 1) versus mouse liver tissue 24h after LPS administration (i.p.). Genes with a fold change ≥ 2-fold (FDR adjusted) in mouse liver tissue or in cultivated hepatocytes (compared to healthy liver tissue) are indicated with red (upregulated) or green (downregulated). The fold change and p-value for deregulated genes are indicated. Genes are identified by gene symbol and name.
8. **Supplemental Table 8**: Top upregulated genes in primary mouse hepatocytes in culture and references to their role in inflammation.
9. **Supplemental Table 9**: Differentially expressed genes (DEGs) in primary mouse hepatocytes isolated at 1 day after CCl_4_ or LPS intraperitoneal administration. Genes with a fold change ≥ 2-fold (FDR adjusted) in mouse hepatocytes isolated from liver tissue after intraperitoneal injection of CCl_4_ on day 1 (CCl4-FH-D1 vs Control FH-D1) and day 3 (CCl4-FH-D3 vs Control FH-D1), and on day 1 after injection of 750 ng/kg LPS (LPS-D1 vs Control FH), are indicated with red (upregulated) or green (downregulated). The fold change and p-value for deregulated genes compared to freshly isolated hepatocytes from healthy liver (Control FH) are indicated. Genes are identified by gene symbol and name.
10. **Supplemental Table 10**: Clusters of differentially expressed genes in cultivated mouse hepatocytes. Gene clusters generated as described in Suppl. Methods (page 6). The genes belonging to each cluster are listed in each vignette. Deregulated genes not fitting in any cluster were classified as outliers and listed in vignette “outlier”. Dark red or green cells indicate fold change ≥ 2. Light red or green cells indicate fold change ≥ 1.5. Genes are identified by symbol and name.
11. **Supplemental Table 11**: Gene set enrichment analysis (GSEA) on gene clusters representing differentially expressed genes on mouse hepatocytes. All Gene Ontology (GO), KEGG pathway (KEGG) and Transcription Factor Binding Site (TBS) enrichment analyses are shown in separate vignettes. The vignette “GO-MF” contains gene ontology annotations for “Molecular Function” overrepresented in each cluster group. Column A: Gene ontology annotation identification (GO:ID). Column B: GO annotation description. Column C: number of genes (size) belonging to each GO annotation. For each cluster group, the number of genes belonging to the GO annotation list (count) and overrepresentation score (p-value) are indicated (grey cells = p-value<0.05, FDR-adjusted). Vignette “GO-MF (Gene)” includes an additional column containing the list of genes identified as belonging to the corresponding GO annotations. The vignette “GO-BP” contains gene ontology annotations for “Biological Process” overrepresented in each cluster group, and vignette “GO-BP (Gene)” includes an additional column containing the list of genes identified as belonging to the corresponding GO annotations. The vignette “GO-CC” contains gene ontology annotations for “Cellular Component” overrepresented in each cluster group, and vignette “GO-CC (Gene)” includes an additional column containing the list of genes identified as belonging to the corresponding GO annotations. Vignette “TFBS-PRIMA” contains TFBS overrepresented in each cluster group. Column A: Transcription factor binding site annotation (TRANSFAC database). For each cluster group, the number of genes (count) containing a binding site for the corresponding TF annotation, overrepresentation score (p-value and enrichment) are indicated (grey cells = p-value<0.05, FDR-adjusted). Vignette “TFBS-PRIMA (Gene)” includes an additional column containing the list of genes identified as belonging to the corresponding TF annotations.
12. **Supplemental Table 12**: Clusters of differentially expressed genes in cultivated human hepatocytes compared to freshly isolated hepatocytes. The genes belonging to each cluster are listed in each vignette. Deregulated genes not fitting in any cluster were classified as outliers and listed in vignette “outlier”. Dark red or green cells indicate fold change ≥ 2. Light red or green cells indicate fold change ≥ 1.5. Genes are identified by symbol and name.
13. **Supplemental Table 13**: Gene set enrichment analysis (GSEA) on gene clusters representing differentially expressed genes on human hepatocytes. Vignette “GO BKL” contains gene ontology annotations overrepresented in each cluster group. Column A: Gene ontology annotation identification (GO:ID). Column B: GO annotation description. Column C: number of genes (size) belonging to each GO annotation. For each cluster group, the number of genes belonging to the GO annotation list (count) and overrepresentation score (p-value) are indicated (grey cells = p-value<0.05, FDR-adjusted). Vignette “GO BKL (gene)” includes an additional column containing the list of genes identified as belonging to the corresponding GO annotations. Vignette “TFBS-PRIMA” contains TFBS overrepresented in each cluster group. Column A: Transcription factor binding site annotation (TRANSFAC database). For each cluster group, the number of genes (count) containing a binding site for the corresponding TF annotation, overrepresentation score (p-value and enrichment) are indicated (grey cells = p-value<0.05, FDR-adjusted). Vignette “TFBS-PRIMA (Gene)” includes an additional column containing the list of genes identified as belonging to the corresponding TF annotations.
14. **Supplemental Table 14**: List of genes constituting the metagene analysis in mouse and human hepatocytes and disease liver tissue
15. **Supplemental Table 15**: Orthologous genes deregulated in mouse and human hepatocytes in culture (day 1). Mouse and human orthologous genes with similar expression in monolayer confluent or sandwich culture (as shown in the quadrants of Fig 7A) are listed in separate vignettes for each quadrant and culture system. Vignette “Q1_Mc” lists the genes included in quadrant 1 on monolayer cultures; vignette “Q2_Mc” lists the genes included in quadrant 2 on monolayer cultures; vignette “Q3_Mc” lists the genes included in quadrant 3 on monolayer cultures; vignette “Q4_Mc” lists the genes included in quadrant 4 on monolayer cultures. Vignette “Q1_S” lists the genes included in quadrant 1 on sandwich cultures; vignette “Q2_S” lists the genes included in quadrant 2 on sandwich cultures; Vignette “Q3_S” lists the genes included in quadrant 3 on sandwich cultures; Vignette “Q4_S” lists the genes included in quadrant 4 on sandwich cultures. Mouse and human genes are indicated by their EntrezID, gene symbol and gene name.
16. **Supplemental Table 16**: List of orthologous interspecies genes constituting the interspecies metagene analysis. The genes in quadrants 1 to 4 are listed in vignettes Q1, Q2, Q3 and Q4, respectively. The table indicates the Entrez-ID, gene symbol, gene description, expression level (fold over fresh hepatocytes), and rank based on expression levels. The column “sum” indicates the sum of gene expression level (fold) for each gene in human and mouse hepatocytes. The column “sum rank” indicates the sum of rank values for each individual gene in human and mouse hepatocytes. The top then genes for both human and mouse hepatocytes were established by the lowest sum rank values (orange cells), and were used to calculate the metagene scores in Fig 7.
17. **Supplemental Table 17**: antibodies used for western blot and immunostaining
18. **Supplemental Table 18**: TaqMan gene expression assays use in this study.
