# Supplementary material for: Gene network activity in cultivated primary hepatocytes is highly similar to diseased mammalian liver tissue
Source: Arch Toxicol. 2016 Jun 23;90(10):2513–29. doi: 10.1007/s00204-016-1761-4 (PMC5043005; doi:10.1007/s00204-016-1761-4)
Supplement: Supplementary file 21 — Supplementary material 21 (DOCX 12128 kb) [file 204_2016_1761_MOESM21_ESM.docx]

**Supplemental figures for the manuscript “Gene network activity in cultivated primary hepatocytes is highly similar to diseased mammalian liver tissue”**

Patricio Godoy^1,17,*,**^, Agata Widera^1,*^, Wolfgang Schmidt-Heck^2,*^, Gisela Campos^1^, Christoph Meyer^3^, Cristina Cadenas^1^, Raymond Reif^1^, Regina Stöber^1^, Seddik Hammad^1,3,16^, Larissa Pütter^1^, Kathrin Gianmoena^1^, Rosemarie Marchan^1^, Ahmed Ghallab^1,16^, Karolina Edlund^1^, Andreas Nüssler^4^, Wolfgang E. Thasler^5^, Georg Damm^6^, Daniel Seehofer^6^, Thomas S. Weiss^7^, Olaf Dirsch^8^, Uta Dahmen^9^, Rolf Gebhardt^10^, Umesh Chaudhari^11^, Kesavan Meganathan^11,18^, Agapios Sachinidis^11^, Jens Kelm^12^, Ute Hofmann^13^, René P. Zahedi^14^, Reinhard Guthke^2^, Nils Blüthgen^15^, Steven Dooley^3^, Jan G. Hengstler^1**^


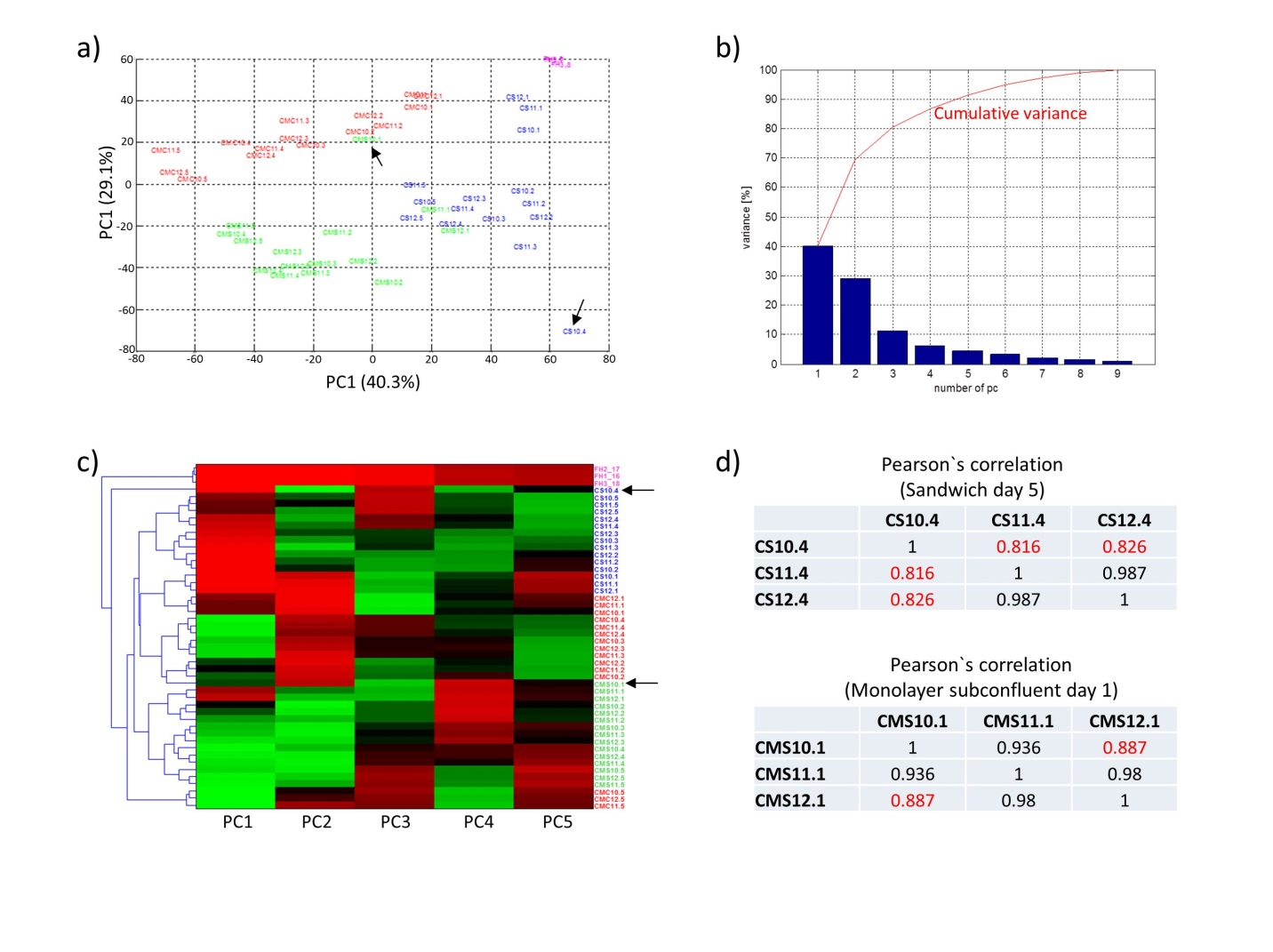


**Supplemental figure 1:** Identification of outliers by principal component analysis and Pearson correlation. a) Principal component analysis with PC1 and PC2 (representing 69.4% of the variance). One sample corresponding to S day 5 (CS10.4) and one to MS day 1 (CMS12.1) are highlighted by black arrows and identified as outliers to their respective groups. b) The blue bars represent percentage of variance in each principal component, and the red line indicates the cumulative variance. The first five PC constitute over 90% of the variance of the dataset. c) Heatmap of all samples based on the first five PCs. Samples from each cultivation system and time point cluster together as triplets with the exception of CS10.4 and CMS12.1, indicated by arrows. D) Person correlation analysis of time-matched samples in collagen sandwich day 5 (CS10.4, CS11.4, CS12.4) and in monolayer subconfluent day 1 (CMS10.1, CMS11.1, CMS12.1). The samples CS10.4 and CMS12.1 show correlations below 0.9 and were thus considered outliers. The complete analysis for all samples and time points can be found in supplemental table 1.


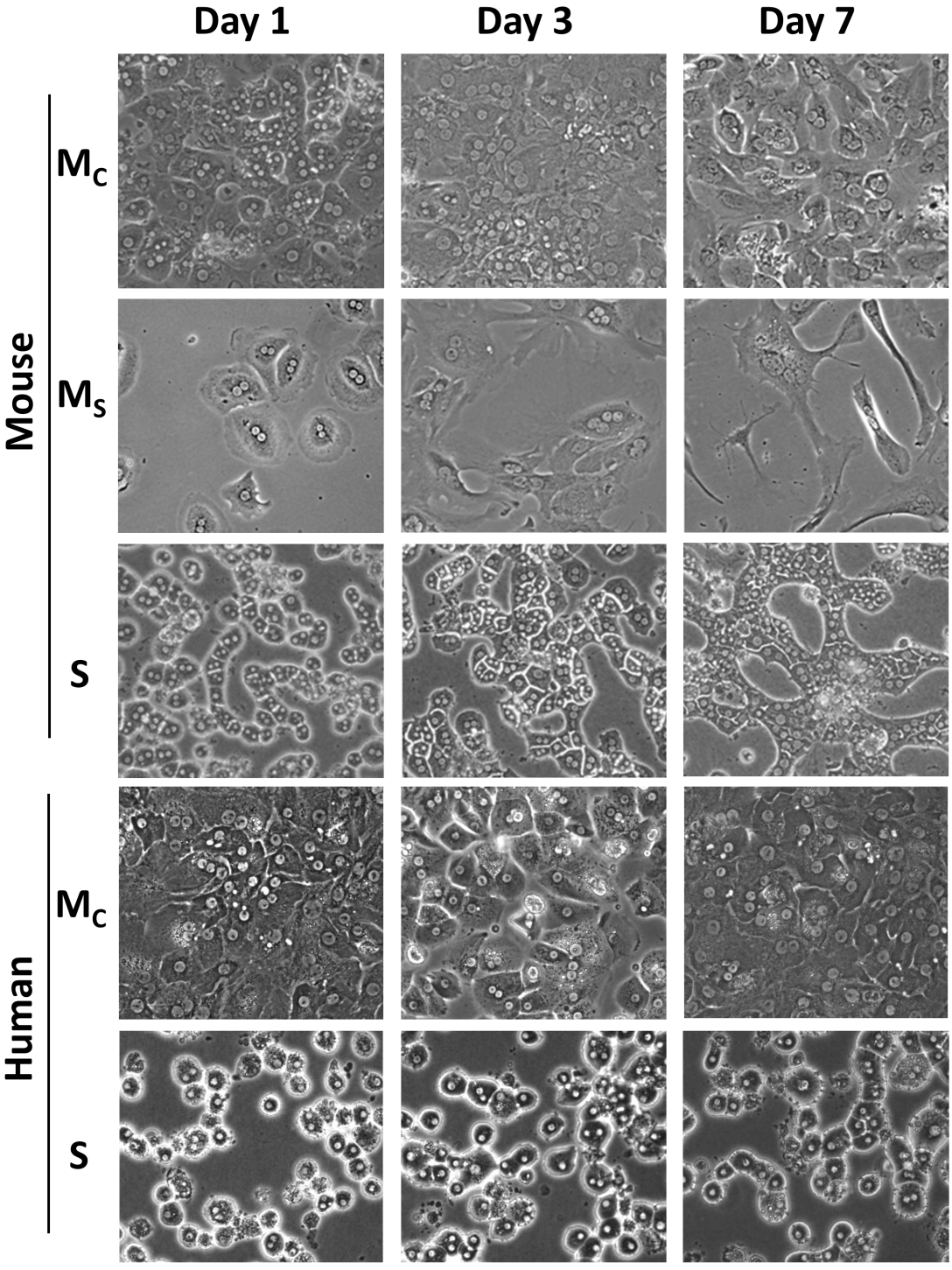


**Supplemental figure 2:** Morphology of primary mouse and human hepatocytes in culture. The images correspond to phase contrast photographs of hepatocytes in monolayer confluent (M_C_), monolayer subconfluent (M_S_) and sandwich (S) cultures, for the indicated periods of time. M_S_ cultured mouse hepatocytes present typical features of epithelial to mesenchymal transition (EMT) such as lamellipodia and filopodia, which become more prominent after longer cultivation periods. M_C_ cultures maintain a cuboidal morphology during the first day in culture. However, after longer cultivation periods, EMT features are also observed. Sandwich cultured hepatocytes present the most stable morphology, maintaining a cuboidal shape and distinct bile canaliculi even after longer periods in culture.


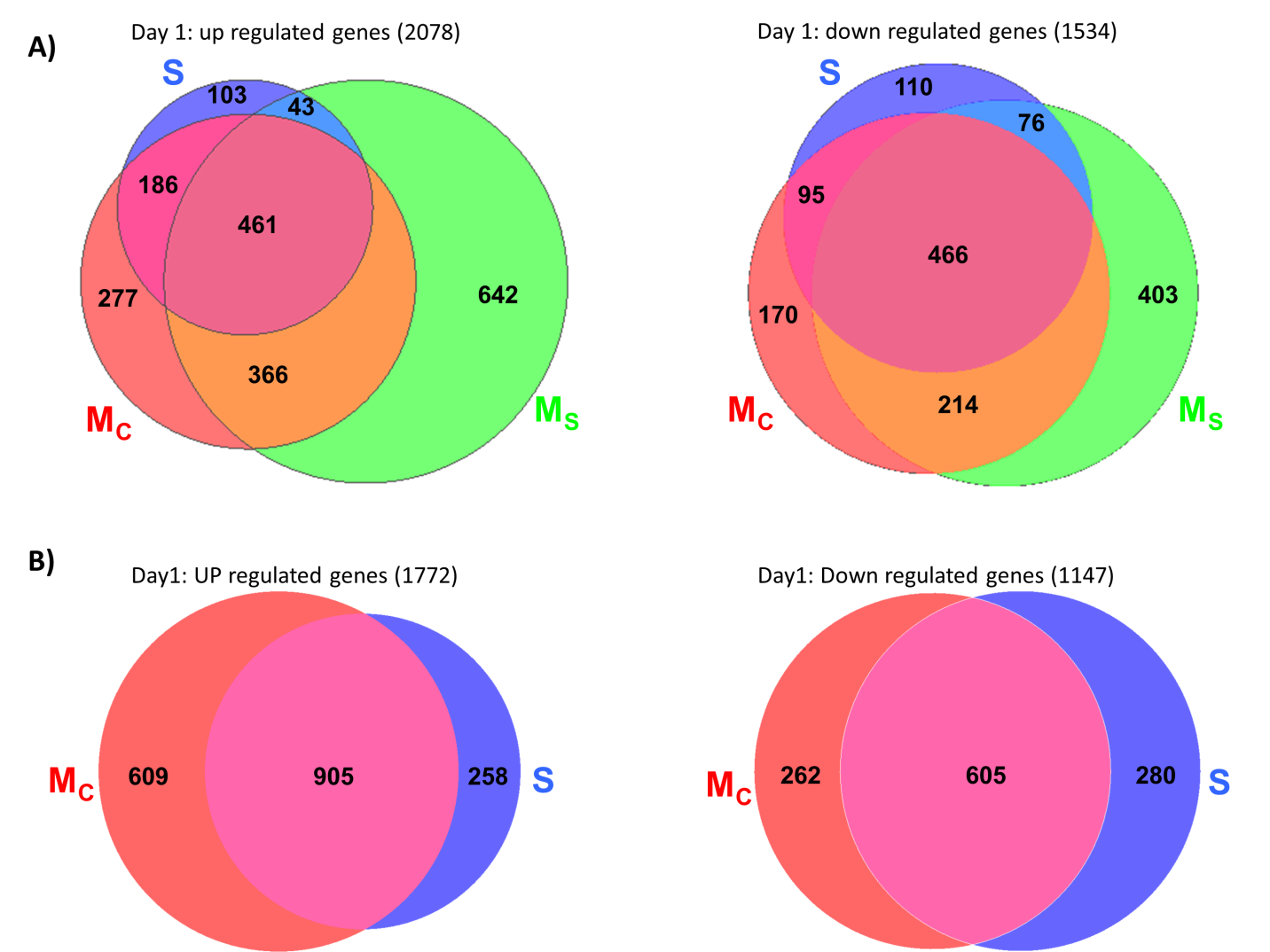


**Supplemental figure 3**: Overlap between deregulated genes in all culture systems. Venn diagrams reveal a high overlap between genes up or downregulated over freshly isolated hepatocytes (≥ 2-fold, FDR adjusted) in the three culture systems. **A)** Deregulated genes in mouse hepatocytes on monolayer confluent (M_C_), monolayer subconfluent (M_S_) and sandwich (S) culture on day 1. **B)** Deregulated genes in human hepatocytes in monolayer confluent (M_C_), and sandwich (S) culture on day 1. The total number of deregulated genes (up and down respectively) is indicated on top of the diagrams.


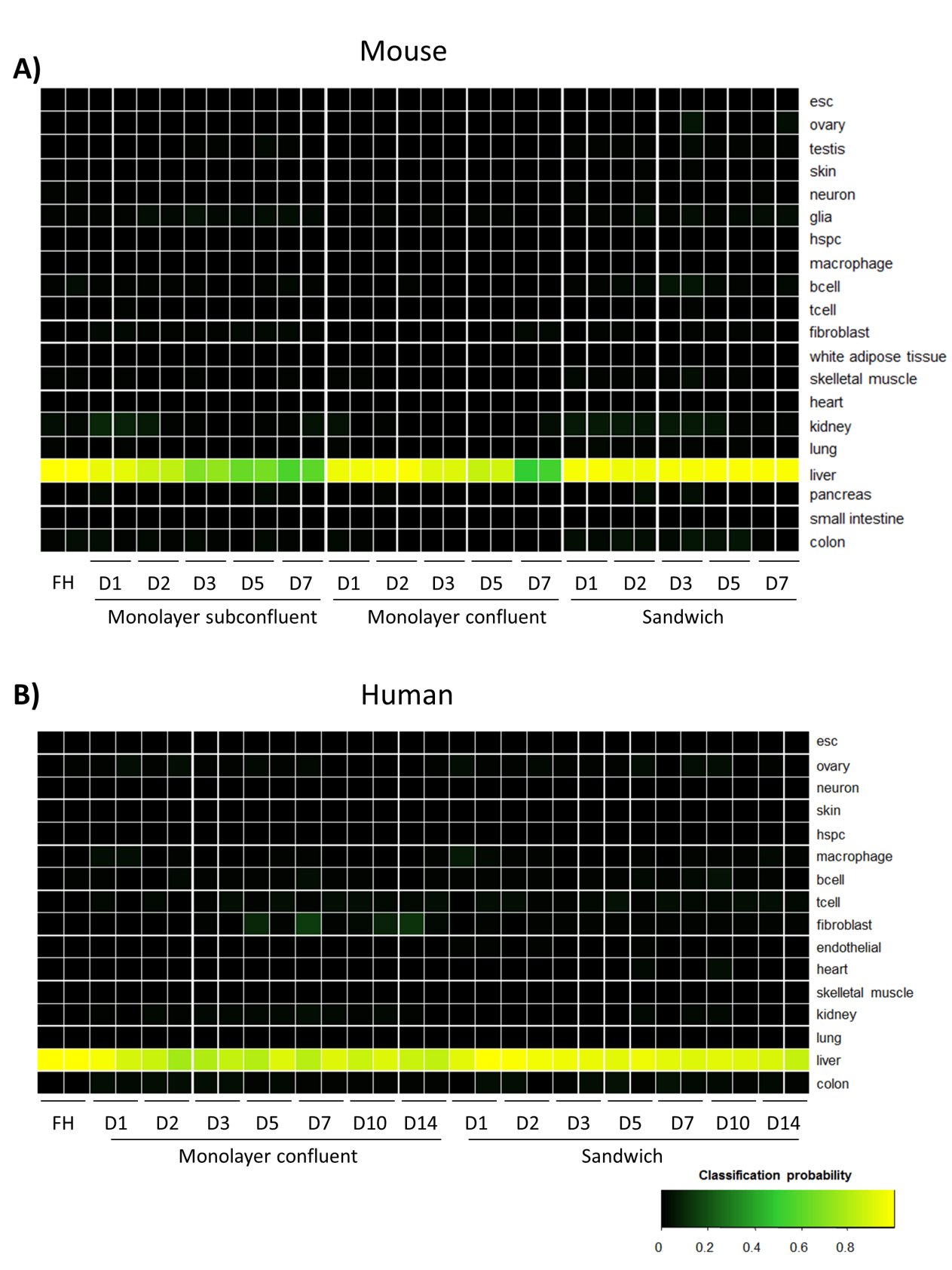


**Supplemental figure 4:** Cell/tissue identity analysis by CellNet in cultivated mouse **(A)** and human **(B)** primary hepatocytes. The heat maps show the cell and tissue classification probability on freshly isolated primary hepatocytes (FH) and primary hepatocytes in monolayer confluent, subconfluent and sandwich culture for the indicated time (days). Gene expression profiles of each sample were analyzed with the CellNet algorithm (see supplemental methods) and compared to the training expression profiles defining one of 16 (human) or 20 (mouse) tissues or cells, as described in Cahan et al [1]. All culture systems maintained a high ´liver` classification score throughout the entire cultivation time investigated, however a marked decrease was observed for this score in monolayer cultures, particularly at late periods in culture and most prominently in subconfluent conditions. Of the remaining cell/tissue identities, only the ´fibroblast` score was slightly increased in monolayer cultures. Classification probability scores rank from 0 to 1 (see classification probability color scale).


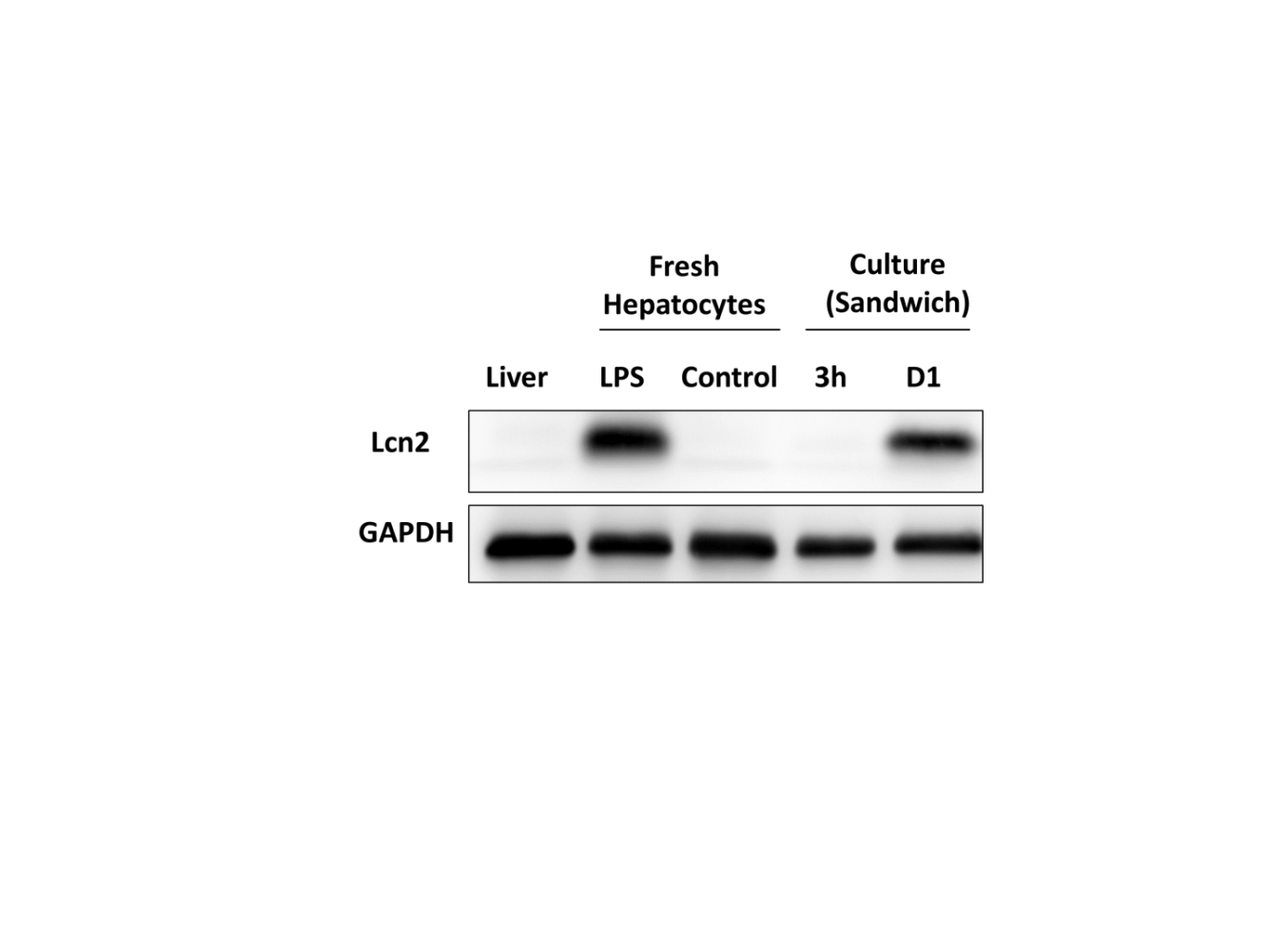


**Supplemental figure 5**: Western blot analysis of Lcn2 expression in hepatocytes and in inflamed liver tissue. Protein extracts from healthy liver tissue and freshly isolated hepatocytes were used as negative controls, while freshly isolated hepatocytes from mice treated for 24h with LPS (intraperitoneally) were used as positive control. Lcn2 expression is clearly observed in sandwich cultured hepatocytes at day 1. GAPDH was used as loading control. Representative picture of three independent experiments.


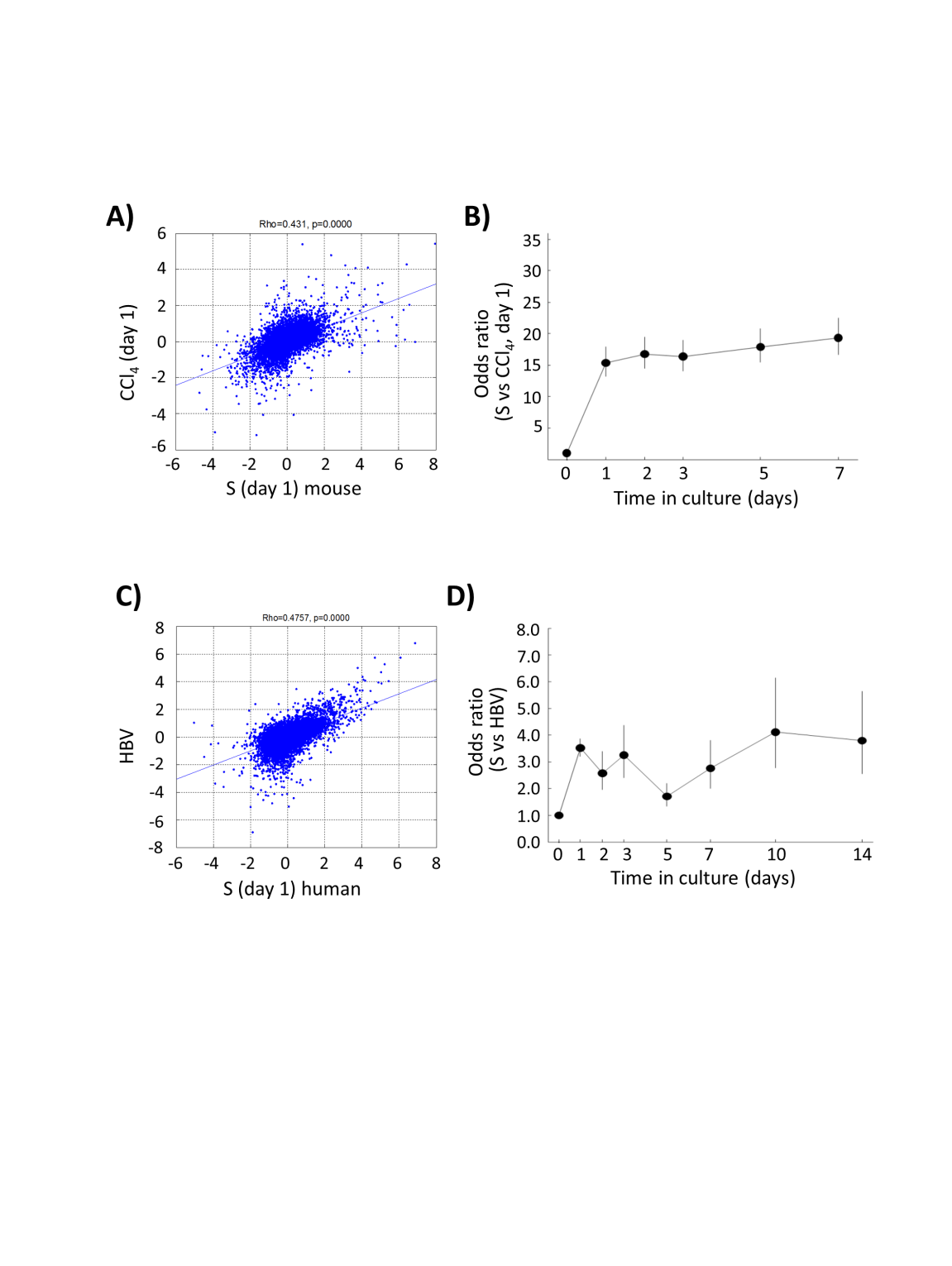


**Supplemental figure 6**: Gene expression correlations between in vivo mouse models of liver disease and cultivation-induced stress in sandwich cultured mouse hepatocytes. **A)** Scatter plots showing all differentially expressed genes in primary mouse hepatocytes on sandwich culture (day 1) versus mouse liver tissue on day 1 after CCl_4_ administration (log2 scale). **B)** Odds ratio analysis between differentially expressed genes in primary mouse hepatocytes (for the indicated time in sandwich culture) and mouse liver tissue on day 1 after CCl_4_ administration. **C)** Scatter plots showing all differentially expressed genes in primary human hepatocytes on sandwich culture (day 1) versus human HBV-infected liver tissue (log2 scale). **B)** Odds ratio analysis between differentially expressed genes in primary human hepatocytes (for the indicated time in sandwich culture) and human HBV-infected liver tissue.


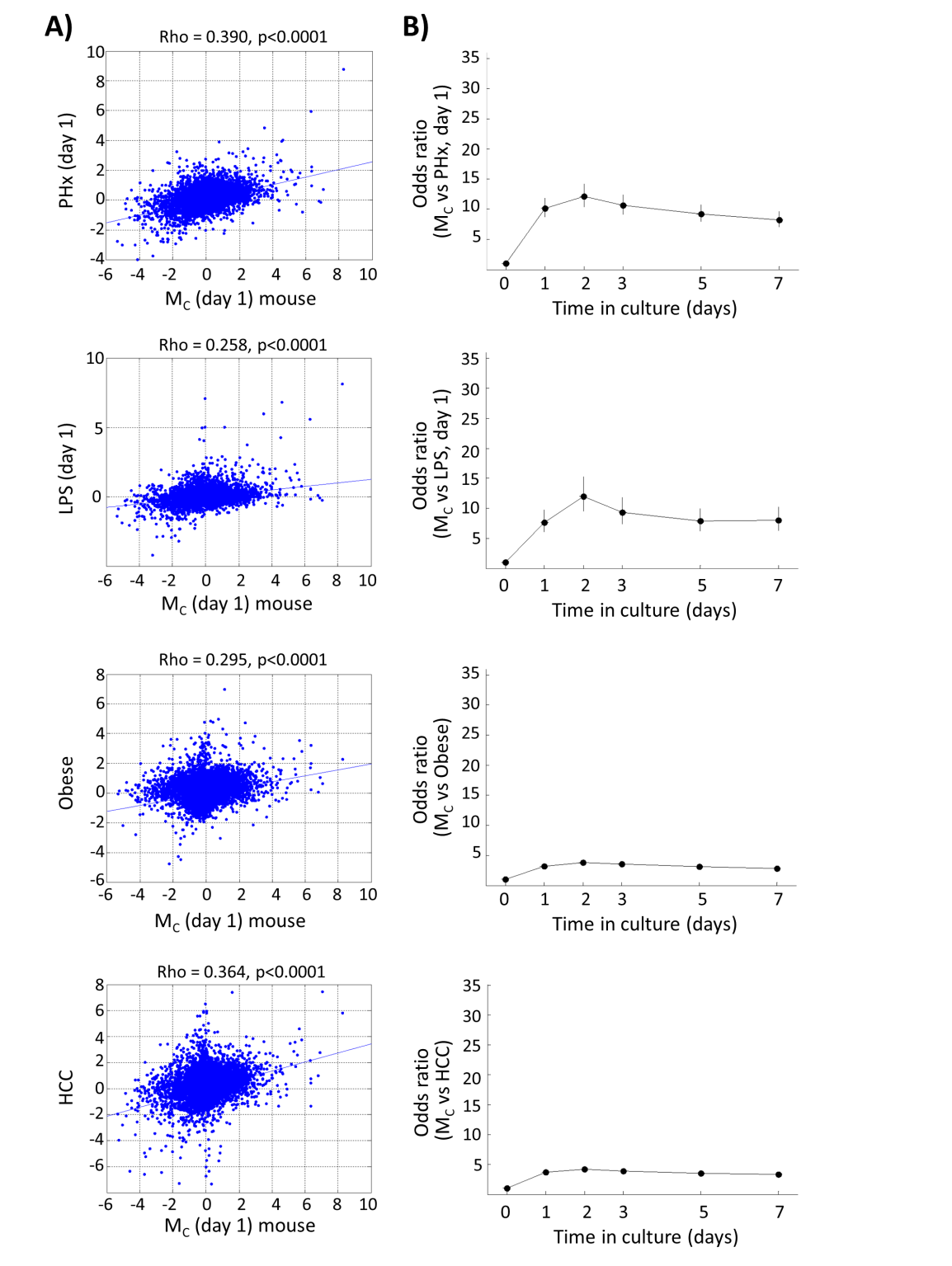


**Supplemental figure 7**: Gene expression correlations between in vivo mouse models of liver disease and cultivation-induced stress in monolayer cultured mouse hepatocytes. **A)** Scatter plots showing all differentially expressed genes in primary mouse hepatocytes on monolayer confluent culture (day 1) versus mouse liver tissue after partial hepatectomy, (day 1), LPS-induced inflammation (day 1), obese (fatty liver), and hepatocellular carcinoma (HCC) (log2 scale). **B)** Odds ratio analysis between differentially expressed genes in primary mouse hepatocytes (for the indicated time in monolayer confluent culture) and mouse liver tissue from the disease models shown in A.

**
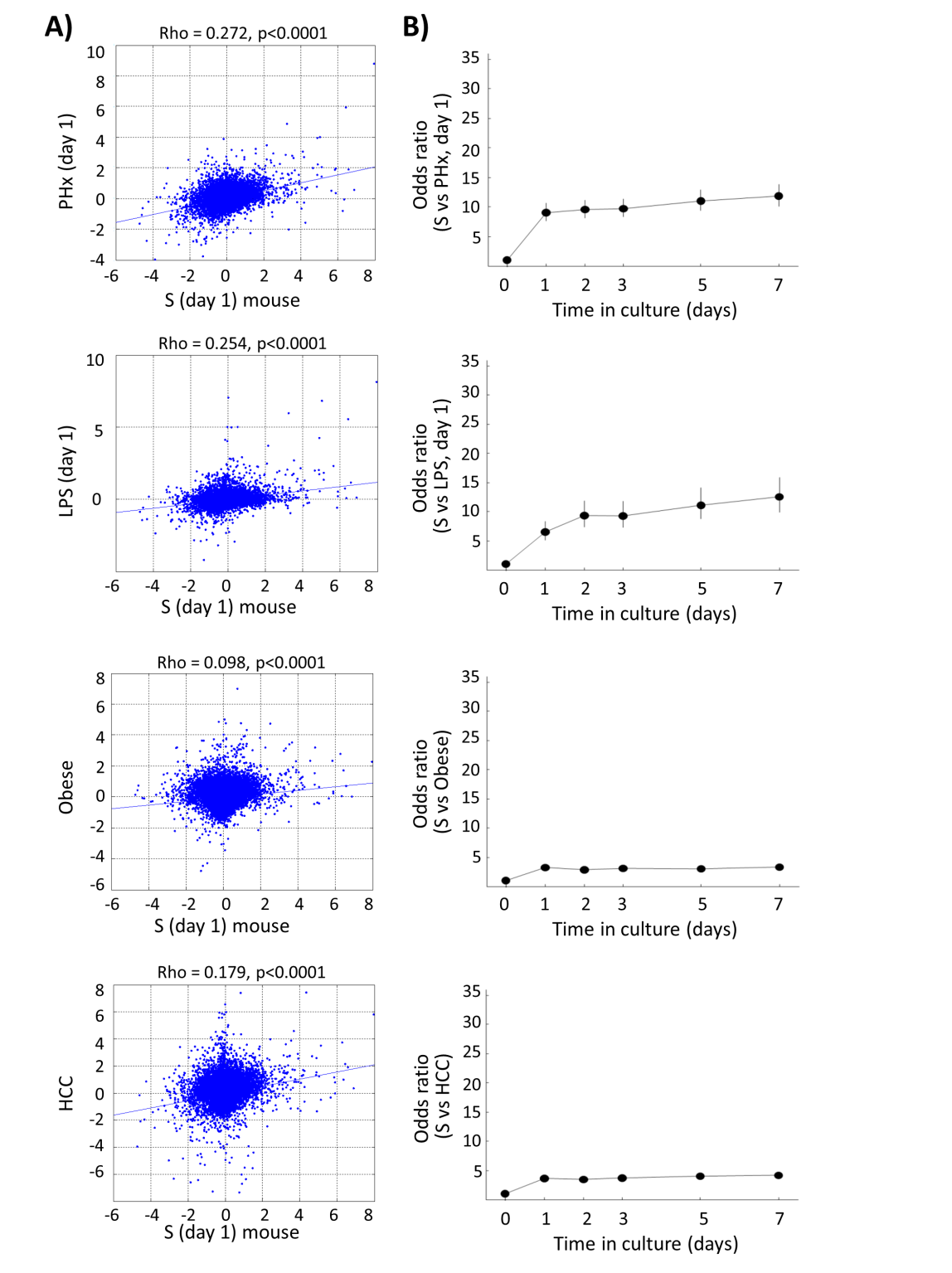
**

**Supplemental figure 8**: Gene expression correlations between in vivo mouse models of liver disease and cultivation-induced stress in sandwich cultured mouse hepatocytes. **A)** Scatter plots showing all differentially expressed genes in primary mouse hepatocytes on sandwich culture (day 1) versus mouse liver tissue after partial hepatectomy, (day 1), LPS-induced inflammation (day 1), obese (fatty liver), and hepatocellular carcinoma (HCC) (log2 scale). **B)** Odds ratio analysis between differentially expressed genes in primary mouse hepatocytes (for the indicated time in sandwich culture) and mouse liver tissue from the disease models shown in A.


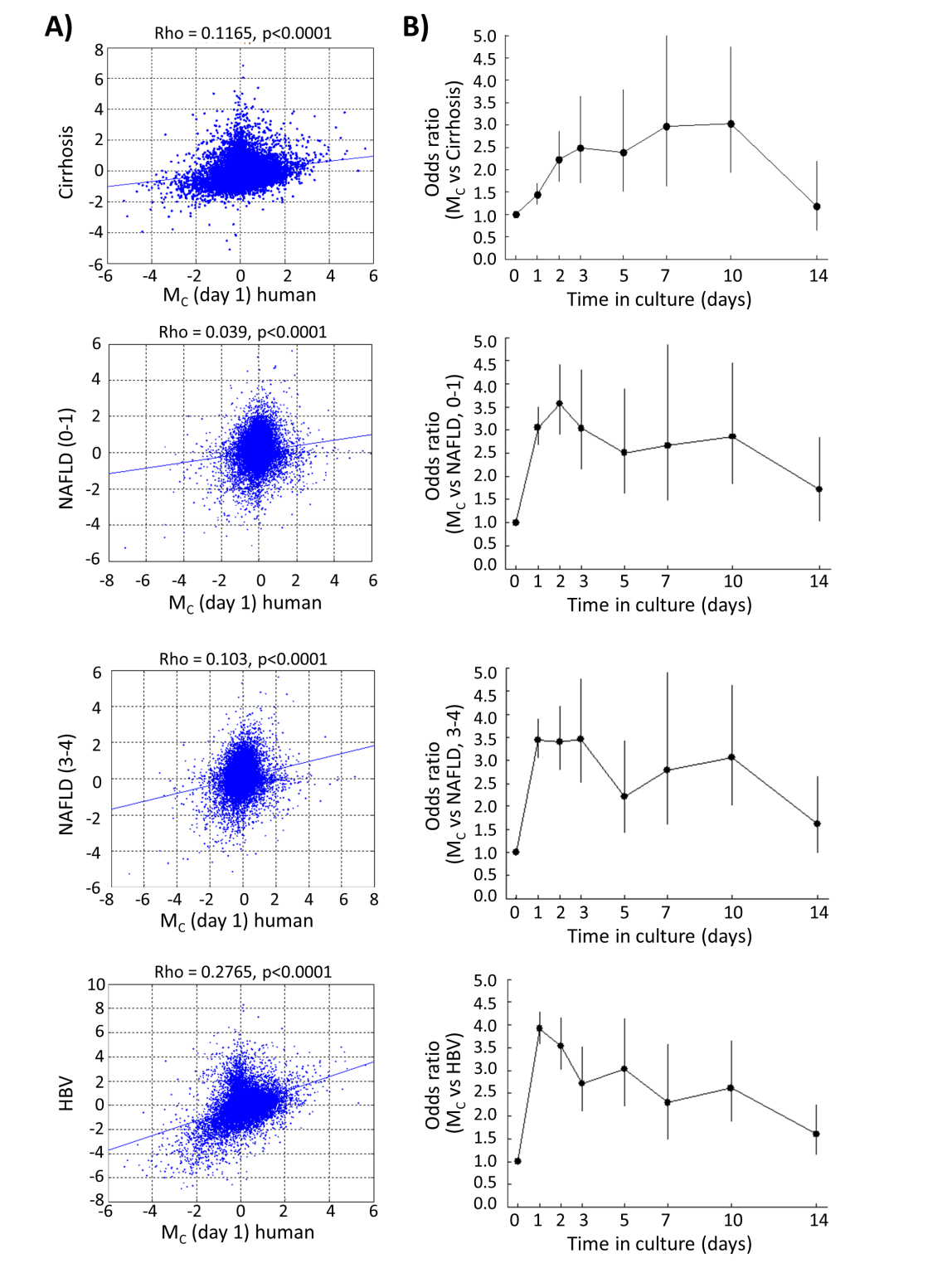


**Supplemental figure 9**: Gene expression correlations between diseased human liver and cultivation-induced stress in monolayer cultured human hepatocytes. **A)** Scatter plots showing all differentially expressed genes in primary human hepatocytes on monolayer confluent culture (day 1) versus human liver tissue from cirrhosis, non-alcoholic fatty liver disease (NAFLD) (stage 0-1), NAFLD (stage 3-4), and hepatitis B virus (HBV) infection (log2 scale). **B)** Odds ratio analysis between differentially expressed genes in primary human hepatocytes (for the indicated time on monolayer confluent culture) and human liver tissue from the disease conditions shown in A.

**
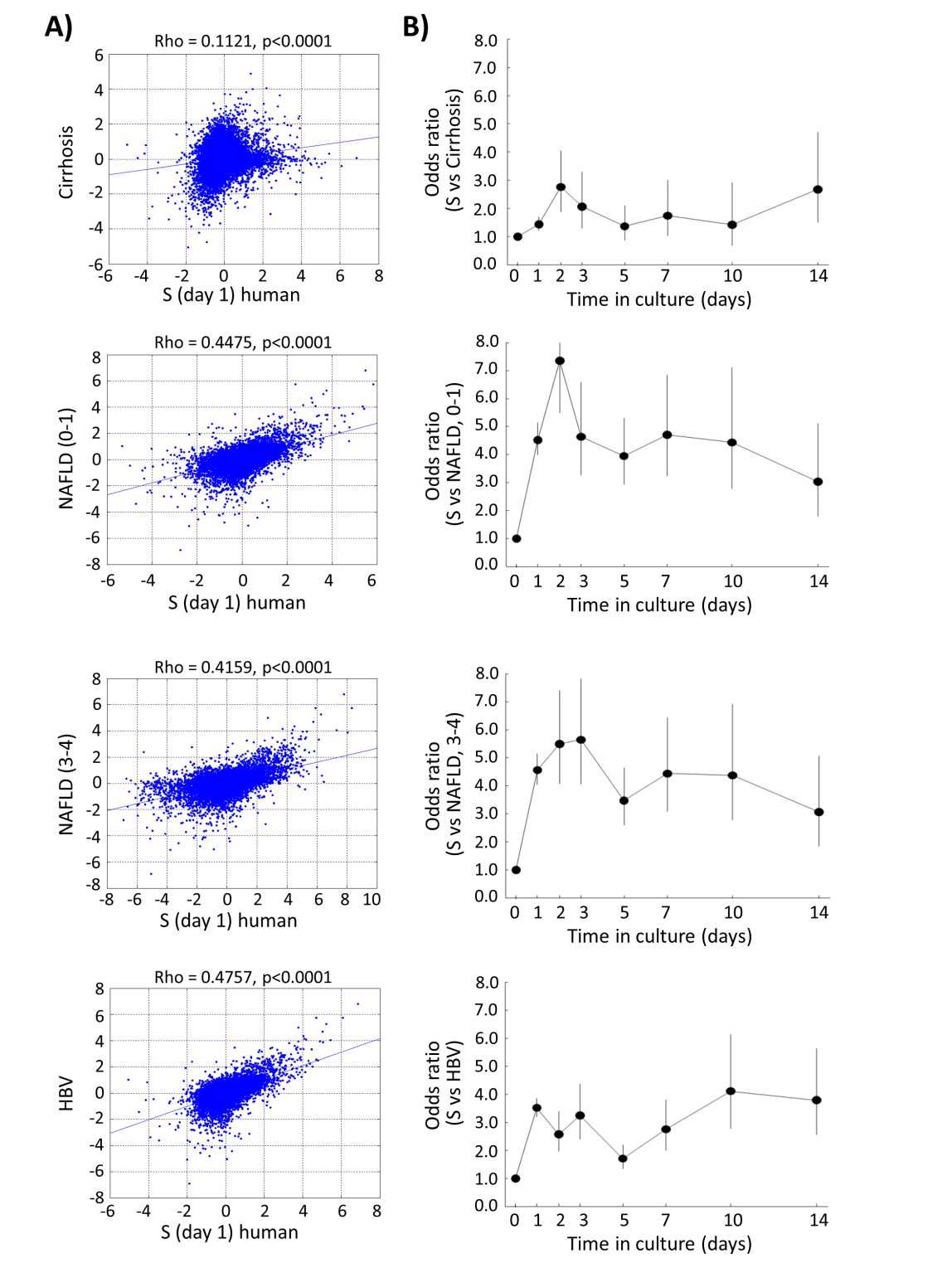
**

**Supplemental figure 10**: Gene expression correlations between diseased human liver and cultivation-induced stress in sandwich cultured human hepatocytes. **A)** Scatter plots showing all differentially expressed genes in primary human hepatocytes on sandwich culture (day 1) versus human liver tissue from cirrhosis, non-alcoholic fatty liver disease (NAFLD) (stage 0-1), NAFLD (stage 3-4), and hepatitis B virus (HBV) infection (log2 scale). **B)** Odds ratio analysis between differentially expressed genes in primary human hepatocytes (for the indicated time on sandwich culture) and human liver tissue from the disease conditions shown in A.


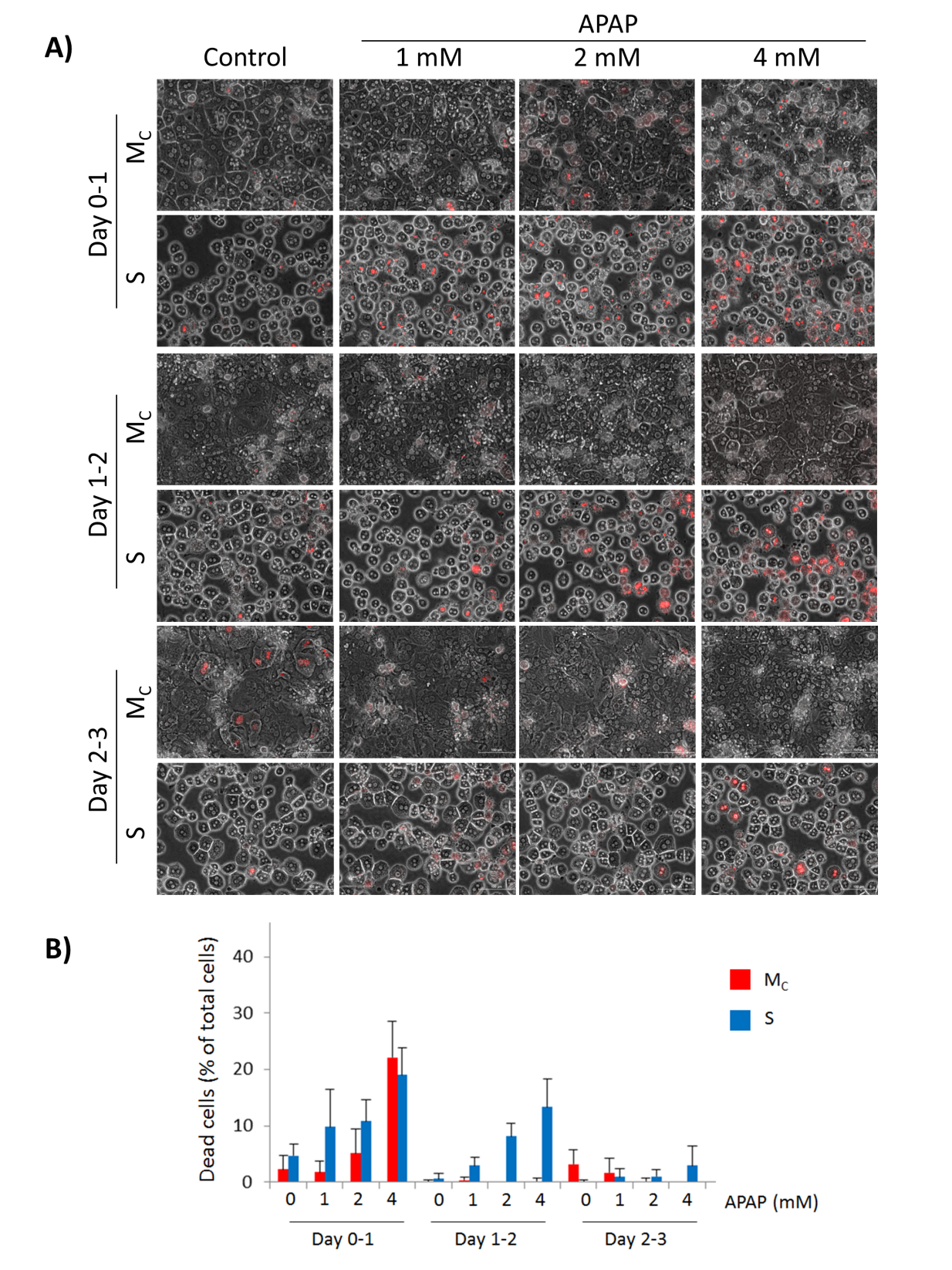


**Supplemental figure 11**: Enhanced sensitivity to hepatotoxic agents in sandwich (S) cultured mouse hepatocytes. **A)** Mouse hepatocytes were exposed to acetaminophen (APAP) at the indicated concentrations for 24h, either after 4h of plating (Day 0-1), or at day 1 after overnight starvation (Day 1-2) or at day 2 (Day 2-3). Cell toxicity was assessed by fluorescent microscopy using propidium iodide uptake as marker for necrotic cell death, visualized as red nuclei. Fluorescent pictures were merged with phase contras photographs. **B)** Quantification of cell death ratio (PI positive) versus total cells. The quantifications were performed in five random fields per condition, which included at least 100 cells. Bars show mean value of four independent experiments. Error bars = SEM.


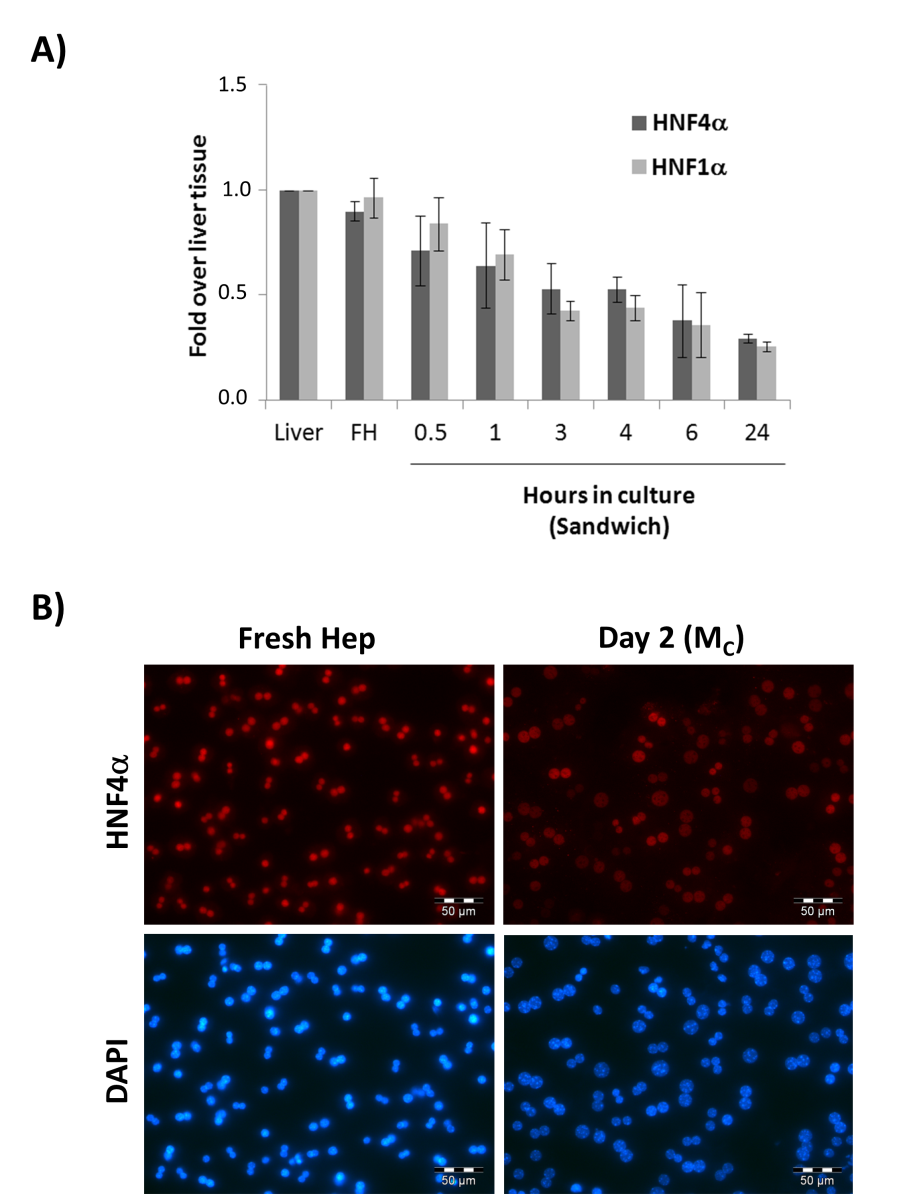


**Supplemental figure 12**: Downregulation of HNF1 and HNF4 in cultivated hepatocytes. **A)** qRT-PCR quantification of HNF4 and HNF1 mRNA expression in healthy mouse liver tissue (Liver), freshly isolated hepatocytes (FH), and in sandwich cultured hepatocytes for the indicated time. Data corresponds to three independent biological replicas. **B)** Fluorescence microscopy analysis of HNF4 (red) in freshly isolated hepatocytes (Fresh Hep) and in hepatocytes in monolayer culture (M_C_) for two days. HNF4 was detected by immunostaining with a rabbit anti HNF4 antibody, followed by a secondary Alexa 555 labeled antibody. Nuclei are visualized by blue fluorescence (DAPI).


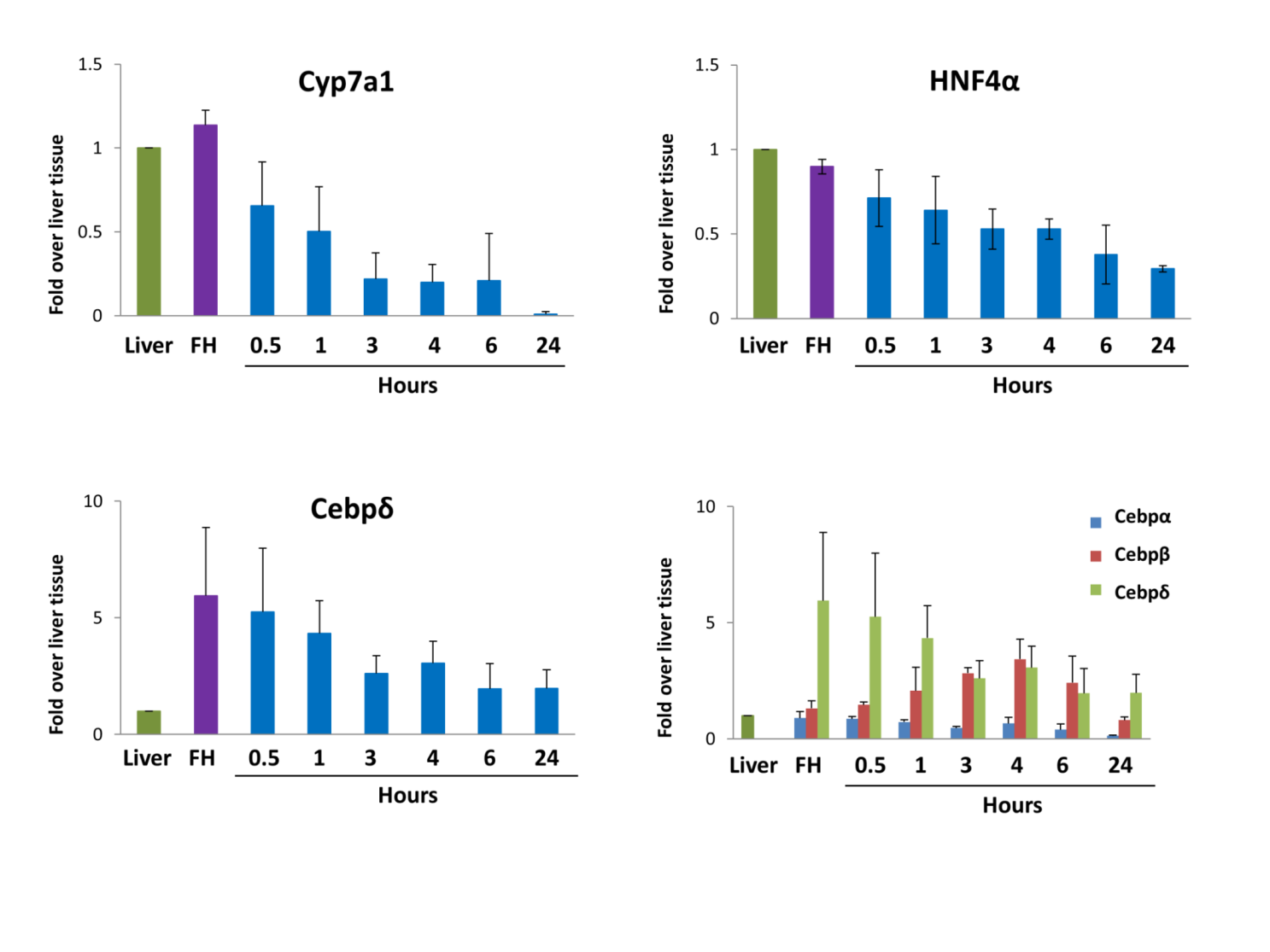


**Supplemental figure 13**: qRT-PCR quantification of Cebpα, β and δ in healthy mouse liver tissue (Liver), freshly isolated hepatocytes (FH) and hepatocytes in sandwich cultured hepatocytes for the indicated time. Data corresponds to average of three independent biological replicas. Error bars indicate standard error.


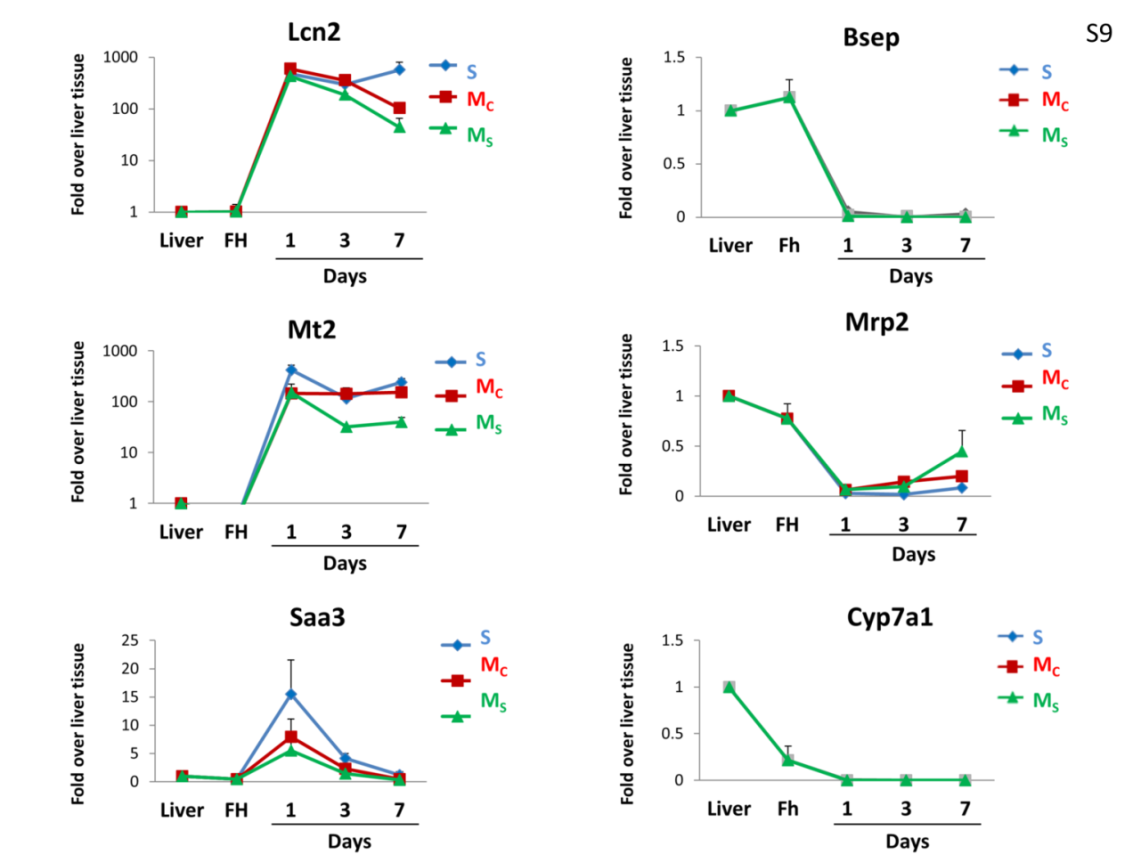


**Supplemental figure 14**: qRT-PCR quantification of the inflammation markers Lcn2, Mt2, Saa3 and the metabolism markers Bsep, Mrp2 and Cyp7a1 in healthy mouse liver tissue (Liver), freshly isolated hepatocytes (FH) and hepatocytes in sandwich (S), monolayer confluent (M_C_) and subconfluent (M_S_) cultures for the indicated time. Data corresponds to average of three independent biological replicas. Error bars indicate standard error.


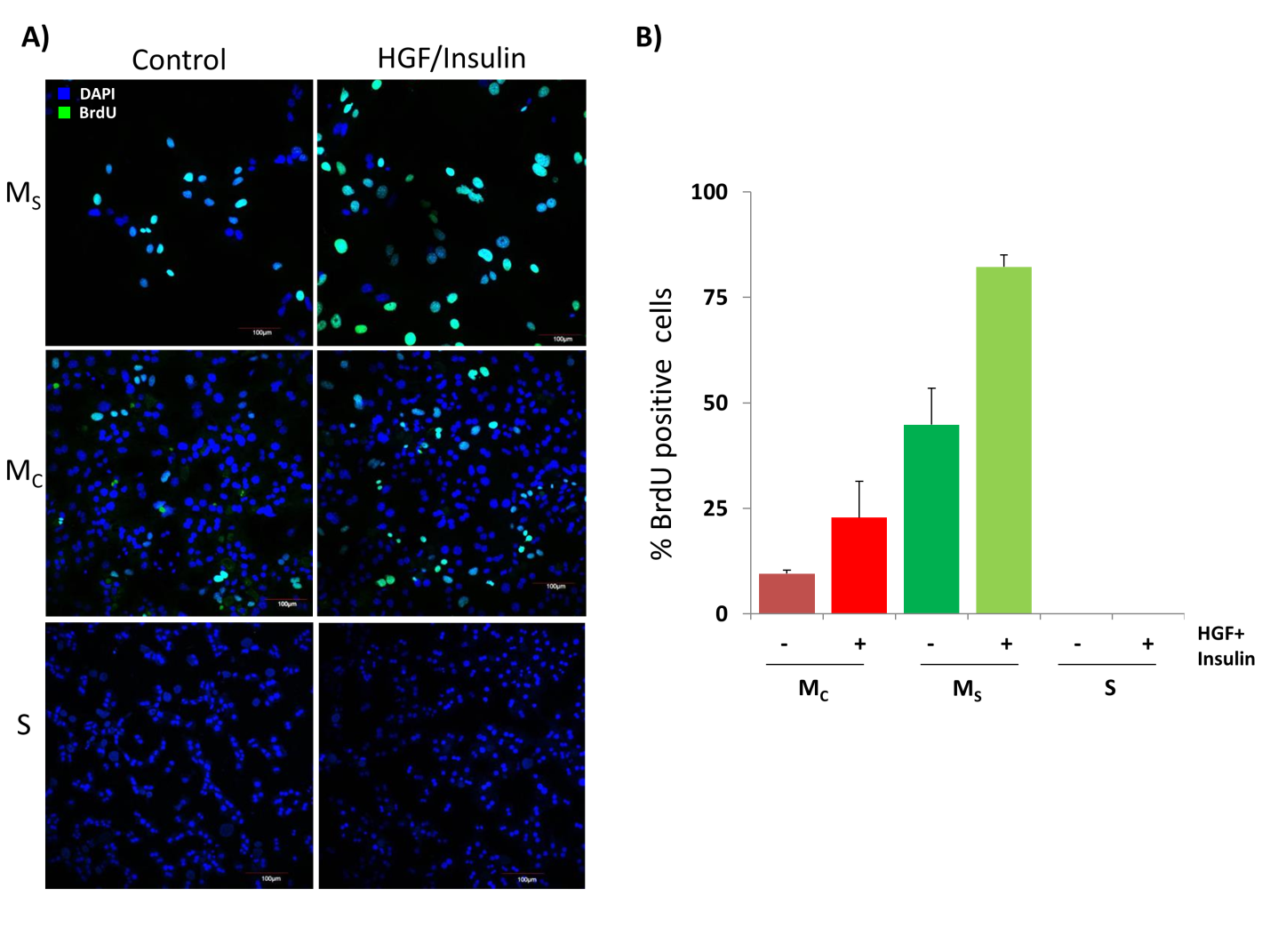


**Supplemental figure 15**: Growth factor-induced proliferation of primary mouse hepatocytes. **A)** Uptake of BrdU in hepatocytes after three days of culture in monolayer confluent (M_C_), monolayer subconfluent (M_S_) and sandwich conditions, in the presence of HGF (10 ng/ml) and insulin (10 ng/ml). Proliferating (S-phase) cells were detected by immunofluorescence using antibodies against BrdU (green). Nuclei were counterstained with DAPI (blue). **B)** Quantification of proliferating cells. The bars represent mean values of three independent experiments. Error bars correspond to standard error.


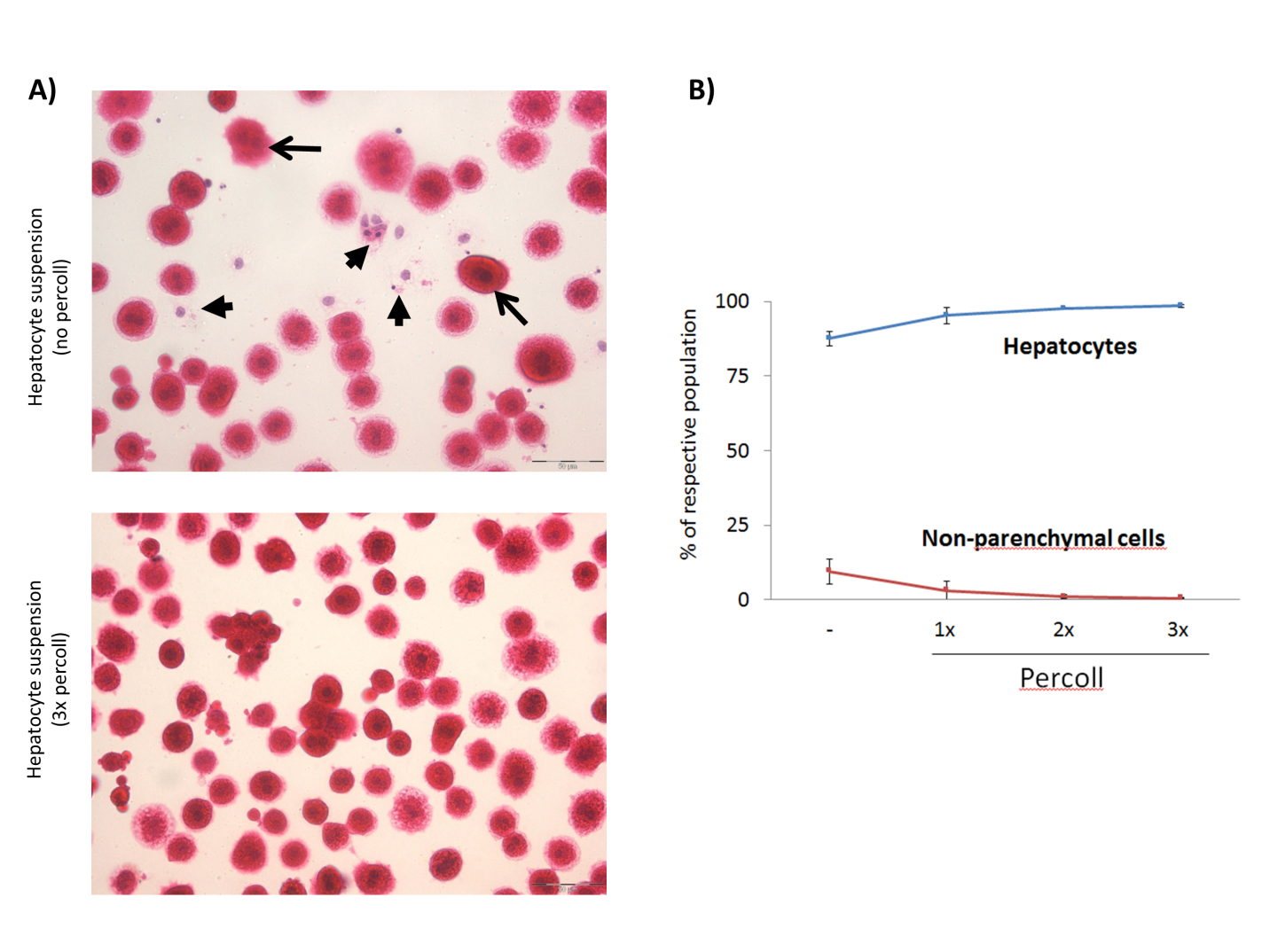


**Supplemental figure 16**: Assessment of non-parenchymal cell contamination in primary mouse hepatocyte suspension before and after enrichment with Percoll gradient. **A)** Bright field photographs of hematoxylin & eosin stained hepatocyte suspensions, without or with three Percoll purification steps. Hepatocytes are easily distinguished as large cells with intense red cytoplasm (arrows), whereas non-parenchymal cells appear with pale blue nuclei and faint cytoplasm (arrowheads). Notice how the non-parenchymal cell population is almost completely eliminated by three Percoll purification steps. **B)** Quantification of hepatocytes and non-parenchymal cells after serial Percoll purification steps. The cells were manually quantified using ten photographs of random fields. A minimum of one hundred cells were counted for each preparation. The data shows average of three independent preparations. Error bars indicate standard error.


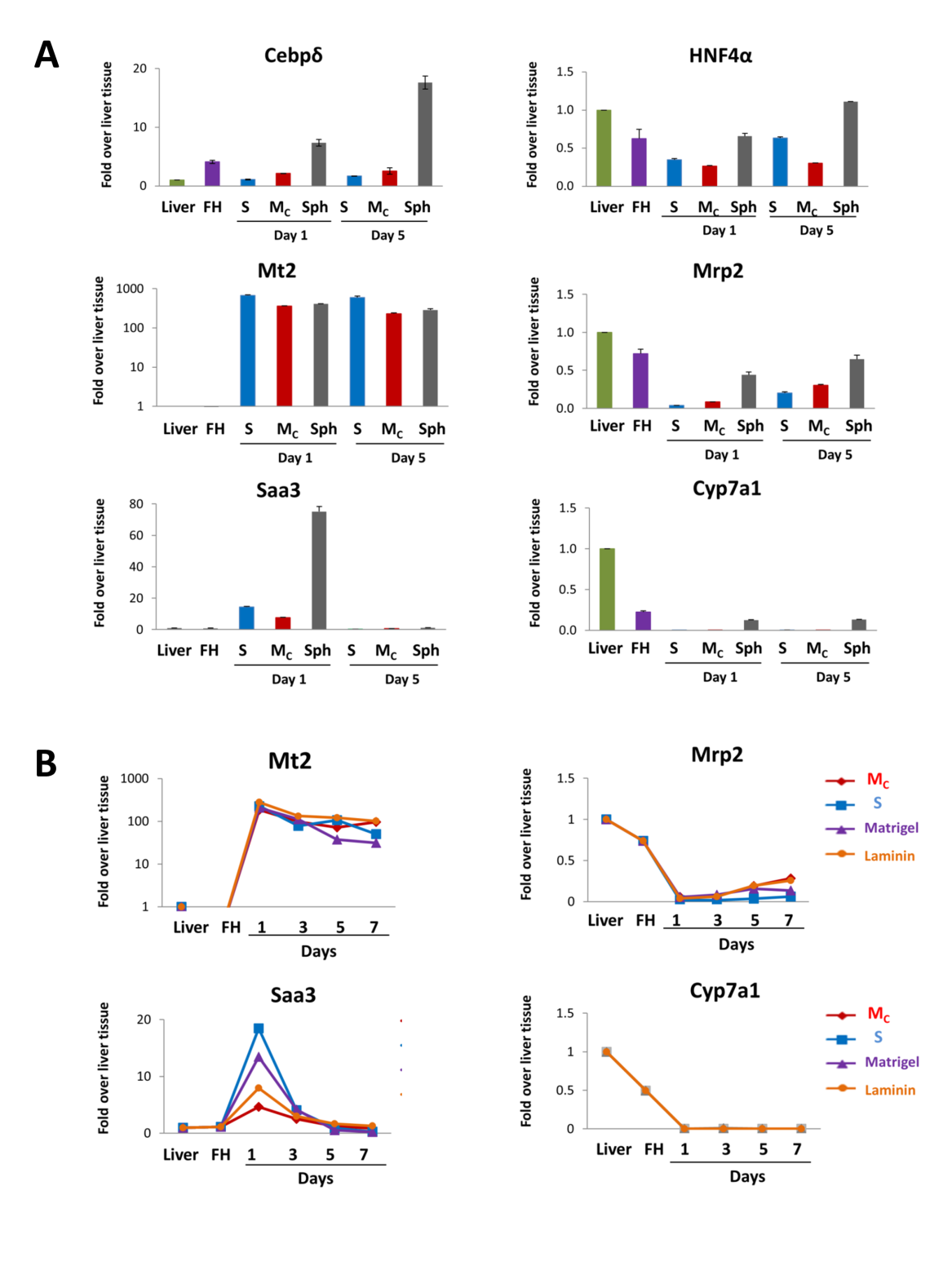


**Supplemental figure 17**: qRT-PCR quantification of the inflammation markers Cebpδ, Mt2, Saa3 and the metabolism markers HNF4α, Mrp2 and Cyp7a1 in healthy liver tissue (Liver), freshly isolated mouse hepatocytes (FH) and hepatocytes in sandwich (S), monolayer confluent (M_C_) cultures, or in spheroids (Sph) for the indicated time periods. Bars represent average of two independent experiments. Error bars indicate standard error.


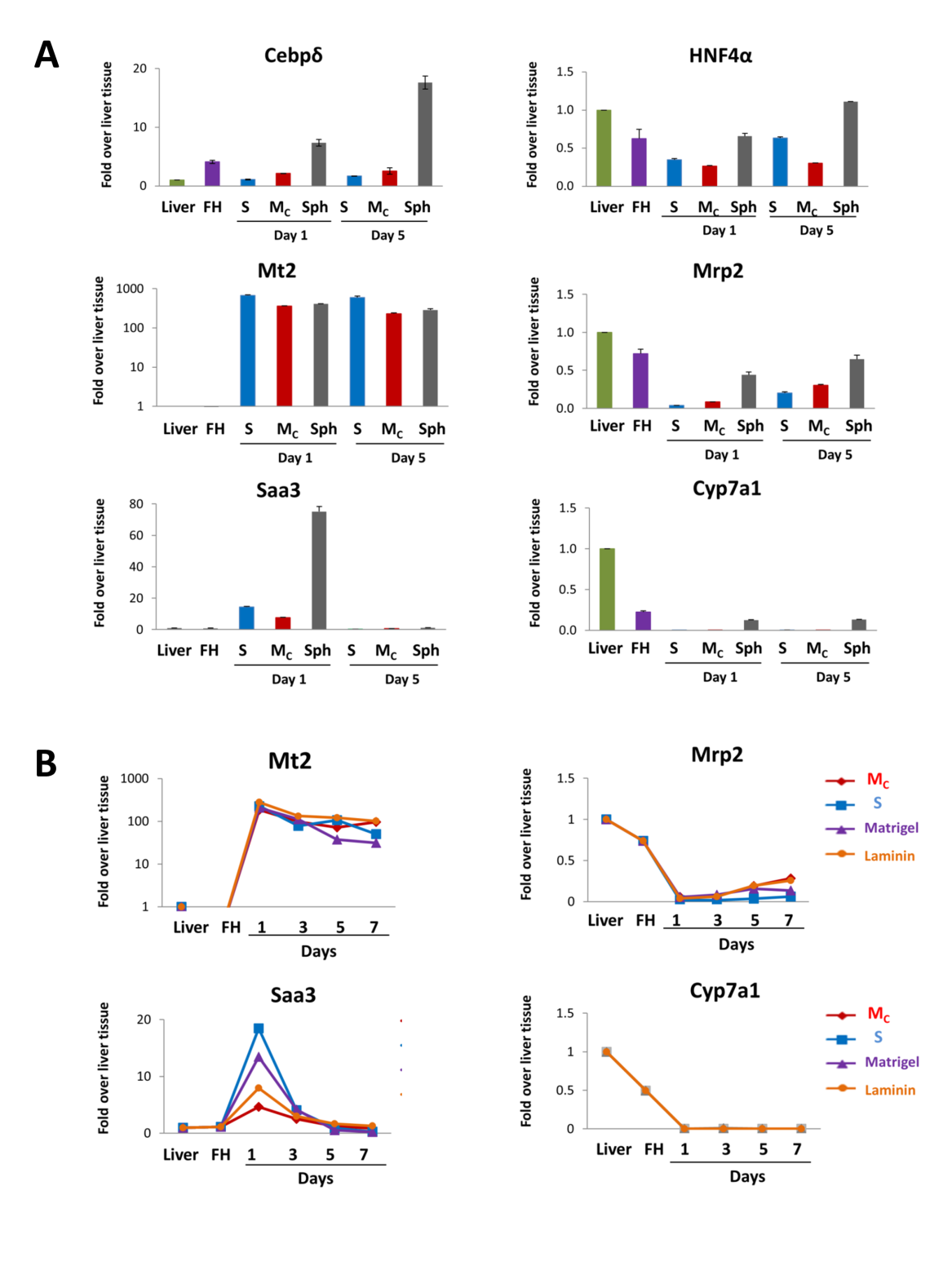


**Supplemental figure 18**: RT-qPCR quantification of the inflammation markers Mt2, Saa3 and the metabolism markers Mrp2 and Cyp7a1 in healthy liver tissue (Liver), freshly isolated mouse hepatocytes (FH) and hepatocytes in sandwich (S), monolayer confluent (M_C_) cultures, or cultured in matrigel or laminin for the indicated time.


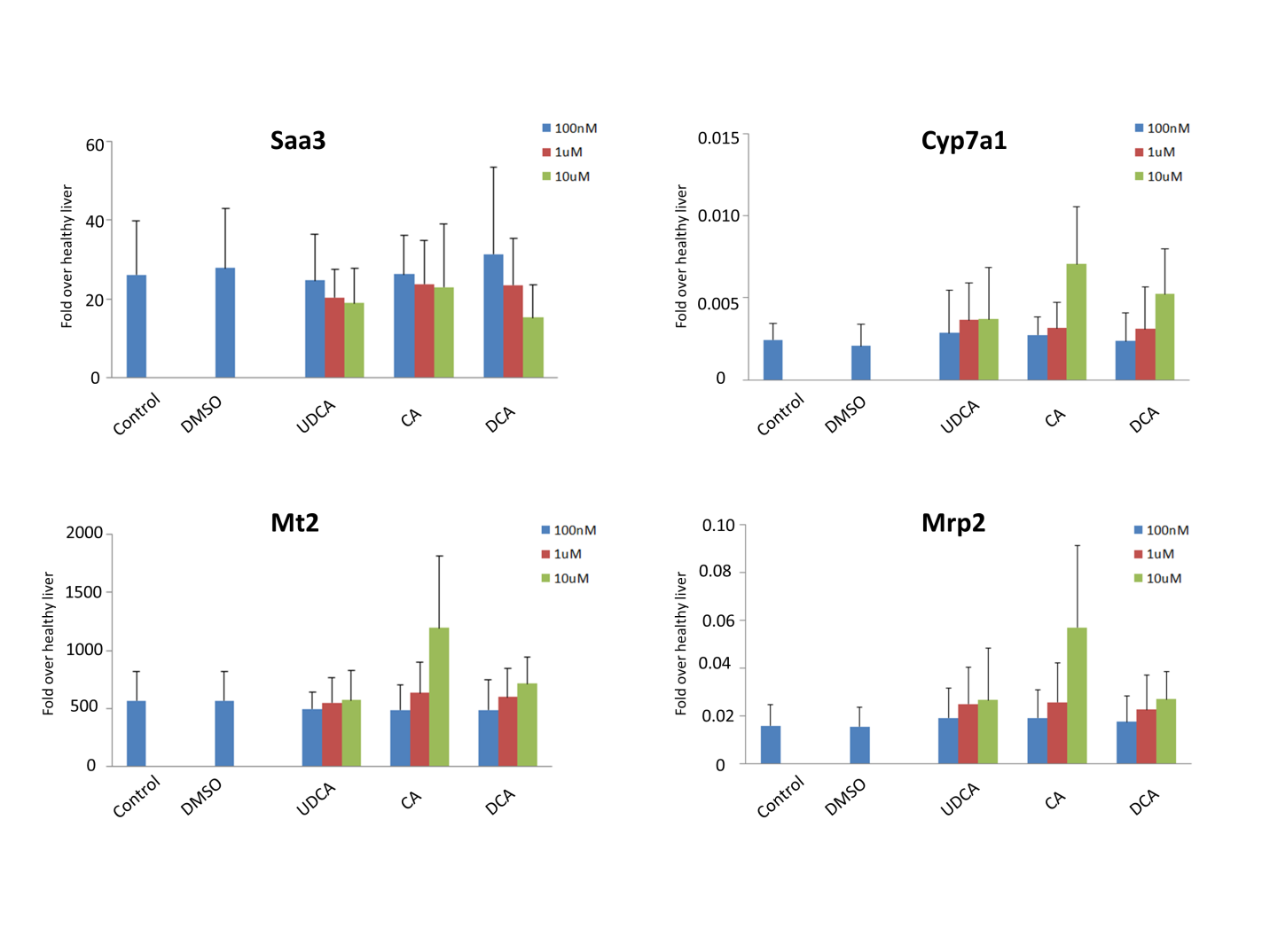


**Supplemental figure 19**: Real time qPCR analysis of the diagnostic genes for ´inflammation` (Saa3, Mt2) and ´mature liver functions` (Cyp7a1, Mrp2), in hepatocytes cultures on collagen monolayer using standard culture media (control) or additional bile salts for 24h at the indicated concentrations. DMSO was included as vehicle control. UCDA: ursodeoxycholic acid, CA: cholic acid, DCA: deoxycholic acid. Bars indicate mean values of three independent biological replicas ± SEM.


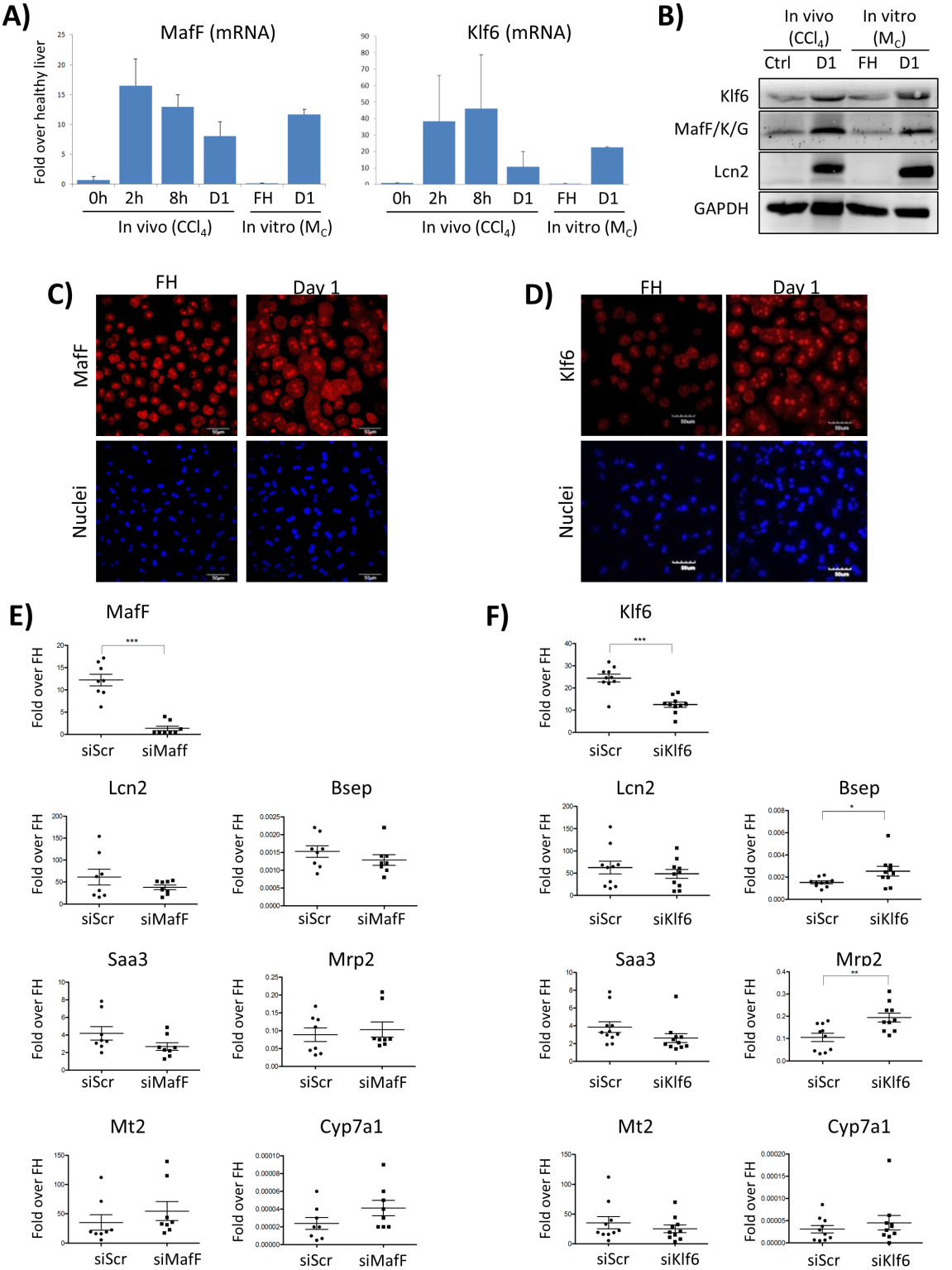


**Supplemental figure 20**: Robustness of transcriptional networks activated in stressed hepatocytes. **A-B)** The induction of the transcription factors MafF and Klf6 in cultivated primary mouse hepatocytes and in mouse liver after CCl_4_ intoxication was validated by real time qPCR (A) and western blot (B). The expression of Lcn2 was also assessed as a marker of inflammation. **C-D)** Both MafF and Klf6 were identified in hepatocyte nuclei by immunofluorescence and confocal microscopy. Nuclei were stained with DAPI. For MafF (D), nuclear translocation was observed already in freshly isolated hepatocytes and remained for the following 24h (Day 1). For Klf6 (D), nuclear translocation was strongly enhanced during the first 24h of culture (Day 1). **E-F)** siRNA-mediated knock down of the transcription factors MafF (E) and Klf6 (F), and the impact on diagnostic genes for the ´inflammation` (Lcn2, Saa2 and Mt2) and ´mature liver functions` (Bsep, Mrp2 and Cyp7a1) clusters, respectively. Dot plots correspond to fold of expression of the corresponding genes in monolayer cultured mouse hepatocytes, 72 hours after transfection of siRNA oligos. Bars indicate mean value (n = 5), whiskers represent SEM.


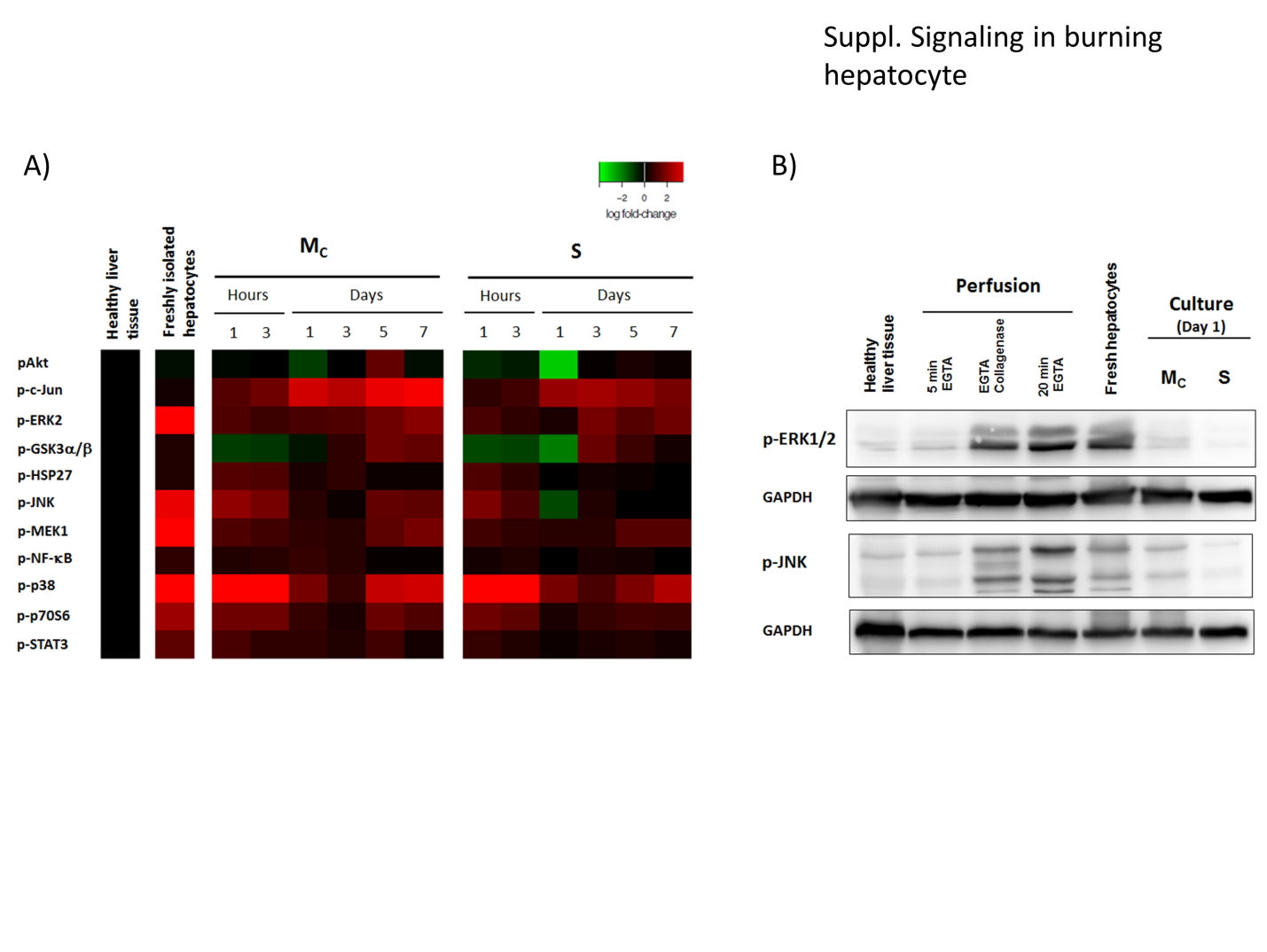


**Supplemental figure 21**: **A)** Phosphorylation of signal transduction pathway-associated proteins was performed using LUMINEX multiplex beads, comparing healthy mouse liver tissue, freshly isolated mouse hepatocytes, and mouse hepatocytes in monolayer confluent (M_C_) or sandwich (S) culture. The heatmap represents mean values of signal intensity in freshly isolated and cultivated mouse hepatocytes, compared to healthy liver tissue (n= 3 biological replicas). **B)** Western blot analysis of phosphorylated ERK1/2, JNK and total GAPDH as loading control. The protein extracts were collected from fresh healthy liver tissue, livers after 5 minutes of EGTA buffer perfusion (5 min EGTA), 5 minutes EGTA buffer plus 15 minutes collagenase buffer perfusion (EGTA + Coll.), or 20 minutes of EGTA buffer perfusion. Protein extracts were also collected from freshly isolated hepatocytes and from hepatocytes in monolayer confluent (M_C_) or sandwich (S) culture on day 1. Representative of two independent experiments.


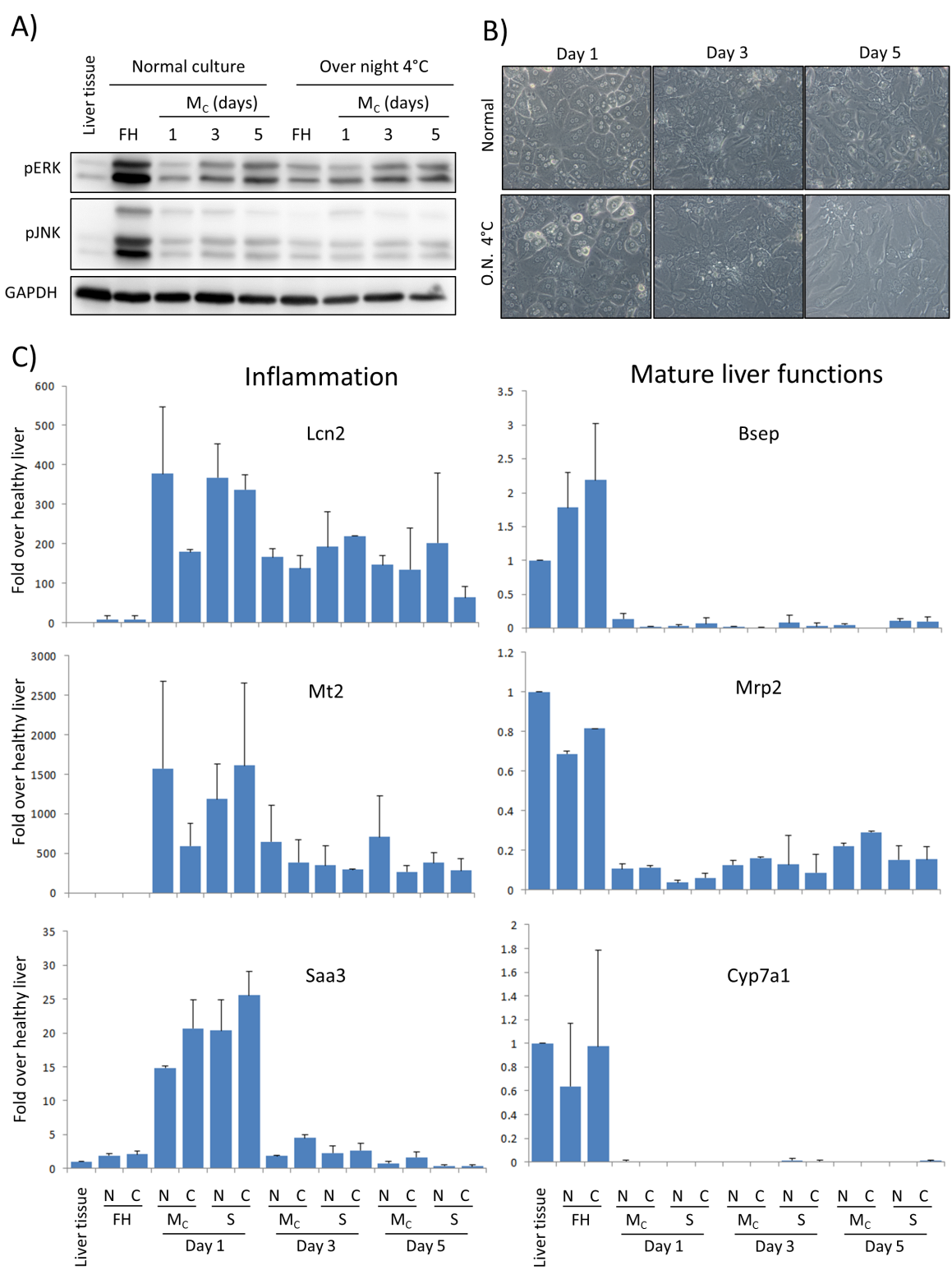


**Supplemental figure 22**: Effect of cold storage in signaling pathway activity and gene expression in primary mouse hepatocytes. The analyses were performed using a single hepatocyte preparation, which was split into two fractions. For the ´normal culture` condition, hepatocytes were treated according to standard procedures, while a fraction of hepatocytes was stored at 4°C (´over night at 4°C`). Both hepatocyte fractions were subsequently cultured on monolayer confluent conditions for the indicated time. **A)** Western blot analysis of ERK and JNK phosphorylation in mouse hepatocytes in suspension (FH) and in monolayer confluent culture for the indicated time. The analysis shows activation levels on hepatocytes in conventional culture and after 24h of storage at 4°C (representative of two independent experiments). GAPDH was used as loading control. **B)** Phase contrast images of mouse hepatocytes on monolayer culture for the indicated time. No signs of cell death were observed on hepatocytes plated after overnight storage at 4°C. **C)** Quantitative real time PCR analysis of the diagnostic genes for the ´inflammation` (Lcn2, Saa2 and Mt2) and ´mature liver functions` (Bsep, Mrp2 and Cyp7a1) clusters, respectively. The analysis was performed on cell extracts from hepatocytes cultured using standard conditions (normal = N) and after overnight storage at 4°C (cooling = C) for the indicated time. Bars show mean value of two independent experiments. Error bars = SEM.
